# Supplementary material for: Single-cell and spatial transcriptomic analyses of gene therapy-associated retinal inflammation in non-human primates
Source: Mol Ther Adv. 2026 Mar 30;34(2):201726. doi: 10.1016/j.omta.2026.201726 (PMC13148909; doi:10.1016/j.omta.2026.201726)
Supplement: Document S1. Figures S1–S15 and Tables S1 and S2 [file mmc1.pdf]

OMTA, Volume *34*

Supplemental information

Single-cell and spatial transcriptomic analyses of gene therapy-associated retinal inflammation in non-human primates

Célia Sourd, Joel Quinn, Molly C. John, Cristina Martinez-Fernandez de la Camara, Lakshanie C. Wickramasinghe, Moustafa Attar, Hoda Shamsnajafabadi, Ahmed Salman, Sally A. Cowley, Calliope A. Dendrou, Robert E. MacLaren, Jasmina Cehajic-Kapetanovic, and Kanmin Xue

Table S1. List of PCR primers.

Name	Sequence	Purpose
**mScarlet_F**	GCGTGATGAACTTCGAGGAC	qPCR Titration of Vector
**mScarlet_R**	CTTGTAGATCAGGGTGCCGT	qPCR Titration of Vector

Table S2. List of antibodies.

Target	Host	Clonality	Reference	Supplier	Working dilution
**RPGR**	Rabbit	Polyclonal	HPA001593	Sigma-Aldrich	1:200 (IHC)
**RPE65**	Mouse	Monoclonal	401.8B11.3D9	Novus Biologicals	1:250 (IHC)
**IBA1**	Rabbit	Monoclonal	019-19741	Wako	1:500 (IHC)
**GFAP**	Chicken	Polyclonal	Ab4674	Abcam	1:200 (IHC)
**CD45**	Rabbit	Monoclonal	Ab281586	Abcam	1:250 (IHC)

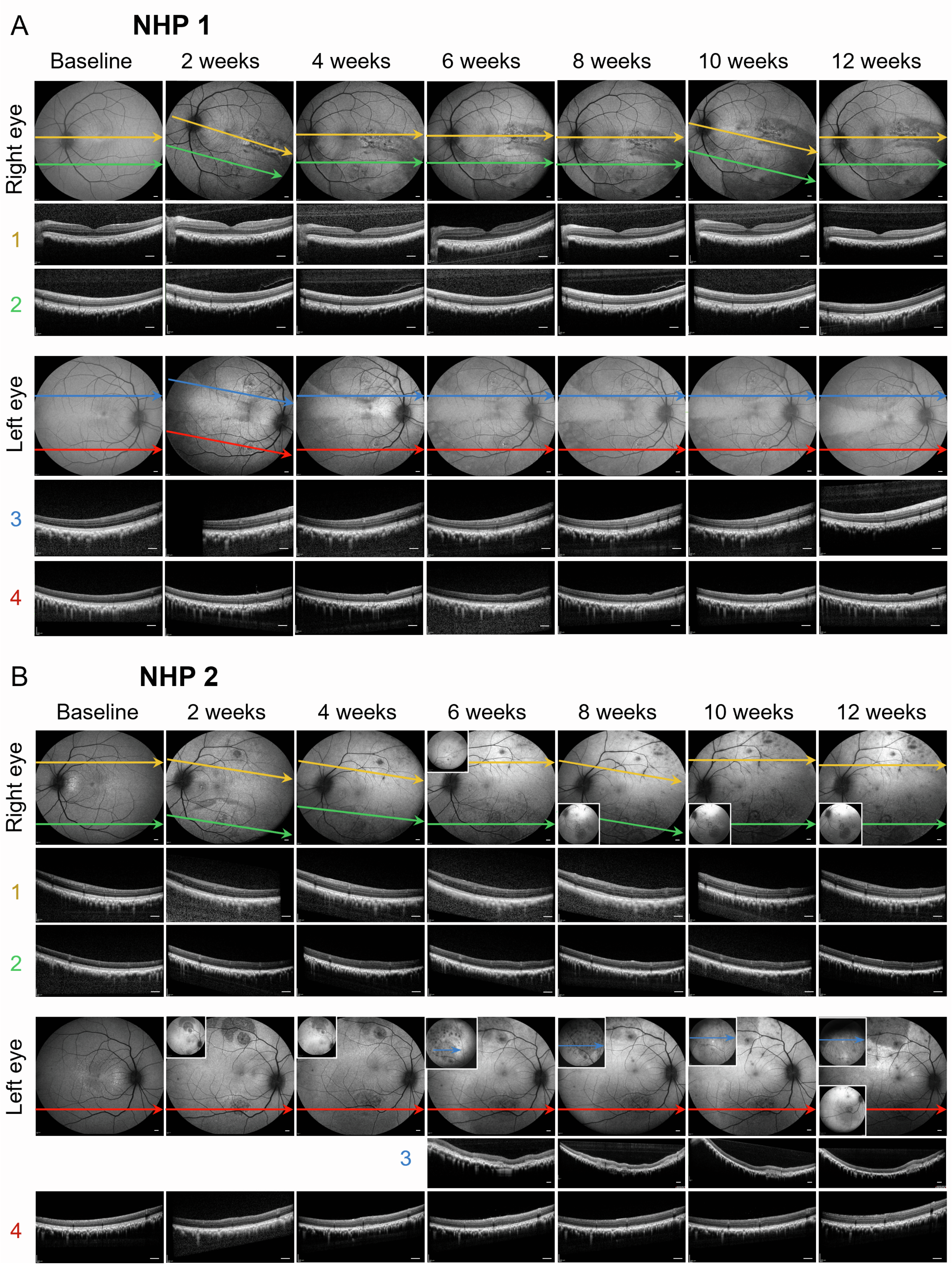
A
NHP 1
Baseline
2 weeks
4 weeks
6 weeks
8 weeks
10 weeks
12 weeks
Right eye
1
2
Left eye
3
4
B
NHP 2
Baseline
2 weeks
4 weeks
6 weeks
8 weeks
10 weeks
12 weeks
Right eye
1
2
Left eye
3
4

Figure S1. Longitudinal multimodal retinal imaging over 12 weeks following subretinal injection of AAV vectors in NHPs.

See Figure 2. Longitudinal multimodal retinal imaging of the macula following subretinal injection of AAV vectors over 12 weeks of both eyes of NHP1 (**A**) and NHP2 (**B**). The coloured lines represent the locations of OCT sections mapped to the fundus autofluorescence (AF) images from inside the treated subretinal bleb areas. Note that all retinal images are vertically inverted (top of the image representing inferior retina). Hypo-autofluorescent patches corresponding to the development of RPE/outer retinal atrophy can be seen along the inferior arcade of the left eye of NHP2 which was treated with an AAV8-GRK1-*mScarlet* vector. The surrounding region showed increased background hyper-autofluorescence which likely represents mScarlet reporter expression. Scale bars = 500 μm.

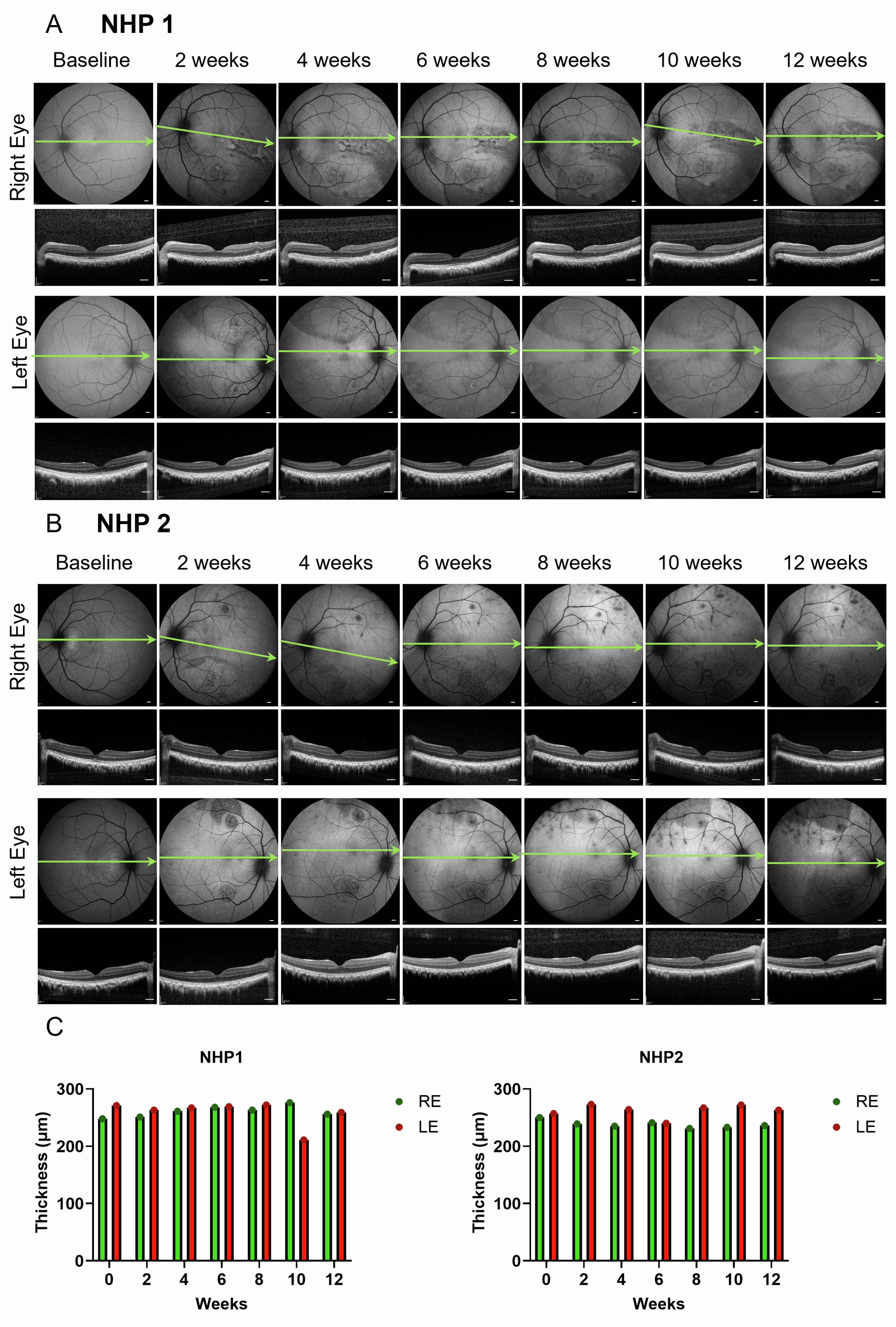
A
NHP 1
Baseline
2 weeks
4 weeks
6 weeks
8 weeks
10 weeks
12 weeks
Right Eye
Left Eye
B
NHP 2
Baseline
2 weeks
4 weeks
6 weeks
8 weeks
10 weeks
12 weeks
Right Eye
Left Eye
C
NHP1
NHP2
Thickness (µm)
300
200
100
0
0
2
4
6
8
10
12
Weeks
RE
LE

Figure S2. Preservation of macula anatomy following subretinal AAV gene therapy in NHPs.

Fundus autofluorescence (AF) and OCT imaging of the macula following subretinal injection of AAV vectors over 12 weeks of the right and left eyes of NHP1 (**A**) and NHP2 (**B**). Green lines on the AF images represent the localisations of corresponding OCT sections across the fovea. White bars represent a scale of 500µm. (**C**) Retinal thickness at the macula of NHP1 and NHP2 derived from the OCT images showed no significant changes over 12 weeks.

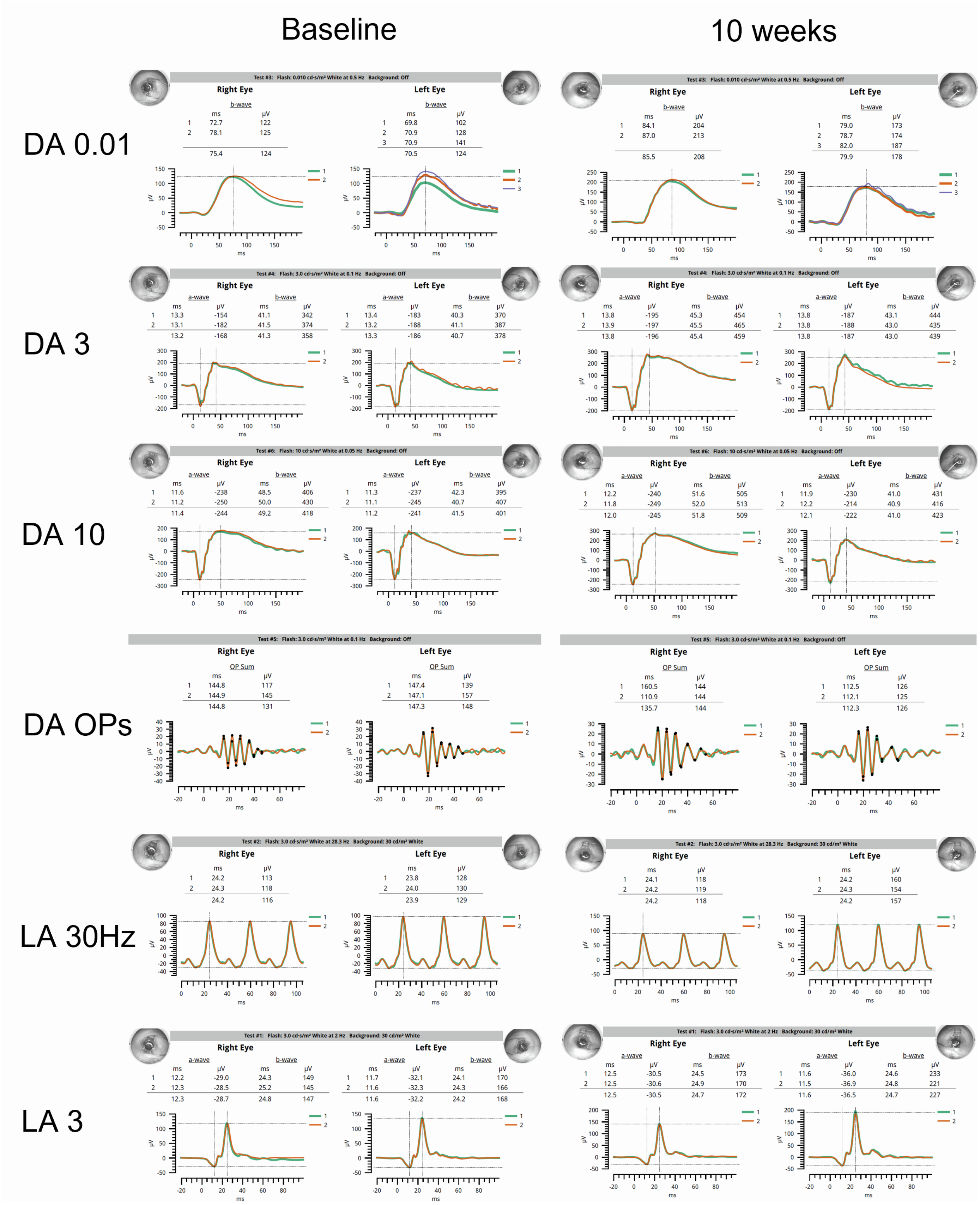
Baseline
10 weeks
DA 0.01
DA 3
DA 10
DA OPs
LA 30Hz
LA 3
Right Eye
Left Eye

Figure S3. Electroretinogram of NHP1.

Electroretinogram (ERG) responses recorded under dark-adapted (DA) and light-adapted (LA) conditions at baseline and 10 weeks after treatment in the right and left eyes of NHP1. Measurements included standard a-wave and b-wave responses, as well as oscillatory potentials (OPs) and 30 Hz flicker were performed in duplicates (green and orange lines) to assess retinal function.

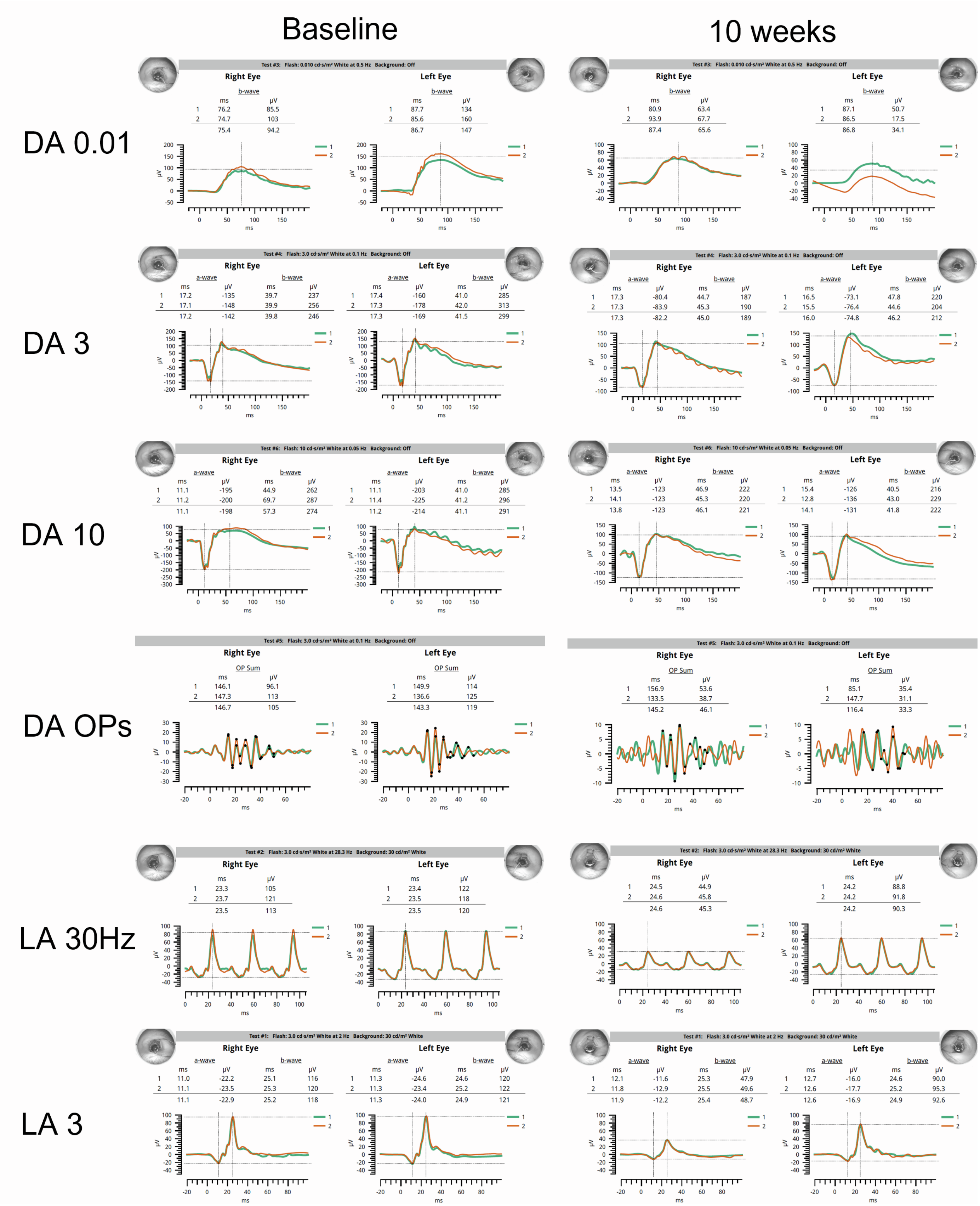
Baseline
10 weeks
DA 0.01
DA 3
DA 10
DA OPs
LA 30Hz
LA 3

Figure S4. Electroretinogram of NHP2.

Electroretinogram (ERG) responses recorded under dark-adapted (DA) and light-adapted (LA) conditions at baseline and 10 weeks after treatment in the right and left eyes of NHP2. Measurements included standard a-wave and b-wave responses, as well as oscillatory potentials (OPs) and 30 Hz flicker were performed in duplicates (green and orange lines) to assess retinal function.

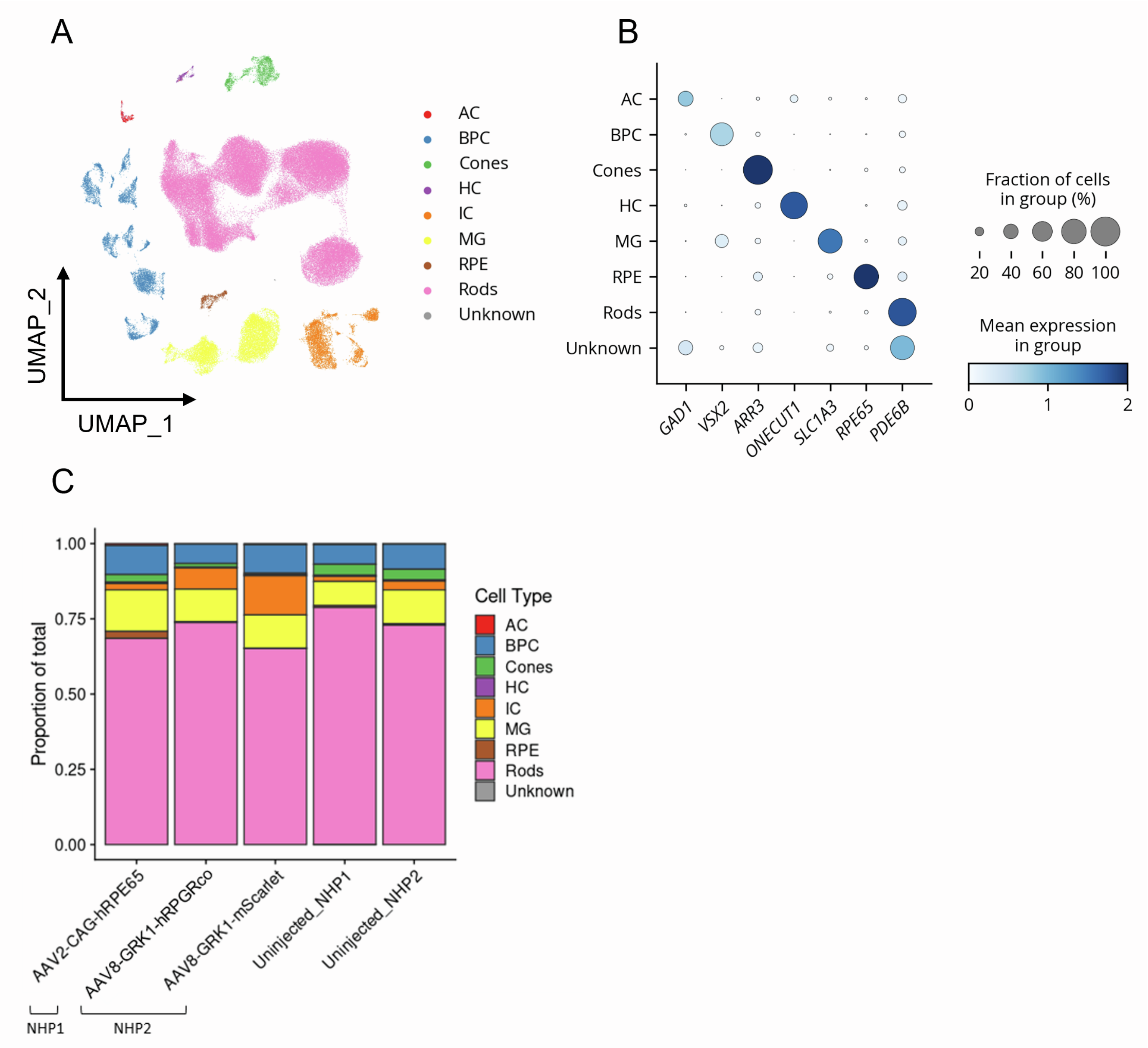

Figure S5. Single-cell transcriptomic analysis of retina cell populations in treated and untreated retinas.

Single-cell RNAseq data from dissociated retinas of NHP1 and NHP2 were combined. (**A**) Integrated UMAP of the entire cell population with labelled cell types. (**B**) Major marker genes used for cell type annotation. (**C**) Proportion of cell types identified from each AAV vector treated bleb area. AC = amacrine cells; BPC = bipolar cells; HC = horizontal cells; IC = immune cells; MG = Müller glia; RPE = retinal pigment epithelium.

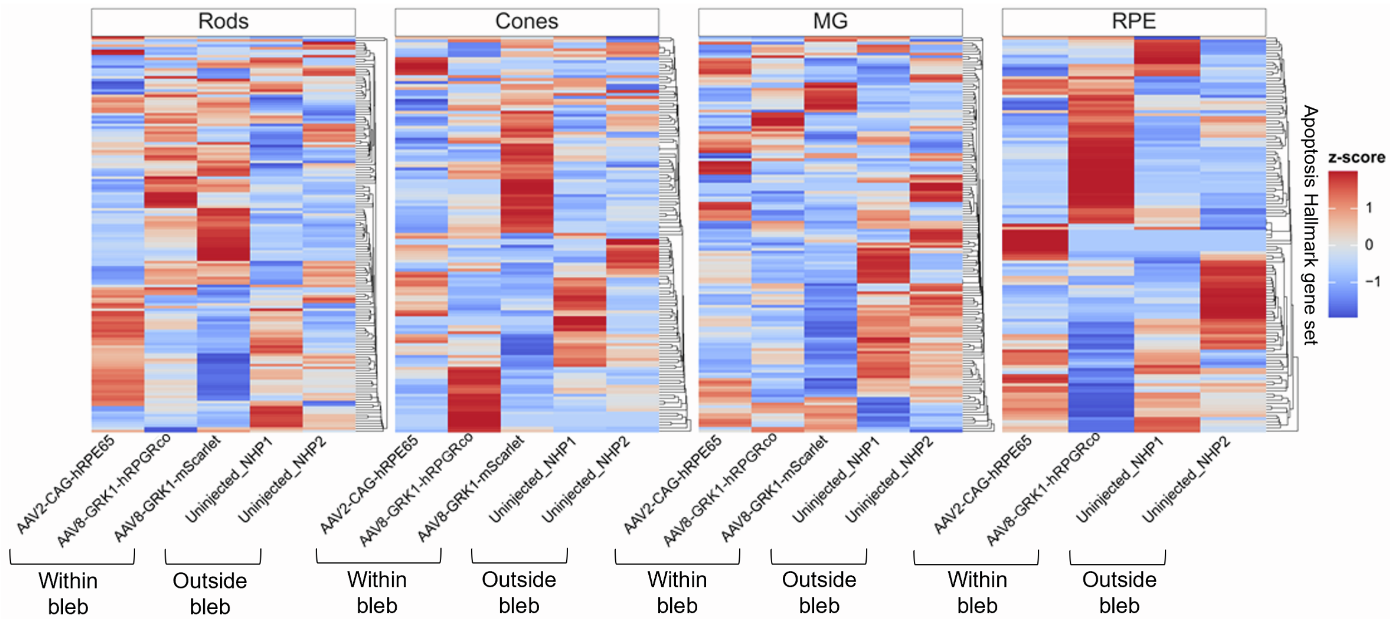

Figure S6. No clear correlation found between the levels of apoptotic marker gene expression and AAV treatment.

Normalised expression levels of apoptotic marker genes in the Apoptosis MSigDB Hallmark gene set in the rod, cone, Müller glia and RPE cell populations were compared between AAV treated (within bleb) versus untreated (outside bleb) retinas.

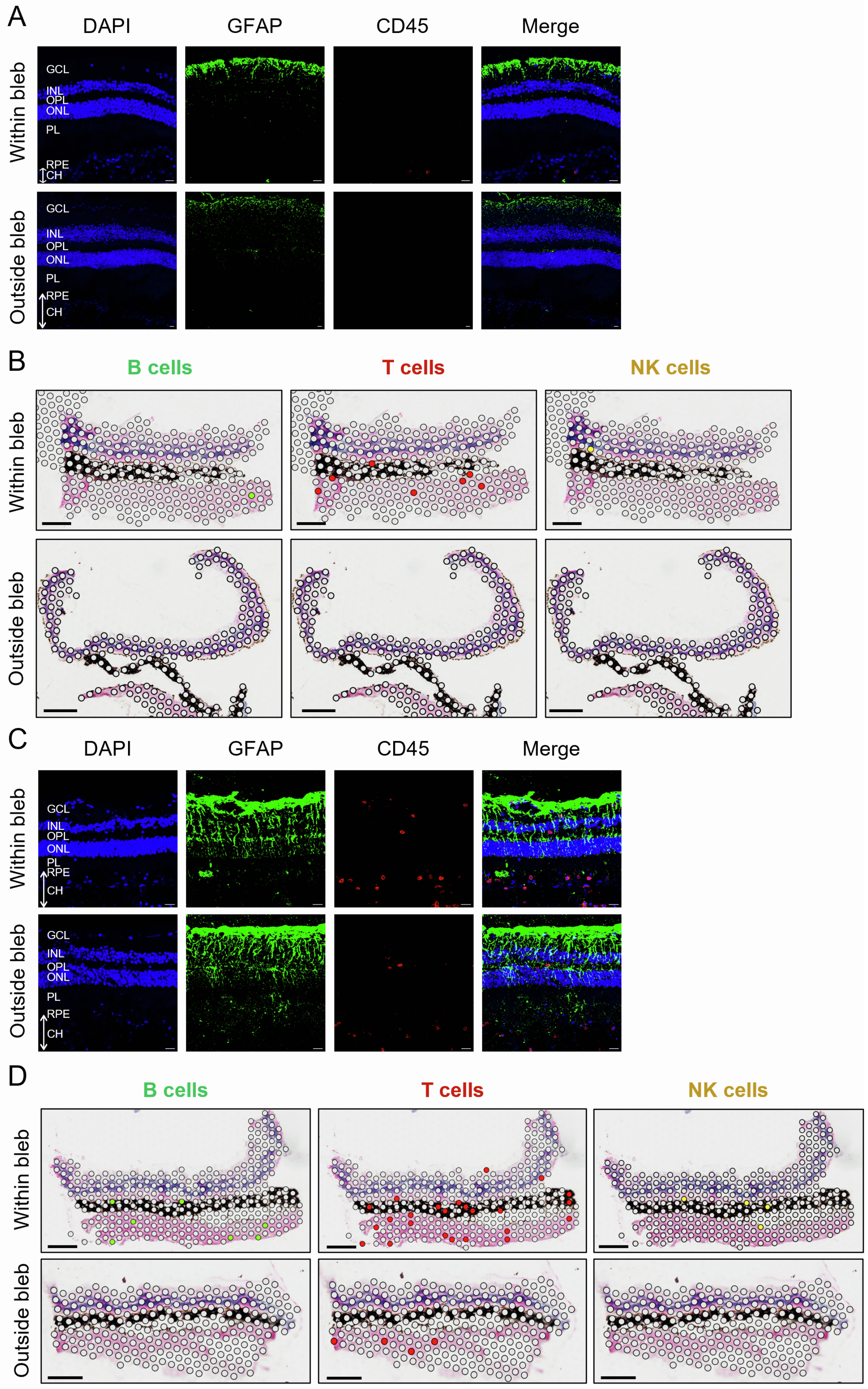
A
DAPI
GFAP
CD45
Merge
Within bleb
Outside bleb
GCL
INL
OPL
ONL
PL
RPE
CH
B
B cells
T cells
NK cells
Within bleb
Outside bleb
C
DAPI
GFAP
CD45
Merge
Within bleb
Outside bleb
GCL
INL
OPL
ONL
PL
RPE
CH
D
B cells
T cells
NK cells
Within bleb
Outside bleb

Figure S7. Analysis of inflammation in the AAV-treated NHP retinas.

(**A, C**) Immunostaining of retina sections from (**A**) AAV2-CAG-*hRPE65* (NHP1) and (**C**) AAV8-GRK1-*hRPGRco* (NHP2) for GFAP and CD45 proteins. Control sections were taken from outside the treated blebs. Scale bars = 20μm. GCL = ganglion cell layer; INL = inner nuclear layer; OPL = outer plexiform layer; ONL = outer nuclear layer; PL = photoreceptor layer; RPE = retinal pigmented epithelium. (**B, D**) Spatial transcriptomic maps of B cell, T cell and natural killer (NK) cell clusters within retina sections of NHP1 (**B**) and NHP2 (**D**). Coloured spots represent the locations of cells expressing marker genes of interest overlayed on the H&E staining image. Scale bar = 0.5 mm.

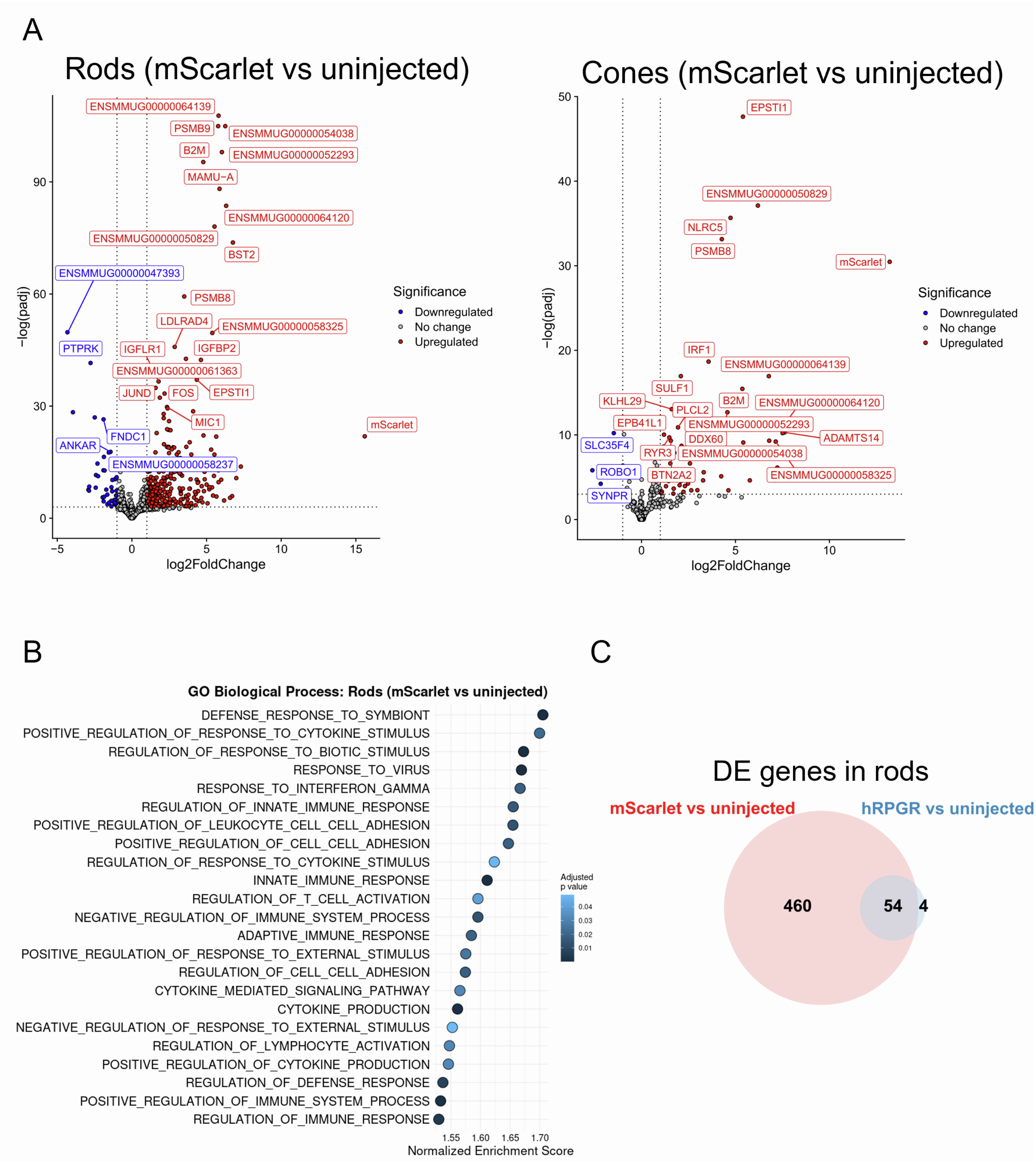
A
Rods (mScarlet vs uninjected)
ENSMMUG00000064139
PSMB9
ENSMMUG00000054038
B2M
ENSMMUG00000052293
MAMU−A
ENSMMUG00000064120
ENSMMUG00000050829
BST2
ENSMMUG00000047393
PSMB8
LDLRAD4
ENSMMUG00000058325
PTPRK
IGFLR1
IGFBP2
ENSMMUG00000061363
JUND
FOS
EPSTI1
MIC1
mScarlet
FNDC1
ANKAR
ENSMMUG00000058237
−log(padj)
log2FoldChange
Significance
Downregulated
No change
Upregulated
Cones (mScarlet vs uninjected)
EPSTI1
ENSMMUG00000050829
NLRC5
PSMB8
mScarlet
IRF1
ENSMMUG00000064139
SULF1
KLHL29
B2M
ENSMMUG00000064120
PLCL2
EPB41L1
ENSMMUG00000052293
SLC35F4
DDX60
ADAMTS14
RYR3
ENSMMUG00000054038
ROBO1
BTN2A2
ENSMMUG00000058325
SYNPR
B
GO Biological Process: Rods (mScarlet vs uninjected)
DEFENSE_RESPONSE_TO_SYMBIONT
POSITIVE_REGULATION_OF_RESPONSE_TO_CYTOKINE_STIMULUS
REGULATION_OF_RESPONSE_TO_BIOTIC_STIMULUS
RESPONSE_TO_VIRUS
RESPONSE_TO_INTERFERON_GAMMA
REGULATION_OF_INNATE_IMMUNE_RESPONSE
POSITIVE_REGULATION_OF_LEUKOCYTE_CELL_CELL_ADHESION
POSITIVE_REGULATION_OF_CELL_CELL_ADHESION
REGULATION_OF_RESPONSE_TO_CYTOKINE_STIMULUS
INNATE_IMMUNE_RESPONSE
REGULATION_OF_T_CELL_ACTIVATION
NEGATIVE_REGULATION_OF_IMMUNE_SYSTEM_PROCESS
ADAPTIVE_IMMUNE_RESPONSE
POSITIVE_REGULATION_OF_RESPONSE_TO_EXTERNAL_STIMULUS
REGULATION_OF_CELL_CELL_ADHESION
CYTOKINE_MEDIATED_SIGNALING_PATHWAY
CYTOKINE_PRODUCTION
NEGATIVE_REGULATION_OF_RESPONSE_TO_EXTERNAL_STIMULUS
REGULATION_OF_LYMPHOCYTE_ACTIVATION
POSITIVE_REGULATION_OF_CYTOKINE_PRODUCTION
REGULATION_OF_DEFENSE_RESPONSE
POSITIVE_REGULATION_OF_IMMUNE_SYSTEM_PROCESS
REGULATION_OF_IMMUNE_RESPONSE
Adjusted p value
Normalized Enrichment Score
C
DE genes in rods
mScarlet vs uninjected
hRPGR vs uninjected
460
54
4

Figure S8. Upregulation of antiviral and MHC Class I genes in NHP photoreceptors after AAV gene therapy.

Results derived from single-cell RNAseq analysis of dissociated retinas from NHP2. (**A**) DESeq2 differential expression analysis. Differentially upregulated genes include B2M (MHC I light chain), ENSMMUG00000054038 (macaque MHC I antigen), ENSMMUG00000064120 (ortholog to CD1D, MHC-like lipid antigen presenter), ENSMMUG00000050829 (MHC I pathway regulator), ENSMMUG00000052293 (ortholog to KIAA1109, MHC I complex assembly) and ENSMMUG00000058325 (MHC I antigen), indicating upregulated MHC class I antigen presentation. Upregulation of multiple immune system-associated genes in rods and cones from AAV8-GRK1-*mScarlet*-treated bleb versus untreated (outside of bleb) retina sections. (**B**) Gene Ontology (GO) Biological Process enrichment of upregulated genes in rods. A variety of antiviral and other immune-related gene sets in AAV8-GRK1-mScarlet treated bleb areas can be observed when compared with untreated (outside of bleb) retina sections. (**C**) Venn diagram comparing differentially expressed genes in rods between AAV8-GRK1-*hRPGRco* and AAV8-GRK1-*mScarlet* treated retinas. The majority of upregulated genes in rods from AAV8-GRK1-*hRPGRco* treated retina overlapped with those from AAV8-GRK1-*mScarlet* treated retina, suggesting a similar response. DE genes = differently expressed genes.

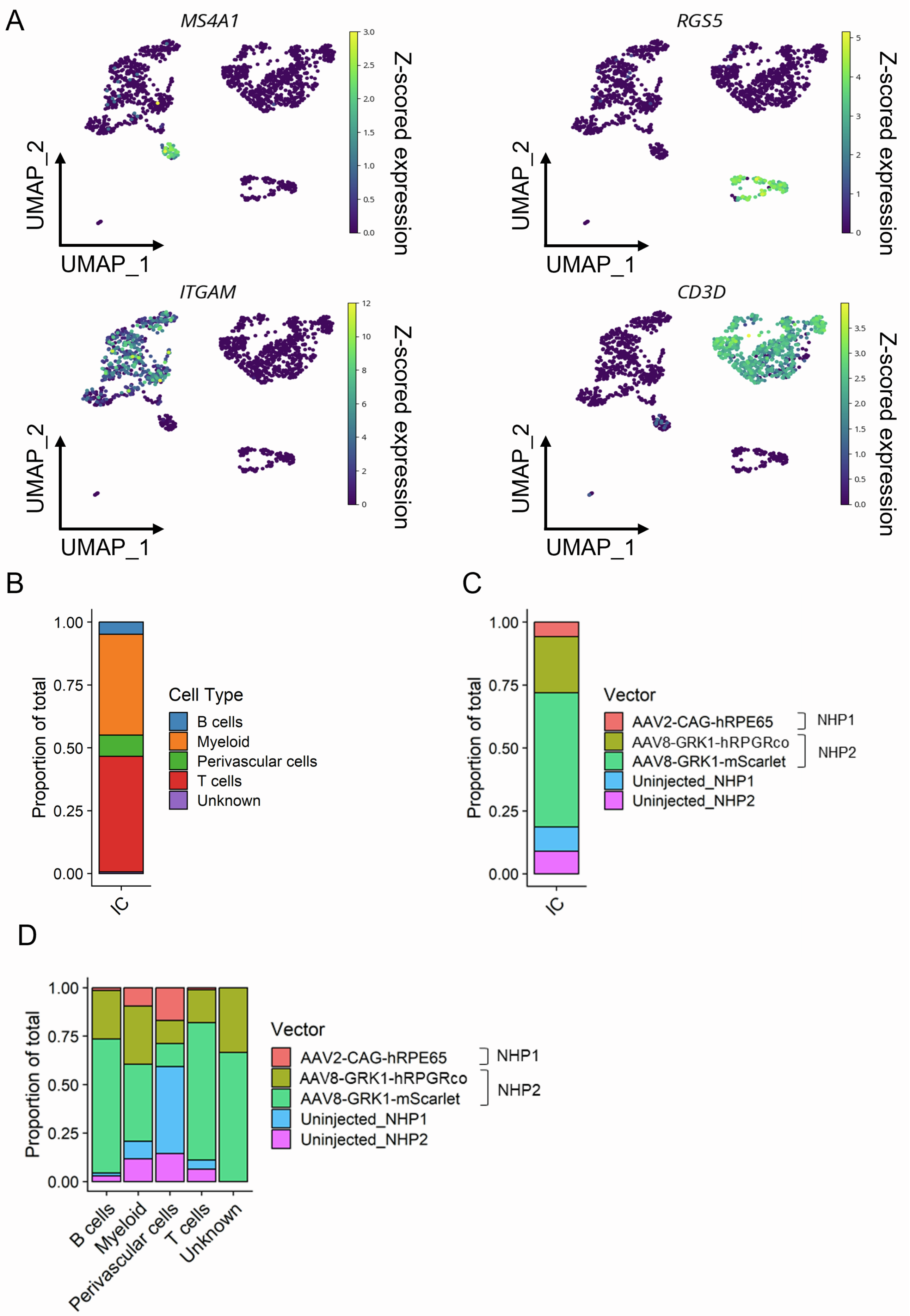
A
MS4A1
RGS5
ITGAM
CD3D
UMAP_1
UMAP_2
Z-scored expression
B
Proportion of total
Cell Type
B cells
Myeloid
Perivascular cells
T cells
Unknown
IC
C
Vector
AAV2-CAG-hRPE65
AAV8-GRK1-hRPGRco
AAV8-GRK1-mScarlet
Uninjected_NHP1
Uninjected_NHP2
NHP1
NHP2
D
1.00
0.75
0.50
0.25
0.00

Figure S9. The immune cell population in AAV-injected NHP retinas.

See Figure 5 A. (**A**) Marker genes used to identify B cells (*MS4A1*), perivascular cells (*RGS5*), myeloid cells (*ITGAM*) and T cells (*CD3D*). Data from both NHP1 and NHP2. (**B**) Relative proportions of different immune cell populations showing predominantly myeloid and T cell infiltrate. (**C**) Relative contributions of immune cells from each retina sample showing that AAV8-GRK1-*mScarlet* and AAV8-GRK1-*hRPGRco* treated retinas contributed the majority of immune cells. (**D**) Relative contributions from different retina samples for each immune cell type. The majority of B cells, T cells and myeloid cells came from AAV8-GRK1-*mScarlet* and AAV8-GRK1-*hRPGRco* treated retinas, indicating ongoing adaptive immune response in the treated retina of NHP2.

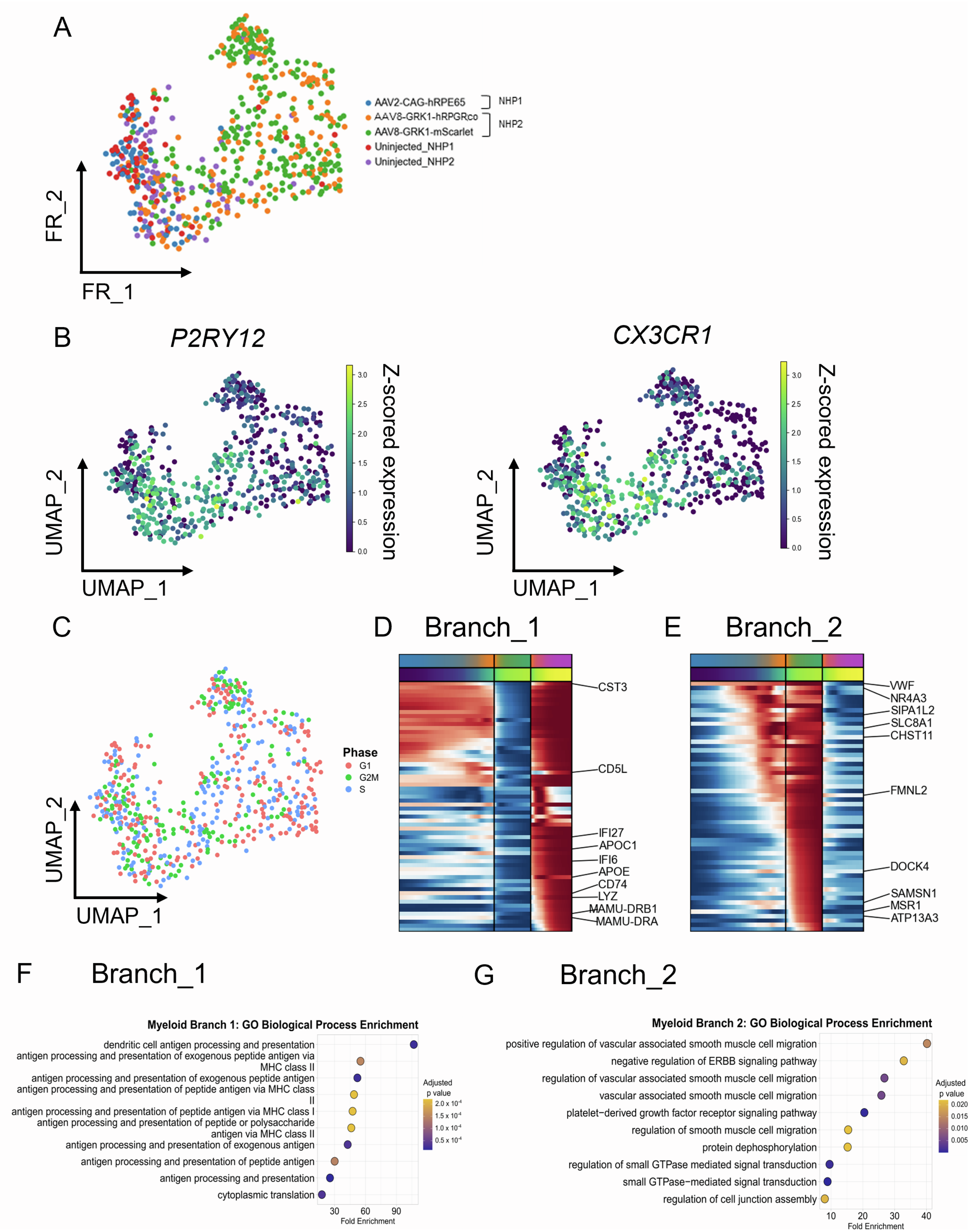
A
AAV2-CAG-hRPE65
AAV8-GRK1-hRPGRco
AAV8-GRK1-mScarlet
Uninjected_NHP1
Uninjected_NHP2
NHP1
NHP2
FR_2
FR_1
B
P2RY12
CX3CR1
Z-scored expression
UMAP_2
UMAP_1
C
Phase
G1
G2M
S
D
Branch_1
CST3
CD5L
IFI27
APOC1
IFI6
APOE
CD74
LYZ
MAMU-DRB1
MAMU-DRA
E
Branch_2
VWF
NR4A3
SIPA1L2
SLC8A1
CHST11
FMNL2
DOCK4
SAMSN1
MSR1
ATP13A3
F
Branch_1
Myeloid Branch 1: GO Biological Process Enrichment
dendritic cell antigen processing and presentation
antigen processing and presentation of exogenous peptide antigen via MHC class II
antigen processing and presentation of exogenous peptide antigen
antigen processing and presentation of peptide antigen via MHC class II
antigen processing and presentation of peptide antigen via MHC class I
antigen processing and presentation of peptide or polysaccharide antigen via MHC class II
antigen processing and presentation of exogenous antigen
antigen processing and presentation of peptide antigen
antigen processing and presentation
cytoplasmic translation
Fold Enrichment
Adjusted p value
G
Branch_2
Myeloid Branch 2: GO Biological Process Enrichment
positive regulation of vascular associated smooth muscle cell migration
negative regulation of ERBB signaling pathway
regulation of vascular associated smooth muscle cell migration
vascular associated smooth muscle cell migration
platelet−derived growth factor receptor signaling pathway
regulation of smooth muscle cell migration
protein dephosphorylation
regulation of small GTPase mediated signal transduction
small GTPase−mediated signal transduction
regulation of cell junction assembly
Fold Enrichment
Adjusted p value

Figure S10. Characterization of the myeloid cell population in AAV-treated NHP retinas.

See Figure 5 B-C. Data based on single-cell RNAseq analysis of retina samples from both NHP1 and NHP2. (**A**) 2-dimensional FR force-directed graph of myeloid cells coloured by treatment conditions. (**B**) Expression of microglia homeostatic markers, *P2RY12* and *CX3CR1*, identified the root for pseudotime analysis. (**C**) Myeloid cells coloured by cell cycle phases. No obvious proliferating cell cluster was detected. (**D**) Heatmap of significant Branch 1-specific gene expression over pseudotime. Detected MHC class I genes were previously observed in Figure S7 A. Detected MHC Class II genes included *Mamu-DRB1* (MHC II beta chain), *Mamu-DRA* (MHC class II alpha chain), *Mamu-DMB* (MHC class II peptide loader), *ENSMMUG00000056183* (ortholog to *Mamu-DQB1*, MHC II DQ beta chain) and *ENSMMUG00000019371* (ortholog to *Mamu-DQA1*, MHC II DQ alpha chain). Detected genes involved in antiviral defence included *RNASE6* (degrades viral RNA), *FGL2* (suppresses immune response, can dampen viral immune response), *BST2* (blocks virus release), *IFI27* (inhibits viral replication), *IFI6* (prevents virus-induced apoptosis), *NPC2* (regulates lipid homeostasis, affecting viral entry and replication) and *STING1* (activates interferon response). Genes commonly associated with pro-inflammatory myeloid cells such as *APOE* (inflammation marker), *APOC1* (modules immune response), *LYZ* (Bacterial defence enzyme), *FCER1G* (activates immune cells) and *C1QB* (complement system component) were also upregulated in Branch 1. (**E**) Heatmap of significant Branch 2-specific gene expression over pseudotime. Upregulated genes were *MAP4K4* (regulates cell migration), *NR4A3* (transcription factor, migration), *MERTK* (phagocytosis and mobility), *MYO1E* (actin-based cell movement), *PTPN1* (tyrosine phosphatase, signalling), *PTPRJ* (regulates cell adhesion and mobility*)* and *CBLB* (ubiquitin ligase, immune signalling). (**F**, **G**) Gene ontology (GO) enrichment analysis on Branch 1 (**F**) and Branch 2 (**G**) specific genes. The majority of Branch_1 detected genes are involved in antigen presentation. Branch_2 genes are involved in cell migration and mobility, GTPase activity and protein dephosphorylation.

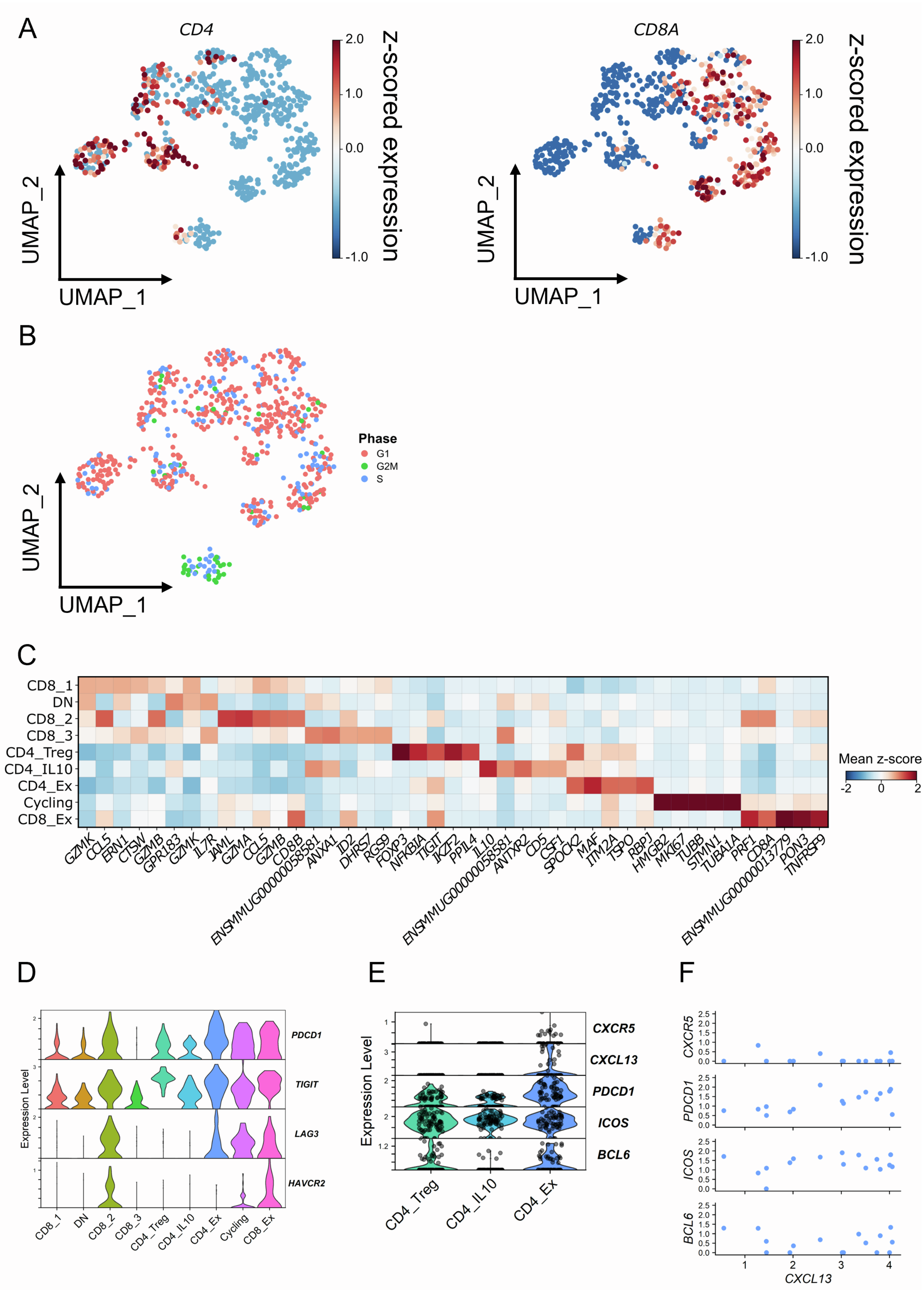
A
CD4
CD8A
Z-scored expression
2.0
1.0
0.0
-1.0
UMAP_2
UMAP_1
B
Phase
G1
G2M
S
C
CD8_1
DN
CD8_2
CD8_3
CD4_Treg
CD4_IL10
CD4_Ex
Cycling
CD8_Ex
Mean z-score
-2
0
2
GZMK CCL5 ERN1 CTSW GZMB GPR183 GZMK IL7R JAML GZMA CCL5 GZMB CD8B ENSMMUG00000058581 ANXA1 ID2 DHRS7 RGS9 FOXP3 NFKBIA TIGIT IKZF2 PPIL4 IL10 ENSMMUG00000058581 ANTXR2 CD5 CSF1 SPOCK2 MAF ITM2A TSPO RBPJ HMGB2 MKI67 TUBB STMN1 TUBA1A PRF1 CD8A ENSMMUG00000013779 PON3 TNFRSF9
D
Expression Level
PDCD1
TIGIT
LAG3
HAVCR2
CD8_1
DN
CD8_2
CD8_3
CD4_Treg
CD4_IL10
CD4_Ex
Cycling
CD8_Ex
E
Expression Level
CXCR5
CXCL13
PDCD1
ICOS
BCL6
CD4_Treg
CD4_IL10
CD4_Ex
F
CXCR5
PDCD1
ICOS
BCL6
CXCL13

Figure S11. Characterization of the T cell population in AAV-treated NHP retinas.

See Figure 5 D-F. Data based on single-cell RNAseq analysis of retina samples from both NHP1 and NHP2. (**A**) CD4 and CD8A marker gene expression enabled differentiation between CD4+ and CD8+ T cells. (**B**) Cells coloured by cell cycle phases, highlighting a significant cluster of proliferating T cells (in S phase). (**C**) Heatmap of major T cell subsets detected based on gene expression profiles. (**D**) Violin plots depicting expression of major immune checkpoint receptor genes (*PDCD1, TIGIT, LAG3 and HAVCR2*). Increased expression was seen in the 'CD8_2' T cell cluster and an exhausted CD8 T cell cluster ('CD8_Ex'). (**E**) Violin plots showing the expression of typical T follicular helper (Tfh) cell marker genes in CD4 clusters. (**F**) Scatter plots showing expression of Tfh marker genes against *CXCL13* in *CXCL13*$^+$ *CD4*$^+$ cells. Co-expression of *PDCD1*, *ICOS* and *BCL6*, but low expression of *CXCR5* might suggest a composition of T peripheral helper cells. (**D**) Violin plots depicting expression of major immune checkpoint receptor genes (*PDCD1, TIGIT, LAG3 and HAVCR2*). Increased expression was seen in the 'CD8_2' T cell cluster and an exhausted CD8 T cell cluster ('CD8_Ex'). (**E**) Violin plots showing the expression of typical T follicular helper (Tfh) cell marker genes in CD4 clusters. (**F**) Scatter plots showing expression of Tfh marker genes against *CXCL13* in *CXCL13*$^+$ *CD4*$^+$ cells. Co-expression of *PDCD1*, *ICOS* and *BCL6* but low expression of *CXCR5* might suggest a composition of T peripheral helper cells.

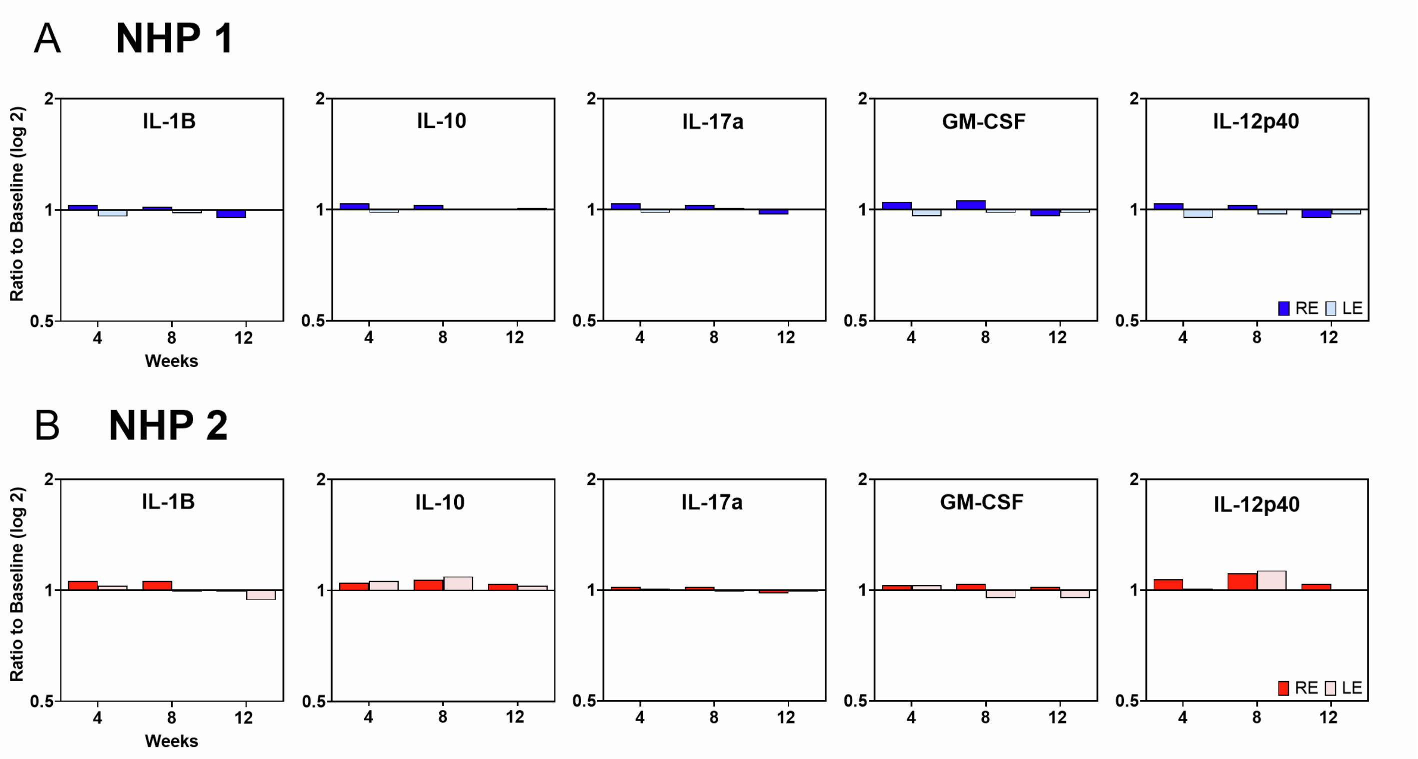

Figure S12. Additional cytokine panels for NHP vitreous samples.

See Figure 6. Additional cytokines assayed as part of the LegendPlex NHP Inflammation Panel applied to the vitreous samples from NHP1 (**A**) and NHP2 (**B**). Ratio scale is in log 2 compared to baseline.

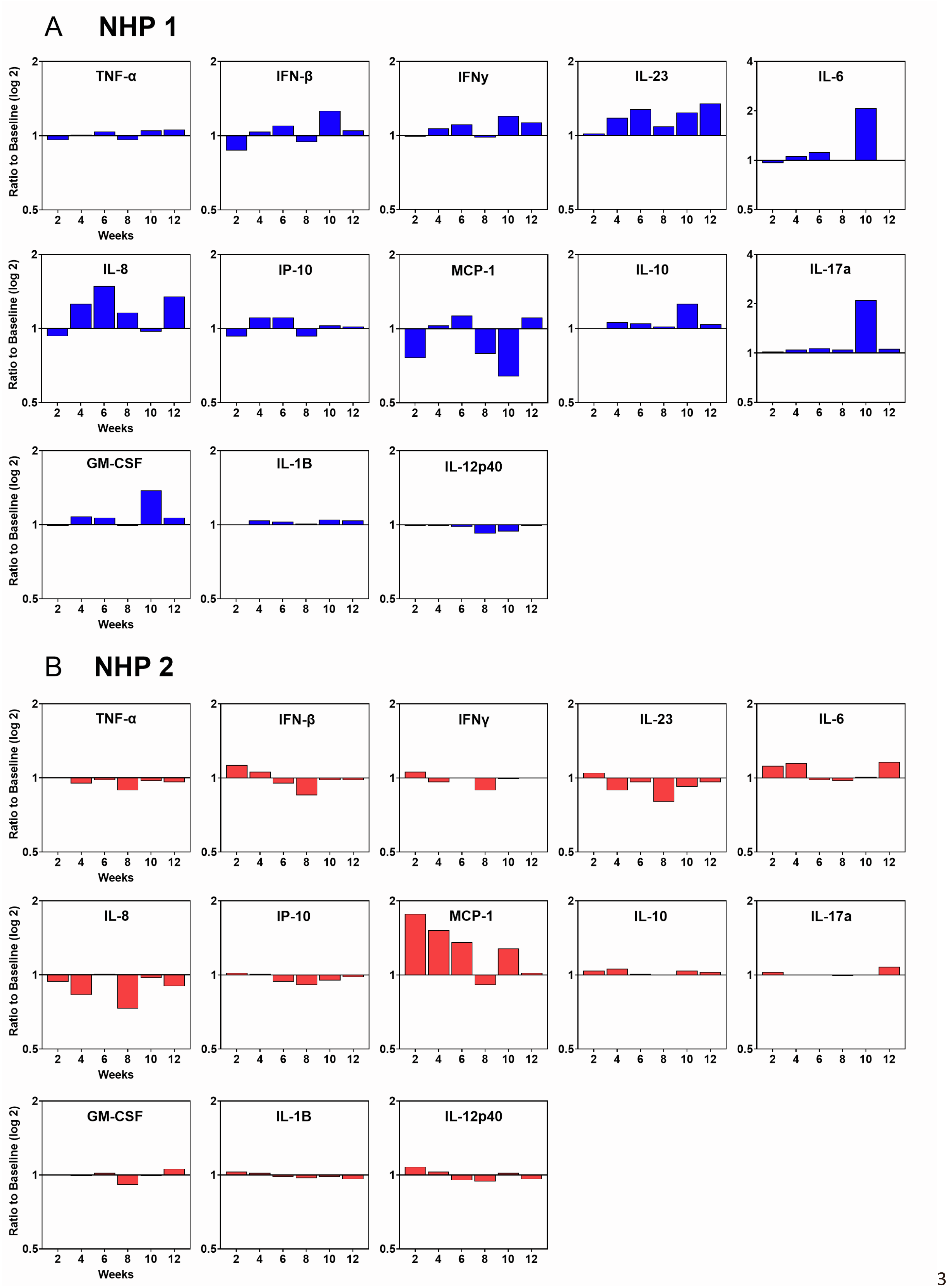
A NHP 1
TNF-α
IFN-β
IFNγ
IL-23
IL-6
IL-8
IP-10
MCP-1
IL-10
IL-17a
GM-CSF
IL-1B
IL-12p40
Ratio to Baseline (log 2)
Weeks
B NHP 2
TNF-α
IFN-β
IFNγ
IL-23
IL-6
IL-8
IP-10
MCP-1
IL-10
IL-17a
GM-CSF
IL-1B
IL-12p40
Ratio to Baseline (log 2)
Weeks

Figure S13. Cytokine profiling of NHP peripheral blood mononuclear cells (PBMCs) revealed no major changes.

Analysis of cytokine expression in the blood samples from NHP1 (**A**) and NHP2 (**B**) following subretinal AAV gene therapy. Ratio scale is in log 2 compared to baseline.

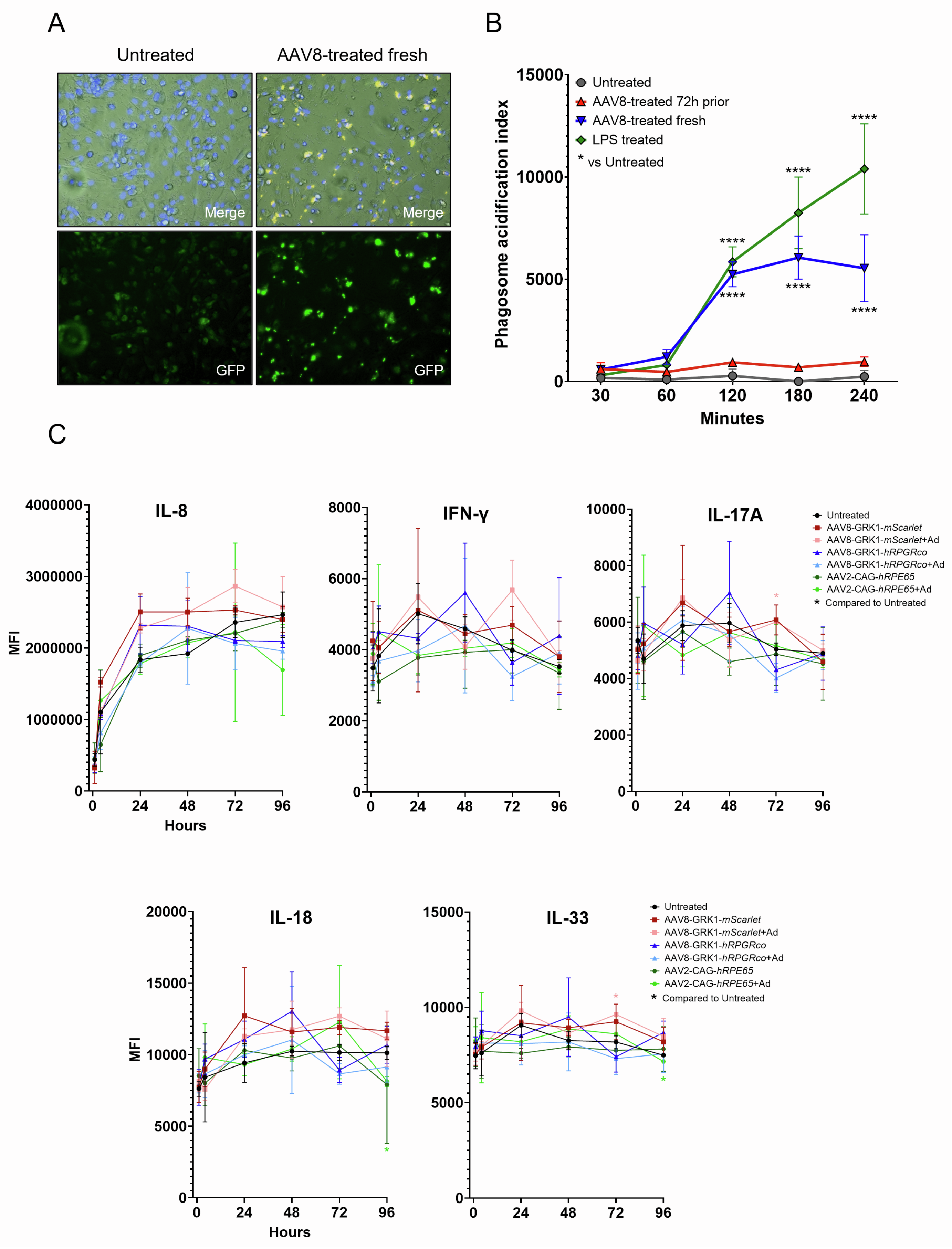
A
Untreated
AAV8-treated fresh
Merge
Merge
GFP
GFP
B
Untreated
AAV8-treated 72h prior
AAV8-treated fresh
LPS treated
* vs Untreated
Phagosome acidification index
Minutes
C
IL-8
IFN-γ
IL-17A
IL-18
IL-33
MFI
Hours
Untreated
AAV8-GRK1-mScarlet
AAV8-GRK1-mScarlet+Ad
AAV8-GRK1-hRPGRco
AAV8-GRK1-hRPGRco+Ad
AAV2-CAG-hRPE65
AAV2-CAG-hRPE65+Ad
* Compared to Untreated

Figure S14. Human iPSC-derived microglia activity and cytokine profile following exposure to AAV vectors.

See Figure 7. (**A**) Representative fluorescence microscopy images showing phagocytosis of pHrodo-labelled zymosan particles by iPSC-microglia exposed to AAV8-CAG-*mScarlet* (AAV8) at 240 min. pHrodo becomes fluorescent upon acidification within endosomes. (**B**) Phagocytic assay comparing (i) naïve microglia exposed to lipopolysaccharide (LPS) (green), (ii) naïve microglia exposed to AAV8-CAG-*mScarlet* (blue), (iii) microglia pre-treated with AAV8 72 hours prior (red), and (iv) untreated naïve microglia (grey). The phagosome acidification index was calculated as the total amount of fluorescence signal above threshold over the number of cells. (**C**) Rest of the cytokine expression panel applied to human iPSC-derived microglia after AAV stimulation. Data are represented as mean ± SD (n=4). Two-way ANOVA test was performed. Ad = adalimumab treatment. * = vs Untreated (UT). * = p>0.01.

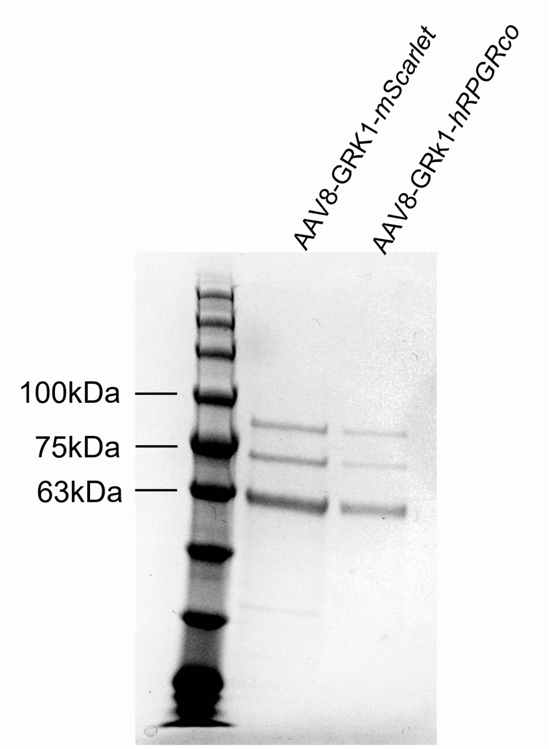

Figure S15. Comparison between purity of AAV8-GRK1-hRPGRco and AAV8-GRK1-mScarlet vector preparations.

Coomassie stained SDS-PAGE of equivalent dose-normalised amounts of both vectors.
