# Supplementary material for: Single-cell and spatial transcriptomic analyses of gene therapy-associated retinal inflammation in non-human primates
Source: Mol Ther Adv. 2026 Mar 30;34(2):201726. doi: 10.1016/j.omta.2026.201726 (PMC13148909; doi:10.1016/j.omta.2026.201726)
Supplement: Document S2. Article plus supplemental information [file mmc2.pdf]

Molecular Therapy
Advances
Original Article

Single-cell and spatial transcriptomic analyses of gene therapy-associated retinal inflammation in non-human primates

Célia Sourd,[1,6] Joel Quinn,[1,6] Molly C. John,[1] Cristina Martinez-Fernandez de la Camara,[1,2] Lakshanie C. Wickramasinghe,[3] Moustafa Attar,[3] Hoda Shamsnajafabadi,[1] Ahmed Salman,[1] Sally A. Cowley,[4] Calliope A. Dendrou,[3] Robert E. MacLaren,[1,2] Jasmina Cehajic-Kapetanovic,[1,2] and Kanmin Xue[1,2,5]

[1]Nuffield Laboratory of Ophthalmology, Nuffield Department of Clinical Neurosciences, University of Oxford, Oxford OX3 9DU, UK; [2]Oxford University Hospitals NHS Foundation Trust, Oxford OX3 9DU, UK; [3]The Kennedy Institute of Rheumatology, University of Oxford, Oxford OX3 7FY, UK; [4]James and Lillian Martin Centre for Stem Cell Research, Sir William Dunn School of Pathology, University of Oxford, Oxford OX1 3RE, UK; [5]Great Ormond Street Hospital for Children NHS Foundation Trust, London WC1N 3BH, UK

Adeno-associated viral (AAV) vectors are rapidly advancing as gene therapies for retinal diseases, but gene therapy-associated uveitis (GTAU) limits their broader application. We assessed the ocular immune response to subretinal AAV gene therapy in two non-human primates (NHPs): NHP1 received bilateral AAV2-CAG-*hRPE65* (voretigene neparvovec) at clinical dose; NHP2 received AAV8-GRK1-*hRPGRco* alongside an analogous *mScarlet* reporter vector in separate blebs. Longitudinal assessments over three months included multimodal imaging, electroretinography, cytokine profiling, followed by immunohistological, single-cell, and spatial transcriptomic analyses of retinal punches. Both therapies were well-tolerated with preservation of retinal structure and function. Single-cell RNA sequencing revealed that the AAV8 vector transduced 80% of cones/rods in treated areas, while AAV2 targeted 30% of RPE/rods. Transgene expression did not correlate with apoptotic markers. At three months, a persistent type 1 cell-mediated response was detected in the retina dominated by myeloid and T cells. Adjunctive intravitreal anti-TNF-α (adalimumab) did not mitigate this chronic anti-viral response. Spatial transcriptomic analysis and immunohistochemistry localized monocytic phagocytes to the subretinal space, consistent with upregulated cytokines (MCP-1/CCL2, IP-10/CXCL10, IL-8/CXCL8, and IL-6), implicating these cells in driving local inflammation. These findings elucidate the mechanism of GTAU and identify potential therapeutic targets to prevent immune-mediated complications in retinal gene therapy.

INTRODUCTION

A large number of adeno-associated viral (AAV) vector-mediated retinal gene therapies are in development for the treatment of inherited retinal degenerations (IRDs) and common retinal disorders such as age-related macular degeneration (AMD).[1] Voretigene neparvovec (Luxturna, Spark Therapeutics Inc., USA), a subretinally administered AAV serotype 2 vector encoding human *RPE65* under the control of a ubiquitous CAG promoter (AAV2-CAG-*hRPE65*), was the first retinal gene therapy to gain FDA approval in 2017 for the treatment of IRD associated with bi-allelic mutations in *RPE65*.[2] More recently, clinical trials of another subretinal gene therapy (cotoretigene toliparvovec, Biogen Inc., USA) for X-linked retinitis pigmentosa (XLRP) demonstrated significant improvements in low-luminance visual acuity and retinal sensitivity.[3,4] This consisted of an AAV8 vector expressing a codon-optimized human retinitis pigmentosa GTPase regulator (*hRPGRco*) transgene under the control of a photoreceptor-specific G protein-coupled receptor kinase 1 (GRK1) promoter. While the pivotal trial for cotoretigene toliparvovec was insufficiently powered to achieve the primary endpoint for clinical approval, a similar vector using an AAV2 capsid variant (AAV2tYF-GRK1-*hRPGRco*, laruparetigene zovaparvovec, Beacon Therapeutics Inc., USA) is currently undergoing randomized, multi-centre phase 3 trial for XLRP (ClinicalTrials.gov: NCT04850118).[5]

Despite relative immune privilege of the eye associated with the blood-retinal barrier, vector dose-related intraocular inflammation, also termed gene therapy-associated uveitis (GTAU), has been frequently observed in clinical trials and post-approval studies.[3,6–12] GTAU could not only present clinically in the form of vitritis, retinitis, cystoid macular edema, or choroiditis but may also manifest subclinically in the form of subretinal deposits seen on optical coherence tomography (OCT).[1] Furthermore, concerns have arisen with the observation of progressive chorioretinal atrophy (CRA) in

Received 31 July 2025; accepted 26 March 2026;
https://doi.org/10.1016/j.omta.2026.201726.

[6]These authors contributed equally

Correspondence: Kanmin Xue, Nuffield Department of Clinical Neurosciences, University of Oxford, John Radcliffe Hospital, Headley Way, Level 6 West Wing, Oxford OX3 9DU, UK.
E-mail: kanmin.xue@eye.ox.ac.uk

© 2026 The Author(s).
Published by Elsevier Inc. on behalf of The American Society of Gene and Cell Therapy.
This is an open access article under the CC BY license (http://creativecommons.org/licenses/by/4.0/).

patients after treatment with voretigene neparvovec, which appears to correlate with AAV dose and may be a result of inflammatory response in the retina.[13,14] Previous mouse study has indicated the local immunological reaction to subretinal AAV gene therapy to consist of a chronic type 1 cell-mediated response with infiltration of myeloid and T cell in the retina.[15] However, the immunological responses in rodents may differ significantly from those in humans, thus primate data is vital to provide more direct understanding of the mechanism of GTAU.

In this study, we sought to gain deep insights into the cellular responses to subretinal AAV gene therapy in non-human primates (NHPs) at single-cell resolution using two clinically important AAV vectors: (1) GMP-grade AAV2-CAG-*hRPE65* (voretigene neparvovec) and (2) engineering-grade AAV8-GRK1-*hRPGRco*. We also assessed the effects of intravitreal anti-TNF-α antibody, adalimumab, as a potential adjunct to prevent intraocular inflammation following gene therapy based on its efficacy in treating non-infectious uveitis.[16–18] Our results demonstrate that subretinal administration of AAV vectors leads to the activation of microglia and infiltration of monocytic phagocytes. Monocytic phagocyte-derived pro-inflammatory cytokines and chemokines appear to drive a chronic type 1 cell-mediated antiviral response characterized by T cells (mainly CD8 effector memory cells) and myeloid cells. Although the magnitude of retinal inflammation varied between different vector products and doses, the nature of the immune cell infiltrate remained consistent. Therefore, our data reveal the key immune ligand-receptor signaling pathways which could be targeted for specific immunomodulation to control GTAU.

RESULTS

Longitudinal assessment of retinal structure and function following subretinal gene therapy in NHPs

In order to simulate the clinical impact of AAV vector-mediated gene therapy and associated immune response, two wild-type NHPs underwent 3-port 23-gauge pars plana vitrectomy and subretinal administration of clinical doses of AAV vectors via 38G subretinal cannula in both eyes, as per current human surgical technique (Figure 1A). The first animal (NHP1) received a total dose of 1.5×10^{11} vector genomes (vg) of clinical grade voretigene neparvovec (Luxturna) (AAV2-CAG-*hRPE65*), which expresses human *RPE65* transgene from a ubiquitous CAG promoter, in two separate subretinal blebs in each eye (Figure 1B). The second animal (NHP2) received subretinal administration of 1.25×10^{11} vg of an engineering grade AAV8 vector (AAV8-GRK1-*hRPGRco*) expressing codon-optimized human *RPGR* transgene under the photoreceptor-specific GRK1 promoter in each eye (Figure 1C). In addition, NHP2 also received 2.5×10^{10} vg of a laboratory grade AAV8 vector (AAV8-GRK1-*mScarlet*) in a separate bleb which is analogous to the therapeutic vector but expresses a fluorescent reporter, mScarlet. This reporter vector (AAV8-GRK1-*mScarlet*) was included to help visualize transgene expression *in vivo* by OCT and fundus autofluorescence (AF) imaging. At the end of surgery, each eye received a subtenon injection of 40 mg of triamcinolone. The left eye of each NHP was also given an intravitreal injection of 1.5 mg adalimumab (an anti-TNF-α biologic), which was subsequently repeated every 4 weeks. We had hypothesized that adjunctive anti-TNF-α may reduce GTAU based on previous clinical report of its efficacy in non-infectious posterior uveitis.[19]

Vision recovery following gene therapy was uneventful except for transient photophobia which resolved spontaneously in NHP2. Follow-up examinations under anesthesia were performed at 2 weekly intervals up to 12 weeks, which included (1) dilated fundal examination with retinal imaging (Heidelberg Spectralis confocal scanning laser ophthalmoscopy [cSLO], OCT, and AF), (2) blood sampling, (3) vitreous biopsy (every 4 weeks), and (4) ERG (every 4 weeks). No clinical signs of media opacity, vitreous or anterior chamber cells were seen during follow-up. Localized hypo-AF was seen around the retinotomy sites and perimeter of blebs by 2 weeks, which became more diffuse over time (Figure S1). In the right eye of NHP1, which was treated with AAV2-CAG-*hRPE65*, marked hypo-AF and RPE mottling developed in the area of bleb 1, which correlated with mild disruption of the ellipsoid zone (EZ) on OCT. Notably, this bleb was relatively small in size as it connected with the larger bleb 2, and consequently received the greatest estimated vector dose per RPE cell (Table 1).[20] In NHP2, the bleb areas treated with laboratory-grade AAV8-GRK1-*mScarlet* (bleb 3) became hyper-AF over the course of 4 weeks, consistent with mScarlet protein expression (Figures 2 and S1). In addition, patchy hypo-AF developed within the *mScarlet* vector-treated areas, which in the left eye coalesced over 8 weeks into a wedge-shaped area of RPE atrophy inferior to the retinotomy site. These changes coincided with the appearance of subretinal hyper-reflective infiltrates on OCT from week 4 to –8 post-treatment (Figure 2). In the area corresponding to bleb 3 in the left eye of NHP2 treated with AAV8-GRK1-*mScarlet*, this change preceded the development of outer retinal and RPE atrophy by 12 weeks. In contrast, a separate area treated by engineering-grade AAV8-GRK1-*hRPGRco* (bleb 4) in the same eye showed only localized changes at the retinotomy site and transient, minor infiltrates at week 4. Both the *hRPE65* and *hRPGRco* vectors were well tolerated, while the *mScarlet* vector was associated with imaging evidence of subretinal inflammation leading to outer retinal atrophy. In the left eye of NHP2, no visible protective effect from adjunctive intravitreal anti-TNF-α treatment was seen against subretinal infiltrates associated with the *mScarlet* vector treatment. The fovea and central retinal thickness (CRT) of all four eyes remained structurally unchanged (Figure S2). At 12 weeks post-gene therapy, all eyes were harvested and processed for immunohistochemistry, spatial transcriptomic, and single-cell transcriptomic analyses.

Light and dark-adapted ERGs were also obtained at baseline and 10 weeks post-gene therapy. Uveitis has previously been associated with prolonged cone b-wave implicit time (correlated to the peak of the 30 Hz flicker response) which could be reversed with treatment.[21,22] In addition, a change in ERG amplitude of >30% would generally be considered clinically significant.[23] In NHP1, no significant change in 30 Hz flicker implicit time was

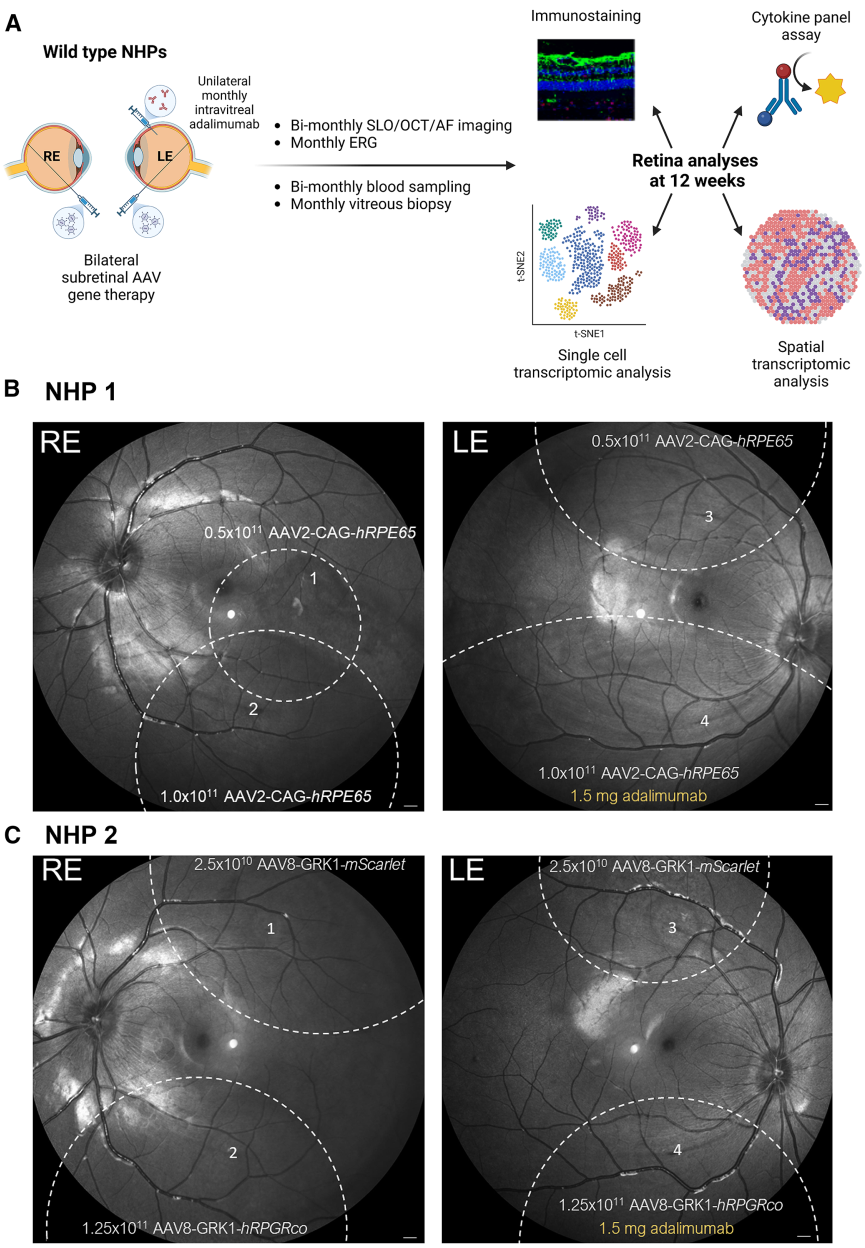

Figure 1. Experimental design

(A) Vitrectomy and subretinal AAV gene therapies were performed to both right (RE) and left (LE) eyes of two wild-type non-human primates (NHPs). The left eyes of both animals also received monthly intravitreal injection of 1.5 mg adalimumab. Retinal structure and function were longitudinally monitored by multimodal imaging and ERG. Vitreous and blood samples were collected at key time points for cytokine analysis. Retinas were harvested at 12 weeks for immunohistochemistry, single-cell RNA-sequencing, and spatial transcriptomic analysis. (B) NHP1 received a total dose of 1.5×10^{11} vg (vector genome) of Luxturna (AAV2-CAG-*hRPE65*) per eye via two subretinal blebs (denoted by dashed lines). (C) NHP2 received 1.25×10^{11} vg of AAV8-GRK1-*hRPGRco* and 2.5×10^{10} vg of an equivalent reporter vector AAV8-GRK1-*mScarlet* in two separate blebs (dashed lines). Note that all retinal images are vertically inverted (top of the image representing inferior retina) due to the imaging device approaching the supine animal from the top. Scale bars, 500 μm.

seen (Figure S3). ERG amplitudes were generally unchanged, except for an increase in scotopic b-wave at DA 0.01 cd·s/m^2 in both eyes (from mean 124 to 208 μV in the right eye and 124 to 178 μV in the left eye). In NHP2, no significant change in 30 Hz flicker implicit time was seen (Figure S4). However, reduction in cone response was observed, particularly in the right eye: e.g., light-adapted (LA) 30 Hz flicker amplitude decreased by 60% (from 113 to 45.3 μV) in the right eye versus 25% (from 120 to 90.3 μV) in the left eye; LA b-wave amplitude decreased by 59% (from 118 to 48.7 μV) in the right eye versus 23% (from 121 to 92.6 μV) in the left eye.

AAV-mediated transgene expression in the retina at single-cell resolution

To evaluate the efficiency of AAV-mediated gene therapy *in vivo*, transgene expression was analyzed in both NHP1 and NHP2. Full thickness retinal punches were obtained from areas within and outside the subretinal blebs. Conventional immunohistochemistry was performed on retinal sections (Figure 3A and 3D). Due to high levels of amino acid sequence homology between macaque and human RPE65 (99%) and RPGR (94%), both the native and transgene-derived proteins were detected with the antibodies. Of note, protein expression appeared higher within the treated blebs than in untreated areas, consistent with vector-mediated transgene expression. Both the native and transgene-derived RPE65 protein appeared to localize to the RPE layer. However, RPGR staining, which is typically localized to the photoreceptor connecting cilia as seen in untreated regions, extended into other parts of the photoreceptor cells, including the inner segments and cell bodies (Figure 3D). While such extension of RPGR expression into photoreceptor cell bodies has previously been attributed to fixation artifacts,[24] it may be a result of AAV-mediated overexpression leading to protein diffusion beyond its normal subcellular localization.

Using spatial transcriptomic analysis (Visium Spatial v.1 3′ Gene Expression, 10× Genomics), we correlated H&E-stained retinal sections with the transcriptome at 55 μm spatial resolution. As this version of Visium is based on polyA capture, the platform enabled robust differentiation between native macaque mRNA transcripts

Table 1. Summary of subretinal dosing of AAV vectors in two non-human primates

NHP	R/L eye	Subretinal bleb no.	AAV vector	Dose injected (vg)	Bleb area (mm^2)	Estimated dose per RPE cell
1	RE	1	AAV2-CAG-*hRPE65*	0.5×10^{11}	3.4	3,676,471
		2	AAV2-CAG-*hRPE65*	1.0×10^{11}	28.3	883,392
	LE	3	AAV2-CAG-*hRPE65*	0.5×10^{11}	34.9	358,166
		4	AAV2-CAG-*hRPE65*	1.0×10^{11}	201.1	124,316
2	RE	1	AAV8-GRK1-*mScarlet*	0.25×10^{11}	48.6	128,601
		2	AAV8-GRK1-*hRPGRco*	1.25×10^{11}	38.5	811,688
	LE	3	AAV8-GRK1-*mScarlet*	0.25×10^{11}	22.3	280,269
		4	AAV8-GRK1-*hRPGRco*	1.25×10^{11}	38.5	811,688

Vector dose (vector genome) per RPE cell for each bleb (numbered in accordance with Figures 1B and 1C) has been estimated based on the actual volume of vector injected, the bleb size, and an estimated RPE cell density of 4,000 cells/mm^2.

and AAV-derived human transgene expression. Human *RPE65* transgene expression could be seen to localize to the RPE layer and expression level appeared to be lower than native macaque *RPE65* (Figure 3B). In contrast, human codon-optimized *RPGR* transgene expression was seen at higher level than native *RPGR* in the outer retina (Figure 3E), consistent with the protein overexpression suggested by immunostaining.

Transgene expression was further characterized by single-cell RNA sequencing (scRNA-seq) of dissociated retina punches taken from within treated bleb areas and untreated control areas of each eye. A total of 89,020 cells were captured following quality control. Clusters corresponding to retinal cell types were identified using marker genes, including amacrine cells (AC; *CALB2* and *SLC6A9*), bipolar cells (BPC; *TRPM1* and *GRIK1*), cone photoreceptors (*ARR3*, *OPN1SW*, and *OPN1LW*), horizontal cells (HC; *ONECUT1*), immune cells (IC; PTPRC), Müller glia (MG; *SLC1A3*, *GLUL*, *VIM*, and *CRABP1*), RPE cells (*RDH5*, *RLBP1*, and *RPE65*), and rod photoreceptors (*NRL*, *NR2E3*, *PDE6B*, and *AIF1*), thereby enabling analysis of transgene expression at the level of individual cell types (Figures S5A and S5B).[25] Retinal cell populations were comparable between treated and untreated areas in both NHPs (Figure S5C). In the NHP1 retina treated with AAV2-CAG-*hRPE65*, *hRPE65* transgene expression was detected at high level in approximately 30% of captured RPE cells, at moderate level in 40% of rods, and relatively low level in 15% of cones (Figure 3C). Bipolar cells and Müller glia showed none to minimally detectable levels of transgene expression. This distribution appears to reflect the selective tropism of AAV2 for RPE and photoreceptors and efficiency of the ubiquitous CAG promoter among these cell types. In addition, *hRPE65* transgene expression was nearly undetectable outside the subretinal bleb area, indicating minimal lateral spread of the viral vector across the NHP retina. In the NHP2 retina treated with AAV8-GRK1-*hRPGRco*, *hRPGR* transgene expression was detected in 80% of cones, 75% of rods, and 40% of RPE cells within the treated areas (Figure 3F). Low level of off-target transgene expression was also detected in approximately 50% of Müller glia, whereas no significant expression was detected in bipolar cells. Similar distribution and proportion of cellular transduction was mirrored in the areas treated with AAV8-GRK1-*mScarlet* reporter vector, which represents the tropism profile of AAV8. Taken together, these results indicate highly efficient *in vivo* transduction of primate photoreceptors by AAV8 and transgene expression from the photoreceptor-specific GRK1 promoter.

It has been hypothesized that transgene overexpression might cause metabolic stress and cell toxicity. We investigated this by looking for any correlation between the levels of expression of the transgene and apoptotic marker genes at single-cell level using the Apoptosis MSigDB Hallmark gene set among the rod, cone, Müller glia, and RPE populations. The expression levels of apoptotic marker genes were found to be low overall and comparable between the treated and untreated areas in both NHPs (Figure S6).

Immune cell population identified by spatial transcriptomic mapping

The retina is considered a relatively immune privileged site due to the presence of the blood-retinal barriers, with microglia being the main resident immune cell population under physiological conditions. Our previous rodent study indicates that subretinal AAV gene therapy induces a delayed cell-mediated immune response in the retina, which is detectable by 2 weeks, peaks around 3–4 weeks, and persists significantly beyond.[15] The timing of this immune response appears to correlate with clinical presentation of GTAU, which is an important limiting factor on treatment safety and efficacy.[11] The extent of the immune response is known to correlate with vector dose but may also be affected by the type and preparation of viral vector.[1,26]

We analyzed retina sections from NHP1 and NHP2 at 3 months post-gene therapy for immune cell populations. In NHP1, immunostaining for the pan-immune cell marker CD45 detected minimal immune cell infiltrate histologically in the *voretigene neparvovec*-treated bleb areas compared with untreated areas (Figure S7A). Staining for GFAP, a marker of gliotic response by Müller cells, did not reveal significant difference between treated and untreated retinas. In contrast, retina sections from NHP2 showed greater CD45 and GFAP staining within vector-treated areas, indicating

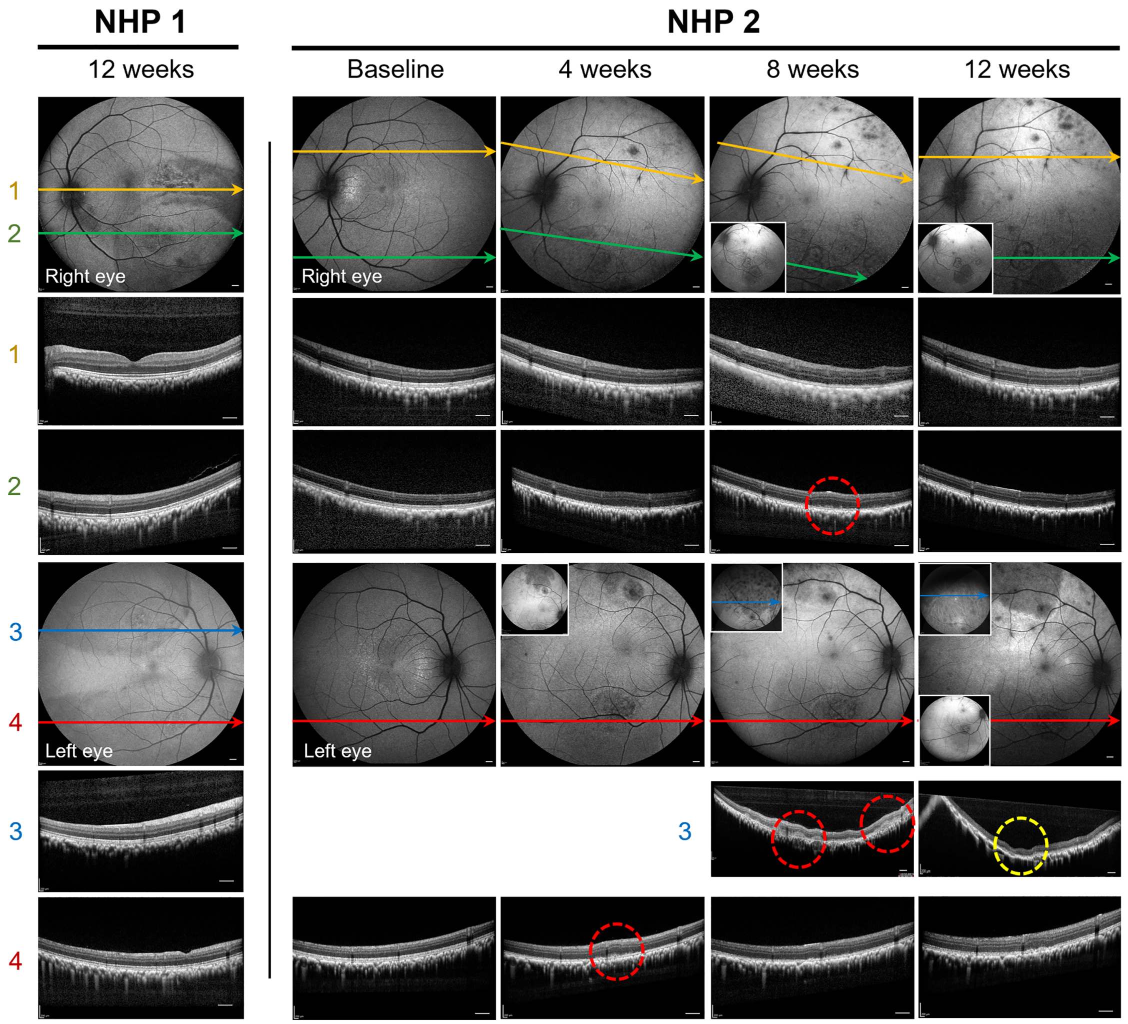

Figure 2. Mild perturbation of retinal structure following subretinal AAV gene therapy in non-human primates

Longitudinal multimodal retinal imaging following subretinal injection of AAV vectors over 12 weeks of both eyes of NHP1 and NHP2. The color lines represent the locations of OCT sections from inside the treated subretinal blebs. Note that all retinal images are vertically inverted (top of the image representing inferior retina). Red-dashed circles indicate subretinal infiltrates seen from 4 to 8 weeks. In the left eye inferior arcade of NHP2 (within an area treated by AAV8-GRK1-mScarlet), this was followed by RPE/outer retinal atrophy associated with hypo-autofluorescence (yellow-dashed circle). Note that the surrounded area treated by the mScarlet vector also demonstrated increased background hyper-autofluorescence, indicative of fluorescent reporter expression. Scale bars, 500 μm.

immune cell infiltration and gliosis (Figure S7C). While immunostaining could detect gross changes in retinal histology, it provides insufficient sensitivity to probe subclinical immune responses. To overcome this limitation, we applied the spatial transcriptomic approach to identify specific immune cell populations through their distinct marker gene expression profiles, e.g., T cells (*CD8A*, *CD3E*, *CD4*, and *FOXP3*), B cells (*CD19*, *CD79A*, and *MS4A1*), and natural killer (NK) cells (*NKG7* and *NCR1*). No significant differences were observed when comparing B cell and NK cell populations between within and outside the treated bleb from NHP1 (0.40% of positive "spots" in the treated blebs of Figure S7B), but NHP2 retina sections displayed small B cell (2.24%) and NK cell (1.28%) clusters in the choroid and the RPE (Figure S7D). In contrast, T cell clusters appeared more abundant inside the AAV-treated blebs of both NHP1 and NHP2 (2.81% and 7.69%, respectively).

Furthermore, we looked for evidence of microglia, macrophages, and myeloid cell activity within the retina (Figure 4). Visium spatial transcriptomic mapping shows clusters of monocytic phagocytes (expressing marker genes *P2RY12* and *HEXB*). NHP1 and NHP2 tissue sections displayed similar amounts of monocytic phagocytes in the treated blebs (8.05% and 9.62% of cell clusters). However, the most striking observation was that while the monocytic phagocytes were distributed across different retinal layers in the untreated areas (thus likely to represent resident microglia), they became concentrated in the subretinal space in the treated retinal sections of both NHPs

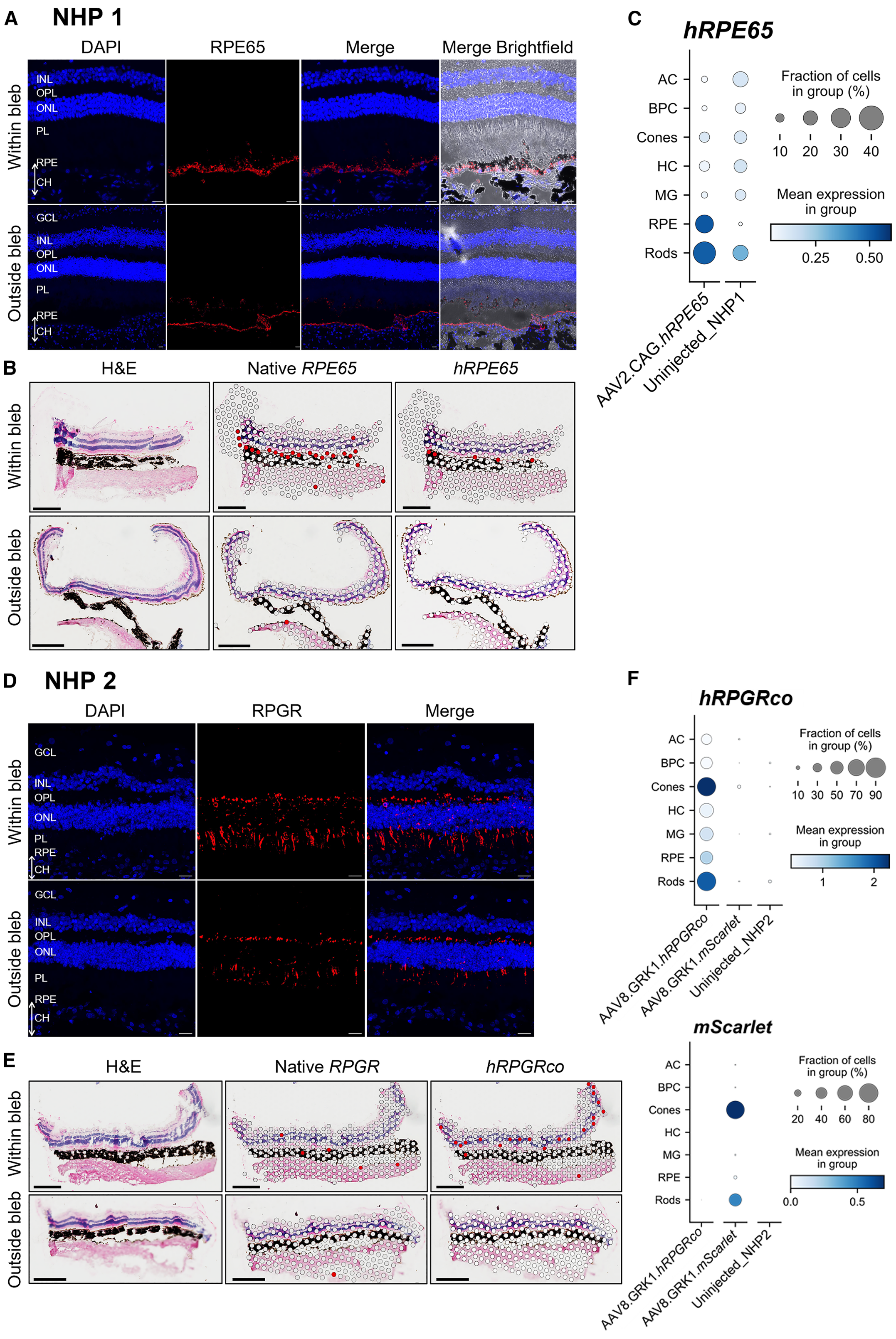

(legend on next page)

(Figures 4A and 4C). This is potentially indicative of microglia activation or myeloid cell infiltration to the subretinal space in response to subretinal AAV administration. To corroborate the spatial transcriptomic findings, immunostaining with IBA1 antibody was performed on adjacent retinal sections from the same tissue blocks. Staining was limited in NHP1. In NHP2, amoeboid IBA1$^+$ cells were relatively enriched in the sclera and subretinal space of the treated bleb compared with untreated regions, whereas the inner and outer plexiform layers showed very little IBA1 staining (Figures 4B and 4D).

Single-cell characterization of the nature of GTAU

To characterize the nature of retinal inflammation and outer retinal atrophy seen in the AAV8-GRK1-*mScarlet* vector treated region in NHP2, we compared the single-cell transcriptome of retina from this area with an untreated control area from the same eye (Figure S8). Differentially upregulated genes were predominantly identified in the rods within the vector-treated area, including *B2M* (MHC I light chain), *ENSMMUG00000054038* (macaque MHC I antigen), *ENSMMUG00000064120* (ortholog to *CD1D*, MHC-like lipid antigen presenter), *ENSMMUG00000050829* (MHC I pathway regulator), *ENSMMUG00000052293* (ortholog to *KIAA1109*, MHC I complex assembly), and *ENSMMUG00000058325* (MHC I antigen), which indicate upregulated MHC class I antigen presentation (Figure S8A). A similar set of MHC class I genes were also upregulated in cones. Moreover, Gene Ontology (GO) enrichment analysis revealed upregulation of gene sets in vector-treated rods that are linked to immune response against viral or cytokine stimuli (Figure S8B). Additionally, of the 58 genes differentially expressed by rods in the AAV8-GRK1-*hRPGRco* bleb, 54 overlapped with the differentially expressed genes in the AAV8-GRK1-*mScarlet* bleb (Figure S8C).

Next, we analyzed the immune cell clusters (*PTPRC*$^+$, 1,397 cells) from the whole scRNA-seq dataset, comprising both NHP1 and NHP2. Myeloid cells (*ITGAM*, 560 cells), T/NK cells (*CD3D*, 642 cells), and B cells (*MS4A1*, 68 cells) made up the bulk of the retinal immune cell infiltrate (Figures 5A and S9). At these depths, populations at $\geq$2%–3% frequency are expected to be captured with at least 10 cells per compartment ($\geq$95% probability), enabling robust characterization of dominant immune cell populations, while rarer states are described qualitatively. Interestingly, a cluster of endothelial/smooth muscle cells (*RGS5*, 118 cells) was also present among the annotated immune cell cluster. Myeloid cells were present in significant numbers in all retinal punch areas, including those that were untreated (Figures S9B and S9C). As expected, the AAV8-GRK1-*mScarlet* and AAV8-GRK1-*hRPGRco* vector-treated areas contributed high proportions of the T cell and B cell clusters, which is indicative of localized adaptive immune response to AAV treatment (Figure S9C).

To further characterize microglia/myeloid cell activation in response to AAV, we performed trajectory inference and pseudotime analysis on the *ITGAM*$^+$ subcluster (Figure 5B and 5C, and S10). The force-directed graph of myeloid cells showed a clear distinction between those present in all retinal areas and expressing homeostasis marker genes (*CX3CR1* and *P2RY12* high), and those present in the AAV8-GRK1-*mScarlet* and AAV8-GRK1-*hRPGRco* vector-treated areas (Figures S10A and S10B). Trajectory analysis suggested a bifurcation in myeloid cell trajectory over pseudotime whose termini we labeled as branch 1 and branch 2 (Figure 5C). We did not find any of the clusters or either termini to be associated with a particular cell-cycle phase, suggesting that cell division did not significantly influence the trajectory (Figure S10C). We then performed statistical analysis of branch-specific genes and subsequent Gene Ontology (GO) enrichment analysis to identify branch-specific transcriptional programmes. We found that branch 1 expressed a number of enriched GO gene sets largely associated with antigen presentation via both MHC class I and MHC class II, as well as viral response, lipid transport, and other immune-related processes (Figure S10D–G).

We next used a similar approach to identify subpopulations of T cells and found 4 clusters corresponding to CD8 T cells and 3 clusters corresponding to CD4 T cells (Figures 5D and S11A). In addition, we found an apparent CD4$^-$ CD8$^-$ double-negative (“DN”) cluster, as well as a proliferating (“cycling”) cluster with high S phase and G2M phase scores (Figure S11B). To broadly understand the T cell states in AAV-treated retinas, we compared our data with ProjecTILs, a well characterized and labeled T cell reference dataset, for classifying T cell subpopulations across species in the context of chronic viral infections (Figure 5E).[27] The majority of CD8 T cells within our dataset (50%) projected onto the “CD8 Effector Memory” cluster in ProjecTILs, with a smaller subset projecting onto the “CD8 Exhausted” cluster. A small number of cells also projected onto the “CD8 Naive like” cluster. The CD4 T cells mostly projected onto the Th1 and Tfh clusters, with a small number of Tregs (Figure 5F). The CD8 T cells in AAV-treated retina showed high level expression of cytotoxic genes (*GZMA*, *GZMB*, *GZMK* [granzyme A, B, and K], and *PRF1* [perforin]) with variation across clusters suggesting different activation states (Figure S11C). One CD8 cluster also exhibited signs of exhaustion, with increased expression of *PRF1*, *CD8A* (cytotoxic T cell marker), and *TNFRSF9* (co-stimulatory receptor), as well as high expression of immune checkpoints

Figure 3. Robust expression of human RPE65 and RPGR transgenes in AAV-treated NHP retinas

(A and D) Immunostaining of retina sections from AAV2-CAG-*hRPE65* (NHP1) and AAV8-GRK1-*hRPGRco* (NHP2) treated areas detect both native NHP and transgene-derived human RPE65 and RPGR proteins, respectively. Control sections were taken from outside the treated blebs. GCL, ganglion cell layer; INL, inner nuclear layer; OPL, outer plexiform layer; ONL, outer nuclear layer; PL, photoreceptor layer; RPE, retinal pigmented epithelium; CH, choroid. Scale bars, 20 μm. (B and E) Spatial transcriptomic maps of native versus human RPE65 (NHP1) and RPGR (NHP2) gene expression within retina sections. Red spots represent the locations of cell clusters expressing the gene of interest overlayed on the H&E-staining image. Scale bars, 0.5 mm. (C and F) scRNA-seq reveals the levels of transgene expression by each retinal cell type. Circle size represents the proportion of cells that express the transgene within each population, while color intensity represents the mean expression level per cell. AC, amacrine cells; BPC, bipolar cells; HC, horizontal cells; MG, Müller glia.

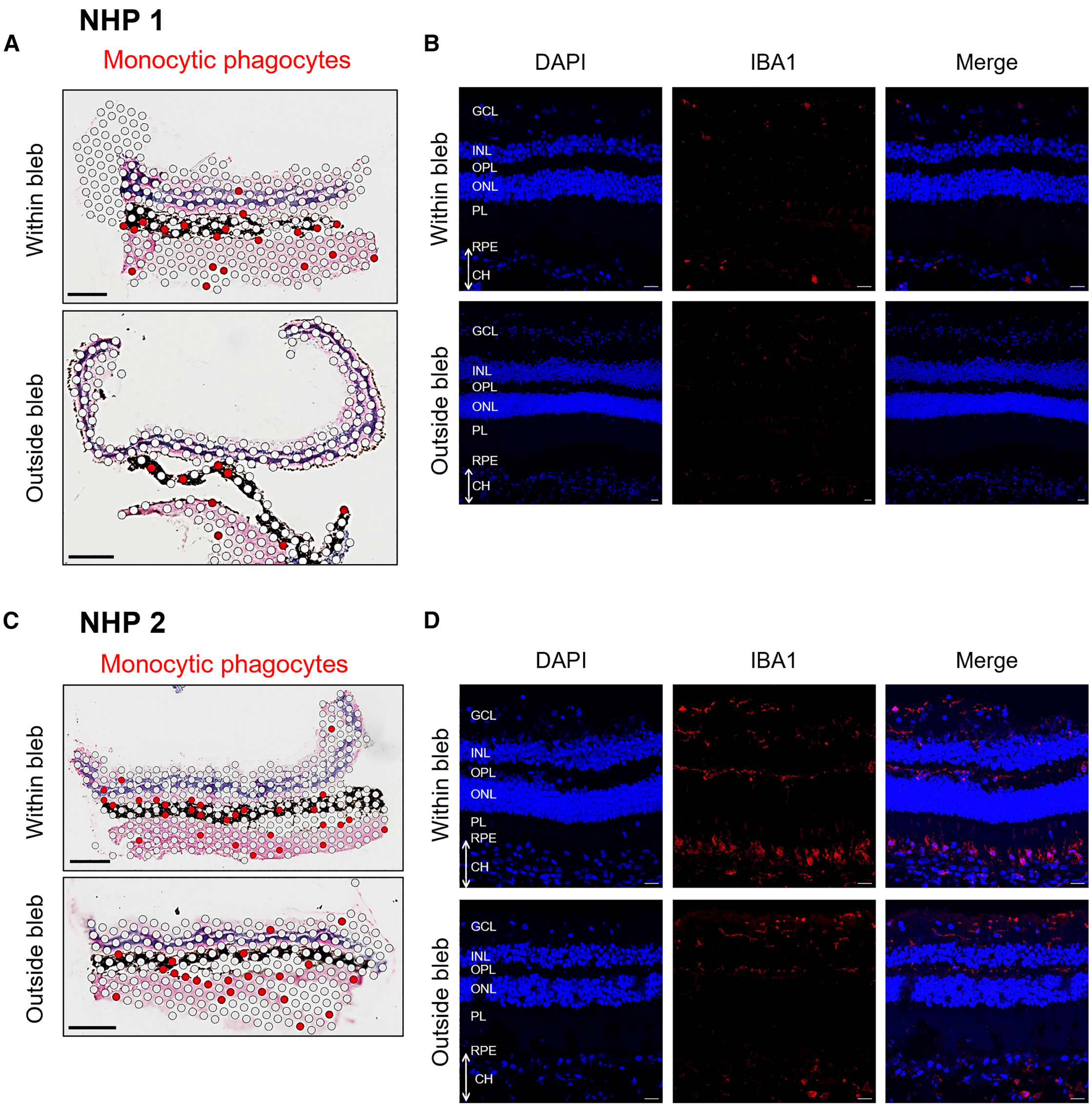

Figure 4. Distribution of mononuclear phagocytes after AAV gene therapy in NHPs

(A and C) Spatial transcriptomic mapping of monocytic phagocytes (including microglia) over treated versus untreated retinal sections from NHP1 (A) and NHP2 (C). Red spots represent cells expressing the marker genes P2RY12 and *HEXB*. Scale bars, 0.5 mm. (B and D) Immunostaining for IBA1 demonstrates amoeboid-shaped monocytic phagocytes, consistent with activation. Scale bars, 20 μm.

PDCD1 (PD-1), *TIGIT*, *LAG3*, and *HAVCR2* (TIM-3) (Figure S11D). Among the CD4 T cells, we identified a Treg subset (*FOXP3*, *TIGIT*, and *IKZF2*) and an *IL-10*$^{+}$ subset, possibly regulating AAV-induced inflammation. While *CXCR5* (indicative of T follicular helper cells) was low, some exhausted CD4 cells co-expressed *CXCL13, PDCD1* and *ICOS*, suggesting a small CXCR5lo T peripheral helper-like population (Figure S11D–F).

Together, these findings suggest that subretinal AAV gene therapy in primates triggers a primarily type 1 cell-mediated response. By 3 months post-treatment, retinal CD8 effector memory T cells persist, alongside exhausted and naive-like subsets. The concurrent presence of Th1 and Tfh-like CD4 populations indicates an antigen-experienced T cell milieu, potentially driving chronic retinal inflammation.

Vitreous and retinal cytokine profile following subretinal gene therapy

Vitreous samples taken from each eye at baseline, 4, 8, and 12 weeks after subretinal gene therapy were analyzed for inflammatory cytokines using the LEGENDplex NHP Inflammation Panel (Figures 6A and 6B). This detected significant elevation of interferon gamma-induced protein-10 (IP-10 or CXCL10) in both NHPs and monocyte

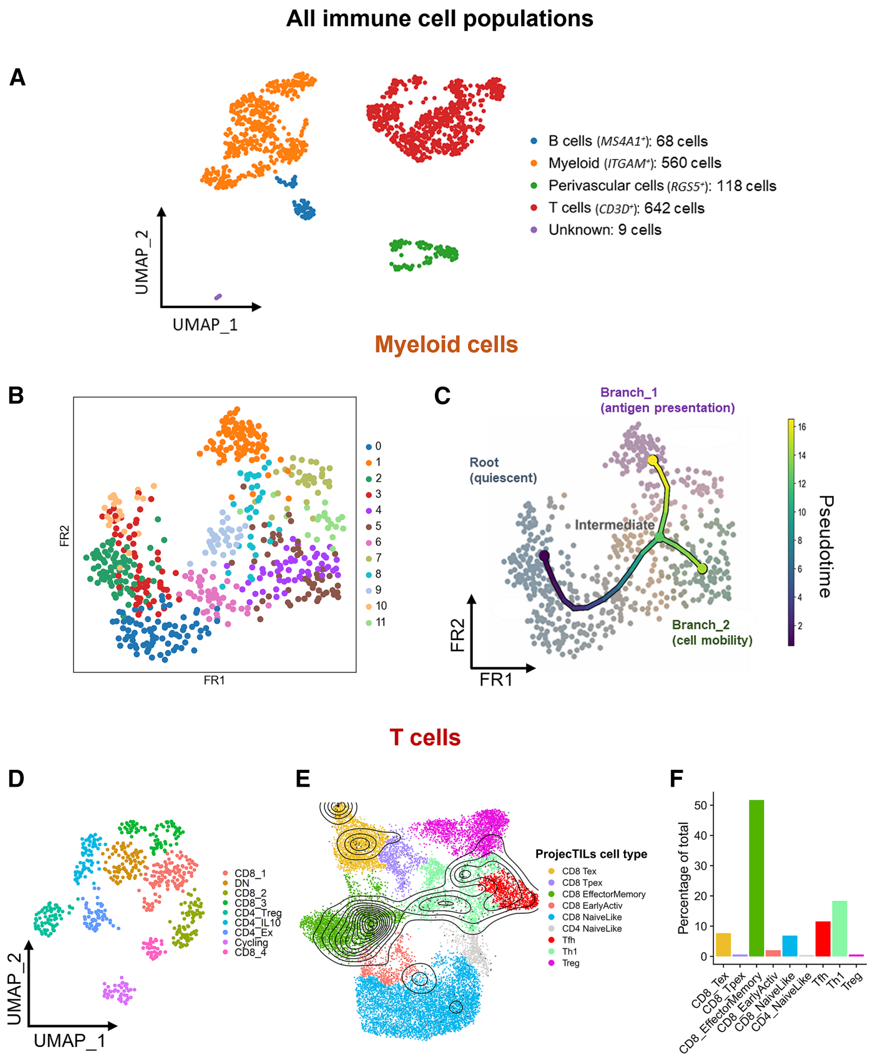

Figure 5. Single-cell transcriptomic analysis reveals a type 1 cell-mediated response in the retina 12 weeks after subretinal AAV gene therapy in non-human primates (NHPs)

(A) Identification of immune population cells in the PTPRC$^+$ cluster. (B) Two-dimensional Fruchterman-Reingold (FR) force-directed graph of myeloid cells colored by unbiased Leiden cluster prior to trajectory inference analysis. (C) Pseudotime analysis of myeloid cells revealed one root (quiescent cells) and two branches—branch 1 (antigen presentation) and branch 2 (cell mobility). Gene ontology enrichment analysis (Figure S9) identified the main biological processes linked with these branches. (D) T cell (CD3D$^+$) subclusters show presence of various CD4 and CD8 subsets, as well as a proliferating ("cycling") cluster with high expression of cell-cycle genes. CD8$^+$ subclusters labeled 1–4, DN, double-negative (*CD4*$^-$ *CD8A*$^-$). (E) Projection of our T cell dataset (black contour plot) onto the ProjecTILs reference dataset (colored UMAP). (F) Percentage of T cells projected onto each ProjecTILs cluster showing a prominent CD8 effector memory cell cluster.

chemoattractant protein-1 (MCP-1 or CCL2) in NHP2. Both IP-10 and MCP-1 act as chemoattractants for monocytes/macrophages, T cells, and NK cells, thus may contribute to persistent intraocular inflammation. All other cytokines in the NHP Inflammation Panel remained at minimal detectable levels at all time points, including TNF-α, IFN-β, IFN-γ, IL-23, IL-6, IL-8, IL-1β, IL-10, IL-17a, IL-12p40, and granulocyte-macrophage colony-stimulating factor (GM-CSF) (Figure S12). The notable absence of significant TNF-α response to AAV gene therapy may account for the lack of efficacy of adalimumab in the prevention of chronic GTAU. In addition, cytokine profiling of blood samples taken across the same time points did not show any significant change from baseline, indicating little systemic inflammatory response to subretinal AAV gene therapy (Figure S13).

To identify which cells are responsible for the production of proinflammatory cytokines in GTAU, we further scrutinized the single-cell dataset. This revealed the myeloid cells in the retina as the dominant source of *MCP-1* (*CCL2*) expression as well as *IL-8* (*or CXCL8*, a neutrophil chemoattractant), *CCL5* (a chemokine for T cells and macrophages), and *TNF-α* (Figures 6C and 6D). In contrast, the Müller glia and RPE cells, which may adopt active immune roles during retinal inflammation, did not demonstrate significant cytokine expression.

Human-iPSC-derived microglia are rapidly activated by AAV *in vitro*

To validate whether microglia could be activated by AAV vectors, we first conducted *in vitro* phagocytosis using human-induced pluripotent stem cell (iPSC)-derived microglia (Figure S14A and S14B). Phagocytic function was compared between (1) naive microglia exposed to lipopolysaccharide (LPS)—positive control, (2) naive microglia exposed to the AAV8-CAG-*mScarlet* vector (labeled as "AAV8"), (3) microglia pre-treated with "AAV8" 72 h prior, and (4) untreated naive microglia—negative control. This showed rapid increases in phagocytosis in both LPS and AAV8-treated microglia from 60 min onward, whereas previous exposure to AAV8 72 h prior did not lead to persistent increase in phagocytic activity. Furthermore, we conducted cytokine profiling of the iPSC-microglia treated with the AAV vectors used for our *in vivo* study: AAV2-CAG-*hRPE65* (in green), AAV8-GRK1-*hRPGRco* (in blue), and AAV8-GRK1-*mScarlet* (in red) (Figure 7 and S14C). In some AAV-treated microglia, adalimumab (+Ad) was also added to the supernatant. Overall, iPSC-derived microglia treated with the *hRPE65* or *hRPGRco* vectors did not display significant increase in cytokine expression. On the other hand, microglia stimulated with the *mScarlet* vector exhibited increased production of TNF-α, IL-6, IL-10, IP-10, and IL-23 from 1 to 96 h. Adalimumab

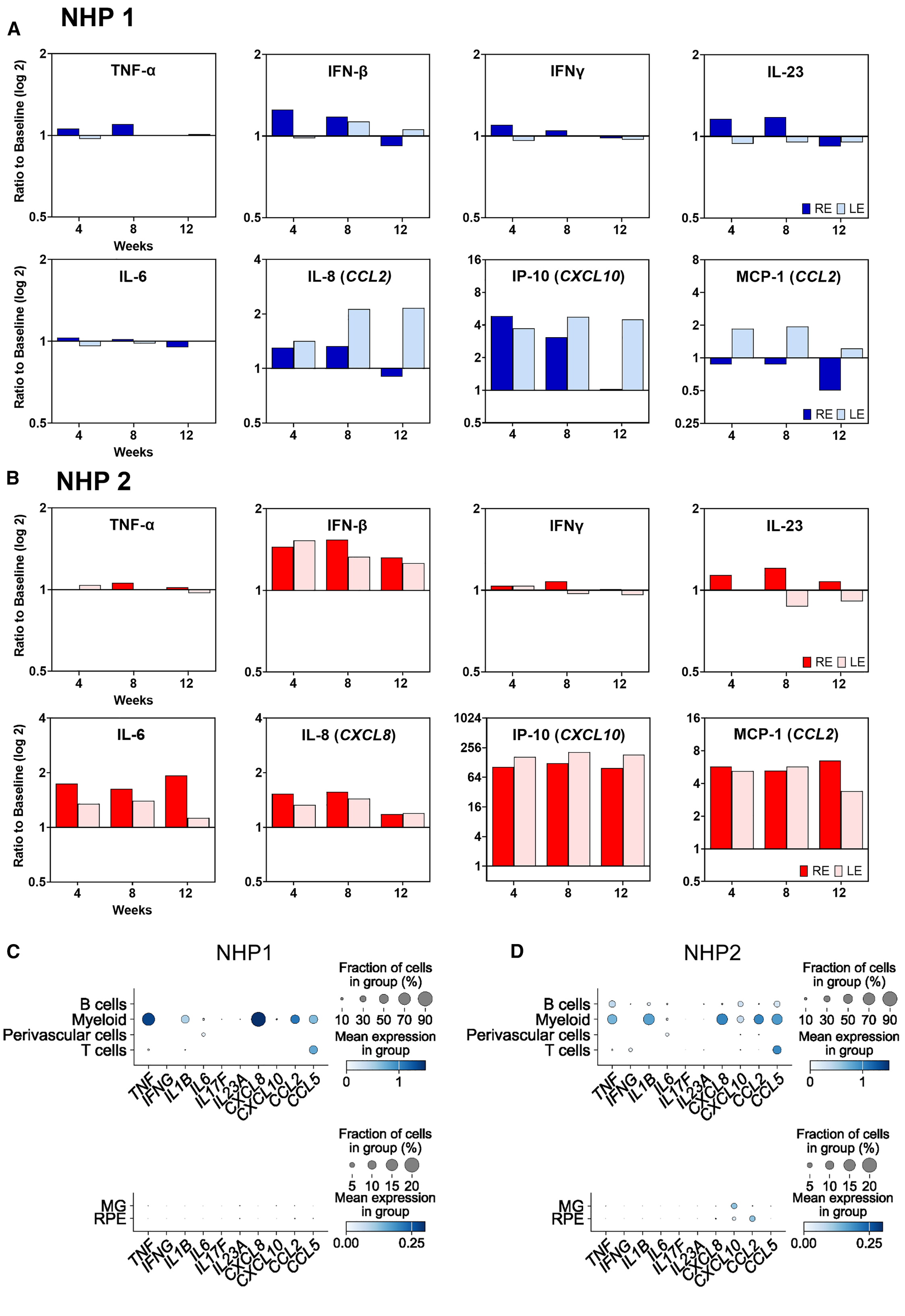

(legend on next page)

treatment mitigated the TNF-α spike induced by this vector in the first 24 h but had little to no significant effect on the other cytokines, thus indicating a selective but limited modulatory effect on vector-induced microglia activation. Interestingly, upregulation of MCP-1 was not detected up to 96 h after AAV treatment. Of note, untreated iPSC-microglia showed a similar increase in MCP-1 production as those treated with AAV vectors, which would suggest this to be a non-specific response potentially to mechanical disturbance. Taken together, the results suggest that microglia possess pattern recognition receptors that can detect the presence of antigens within AAV vector preparation which induce a rapid innate immune response that might contribute to the recruitment of adaptive immunity.

DISCUSSION

In this study, we simulated subretinal gene therapy in primates using human therapeutic AAV vector doses and surgical techniques and conducted deep assessment of the resulting retinal cellular responses at single-cell resolution. Using scRNA-seq and spatial transcriptomic approaches, we were able to distinguish between viral transgene expression and native gene expression in wild-type animals and identify subpopulations of immune cells to interrogate the key immune interactions involved in GTAU.

In NHP2, the *mScarlet* and *hRPGRco* vectors were delivered as two separate subretinal blebs in distinct quadrants (Figure 1B). Because no fluid-air exchange was performed, bleb migration was not expected. Consistent with this, AF/OCT imaging (Figure 2) showed that both transgene expression and inflammatory signals remained spatially confined to their respective bleb territories. This spatial containment underpins our use of outside-bleb retina as an internal control throughout the study. Across all four eyes from the two NHPs, the total vector dose was held constant at 1.5×10^{11} vg per eye, ensuring comparable ocular and systemic immune exposure between eyes and animals while matching the dose range used clinically for these therapeutic vectors.

The results demonstrate that both clinical-grade AAV2-CAG-*hRPE65* (Luxturna) and engineering-grade AAV8-GRK1-*hRPGRco* were well tolerated *in vivo* with no adverse effects on retinal structure and function. This aligns with the safety profiles of both vectors reported in clinical trials.[2,4] However, the combined immunohistochemistry, spatial transcriptomics, and scRNA-seq data in Figure 3 highlight an important discrepancy between the apparent extent of transgene expression on histology and the actual fractions of cells that are transduced at single-cell resolution. Strong *hRPE65* signal in the RPE—the target cell population for functional rescue in *RPE65*-associated Leber congenital amaurosis (LCA)—was detectable by immunohistochemistry and spatial transcriptomics analysis in NHP1 retina sections. However, within the subretinal bleb area, the AAV2 vector containing the ubiquitous CAG promoter transduced approximately 30% of RPE in our study. This implies that single-cell transcriptomic analysis may be a more objective approach for quantifying cellular level transgene expression within the target tissue, while immunostaining provides a qualitative assessment which is dependent on the affinity of the antibodies used. Moreover, the level of transduction *in vivo* has major clinical implications for the level of visual function improvement and future potential for disease progression. For instance, if 70% of RPE cells in the treated area remained deficient for RPE65, these cells would be unable to complete the visual cycle thus may continue to degenerate. The single-cell data also indicated 35% "off-target" transgene expression among rods. While such ectopic expression of RPE65 may be unhelpful in treating LCA, it could be a desirable characteristic for "biofactory" gene augmentation approaches aimed at producing a secreted protein product (e.g., AAV2-CAG-complement factor I gene therapy for dry AMD).[28] By contrast, the AAV8 vector containing the photoreceptor-specific GRK1 promoter transduced 70%–80% of rods and cones, which represents the majority of the target cell types in *RPGR*-associated XLRP. This could account for the dramatic microperimetry retinal sensitivity improvements seen in clinical trials.[3,4,29] In this instance, the GRK1 promoter robustly restricted transgene expression to photoreceptors, thus minimising off-target effects. Moreover, spatial transcriptomic data indicate the level of AAV-mediate human *RPGR* transgene expression was considerably greater than native macaque *RPGR* expression (Figure 3E). Overexpression of RPGR has previously been reported to be associated with protein mislocalization and potential retinal toxicity in preclinical studies.[30–32] However, we did not find any correlation between the level of transgene expression and upregulation of apoptotic marker genes (*BAX*, *CASP3*, and *APAF1*) at the single-cell level. This somewhat abrogates concerns that transgene overexpression may be a cause of retinal toxicity at the therapeutic dose.[33] We did not find a relationship between inflammation and transgene expression. Although cells from the *mScarlet*-injected bleb contributed a larger share of immune cells in the scRNA-seq dataset (Figure S9C), photoreceptor transgene expression levels were comparable between the *mScarlet* and *hRPGRco* blebs in NHP2 (Figure 3F).

A number of clinical studies have reported incidence of CRA following retinal gene therapy with voretigene neparvovec.[7,34,35] The mechanism of CRA remains unclear. In NHP2, we noted CRA development (seen as confluent hypo-AF and outer retinal thinning) around the area treated with laboratory-grade AAV8-GRK1-*mScarlet* reporter vector (Figure 2, left eye), which was associated with subretinal infiltrates on OCT and the greatest contribution of immune cells to the single-cell analysis (Figures S9C and S9D). This suggests that retinal inflammation can be a potential cause of CRA. Single-cell analysis of immune cells from the

Figure 6. Cytokine expression profile after AAV gene therapy in NHPs

Quantification of vitreous cytokines in NHP1 (A) and NHP2 (B) using the LegendPlex NHP Inflammation Panel with levels expressed as fold-change (log 2) in mean fluorescence intensity (MFI) relative to baseline. RE, right eyes; LE, left eye. (C and D) Single-cell transcriptomic analysis of cytokine gene expression among retinal cell types in NHP1 and NHP2, respectively.

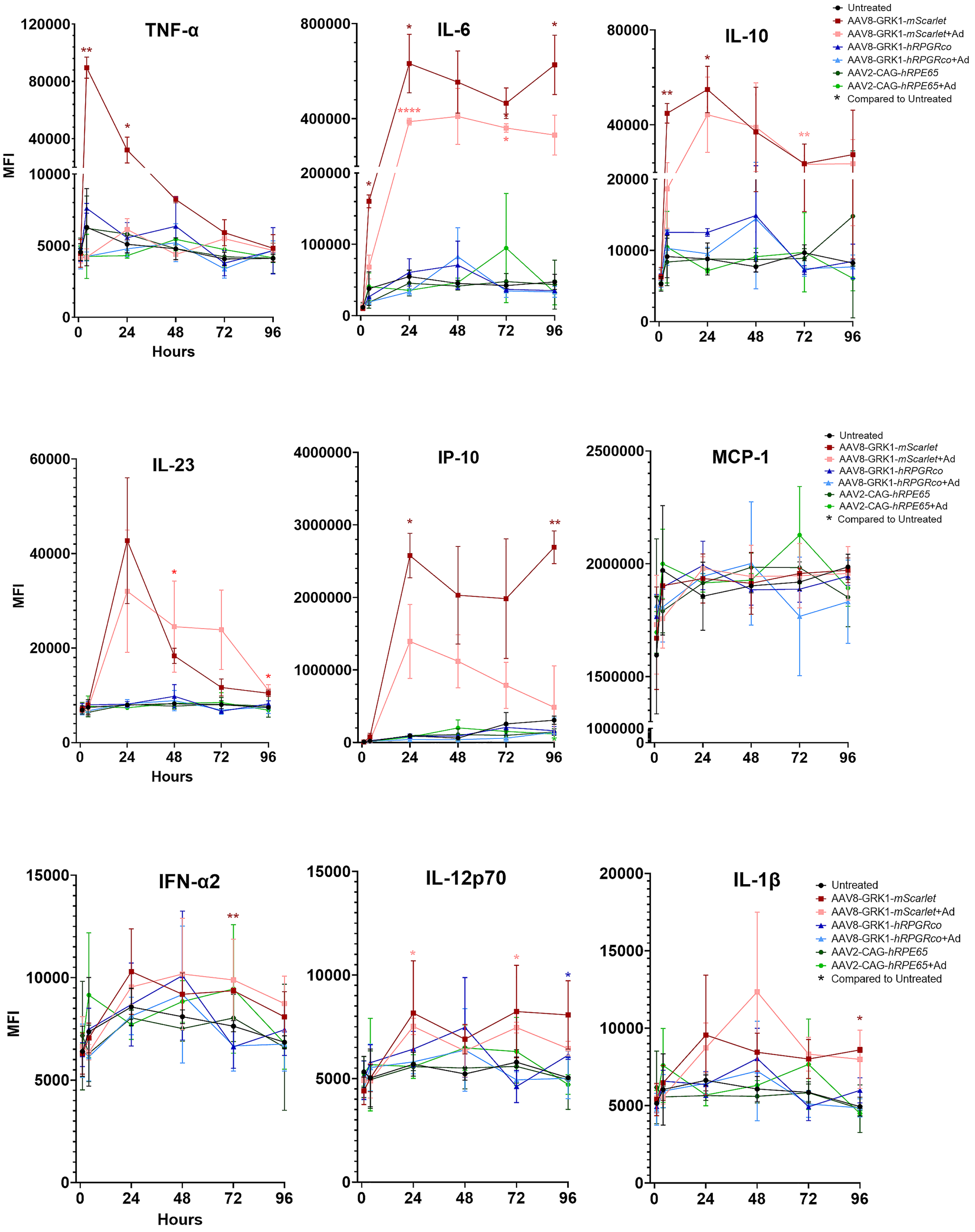

Figure 7. Cytokine changes in human iPSC-derived microglia following exposure to AAV vectors

Cytokine profile of iPSC-microglia treated with AAV2-CAG-hRPE65, AAV8-GRK1-hRPGRco, or AAV8-GRK1-mScarlet vectors, and with (+Ad) or without adalimumab. Untreated iPSC-derived microglia were used as controls. Supernatant was collected at 1, 4, 24, 48, 72, and 96 h. Error bars represent SD (n = 3). two-way ANOVA test was performed: *$p > 0.01$; ** $0.001 < p < 0.01$; ****$p < 0.0001$.

mScarlet vector-treated area revealed upregulation of MHC class I and immunoproteosome-related genes in rods and cones (suggesting increased presentation of intracellular antigens) as well as antiviral response genes (Figure S8). This could promote presentation of capsid and transgene peptides to infiltrating CD8 T cells, triggering targeted photoreceptor destruction. We also observed a type I interferon response with upregulation of IFN-α/β receptor subunit (*IFNAR2*), interferon regulatory factor (*IRF7*), interferon-related genes (*DDX60*, *OAS1*, and *OAS3*) in rods, as well as elevated IFN-β in the vitreous (Figure 6). This could lead to suppression of transgene expression and stimulate myeloid/NK cell antiviral responses. Interestingly, immune infiltration and CRA were confined to the AAV8-GRK1-*mScarlet* bleb, whereas the AAV8-GRK1-*hRPGRco* and untreated regions remained quiescent, arguing against generalized blood-retinal barrier breakdown and making the shared serotype/promoter unlikely drivers of this difference. A transgene-specific contribution remains plausible given the exogenous nature of mScarlet protein, but the very early TNF-α signal in our iPSC-derived microglia in Figure 7 suggests an innate trigger that precedes transgene expression. Vector-associated impurities such as residual host cell proteins or DNA may contribute to the rapid microglial cytokine response seen for this AAV vector.[36]

Our primate transcriptomic data indicate a retinal infiltrate composed mainly of myeloid cells and T cells (Figures 4 and 5, S7B, and S7D). Although many immune cells derived from the mScarlet bleb in NHP2, raising the possibility of responses to a non-mammalian transgene, vitreous proinflammatory cytokines (IP-10/*CXCL10*, MCP-1/*CCL2*, and IL-8/*CXCL8*) were elevated in both NHPs (Figures 6A and 6B). This *CXCL10*-high pattern, together with myeloid APC-associated and T cell-recruiting chemokines, is characteristic of a type-1/IFN-skewed response to subretinal AAV which does not appear to be contingent on the viral transgene.

A type I cell-mediated response against AAV vectors is consistent with previous observations in mice, where subretinal injection of AAV8-CAG-*GFP* led to a similar composition of leukocyte infiltrate detected by flow cytometry.[15] A predominant pro-inflammatory T helper 1 pathway in retina NHP sections was also confirmed by Reichel et al. (2017), including a significant upregulation of *CXCL10* at 4 weeks after AAV injection.[37] *CXCL10* (IP-10) protein was found to be elevated in the vitreous compared to baseline at all three time points from 4 to 12 weeks, especially in NHP2 (Figures 6A and 6B), whereas scRNA-seq performed at 12 weeks showed relatively low level of *CXCL10* mRNA transcripts across retinal myeloid cells. IP-10 is primarily produced by Müller cells, activated microglia and RPE during retinal inflammation.[38] Because *CXCL10* transcription after AAV exposure is transient and peaks at earlier time points, the timing of our sampling likely explains the discordance: by 12 weeks, transcription has returned to baseline while previously secreted protein remains measurable in the vitreous.

Comparison between single-cell data from untreated and treated retinal punches implicates activation of resident myeloid cells by AAV vectors (Figures 5 and S9). Trajectory analysis infers transition of myeloid cells (including microglia) from a resting state toward two activated states characterized by upregulation of (1) antigen presentation and (2) cell motility gene sets. An acquired antigen-presenting function in microglia would be expected to restimulate AAV antigen-specific infiltrating T cells. Enhanced mobility would enable migration of microglia to the subretinal space, a phenomenon previously suggested by Xiong et al.[39] Recent work shows infiltrating monocytes can acquire microglia-like features in the retina, including expression of P2RY12 and *AIF1*/IBA1.[40] IBA1 and P2RY12 staining are therefore not microglia exclusive (Figure 4) as these markers could also label infiltrating macrophages (which could account for the detection of monocytic phagocytes in the choroid and sclera in the spatial transcriptomic data). However, at 12 weeks, we observed very few marker-positive microglia in the inner and outer plexiform layers, while monocytic phagocytes appeared enriched in the subretinal region. This spatial pattern may represent prior redistribution of resident microglia toward the subretinal space following subretinal AAV administration or infiltration of macrophages from the choroid. Since IBA1 gene expression can change with microglia activation, the data do not enable clear differentiation between these two hypotheses. P2RY12 and *HEXB* expression markers decrease in activated microglia. However, the resolution of the Visium platform limited sensitivity to detect less abundant states. *TMEM119* and *AIF1*/IBA1 staining of active microglia identified only rare positive cells within the *hRPE65* and *hRPGRco* vector-treated retinas at 12 weeks (data not shown), supporting the interpretation that the strong P2RY12 and HEXB signal seen in Figures 4A and 4C reflects the majority of microglia captured in this experiment.

Whether the two branches based on trajectory inference are truly separate, or whether the motility gene expression program is an intermediate on the path to an antigen presenting phenotype, requires further exploration. Further insights into the role of microglia in GTAU were gained from human iPSC-derived microglia stimulated with AAV vectors. AAV exposure led to a rapid increase in microglia phagocytic activity and release of inflammatory cytokines, including TNF-α, IFN-α2, IL-6, and MCP-1. MCP-1 was consistently identified as a key monocyte chemoattractant expressed by myeloid cells in the NHP retinas by single-cell analysis and a prominent cytokine in vitreous biopsies but not in the first 96 h after AAV exposure in iPSC-derived microglia (Figure 7). Together, these results implicate retinal microglia as a key orchestrator of immune response against AAV and recruiter of adaptive immunity.

At 12 weeks post-subretinal gene therapy, the infiltrating T cells in the retina showed signs of exhaustion similar to those seen during chronic viral infection, with high expression of cytotoxicity genes and immune checkpoints (*PD1*, *TIGIT*, and *ICOS*) (Figure S11D and S11E). We also detected a subset of $CD8^+$ effector memory T cells. The extent to which these cells are actively killing transduced retinal cells remains to be determined. Their presence carries

significant clinical implications for second eye treatment in the same patient as re-challenge with the same antigen may lead to more severe and prolonged retinal inflammation. Interestingly, we also found evidence of both Tregs and IL-10 + T cells, which are likely dampening the ongoing immune response. Understanding the interactions between these subsets of T cells and the myeloid cells will be important in developing more effective prophylaxis and treatment for GTAU.

The use of adjunctive intravitreal adalimumab at 4 weekly intervals had limited impact on the immune response seen *in vivo* at 12 weeks over and above the effects of a single dose of periocular corticosteroid (triamcinolone) at the time of gene therapy surgery. Given the small number of eyes and the concentration of inflammation in one animal, the study was not powered to evaluate intravitreal anti-TNF-α. However, adalimumab did suppress the transient TNF-α rise (first 4 h) in human iPSC-microglia exposed to the AAV8-GRK1-*mScarlet* preparation *in vitro* (Figure 7), suggesting that while TNF-α may be part of the early response to vector exposure, it may not play a major role in chronic GTAU. The vitreous profile instead suggests CXCL10, IL-6, and CXCL8 pathways as potential candidates for subsequent studies.

In conclusion, our appreciation of the cellular effects of AAV gene therapy in human eyes has so far been limited by the "black box" effect of the treatment in patients. This study provides deep insight into the nature of immune response to AAV-mediated retinal gene therapy in primates using a combination of spatial and single-cell transcriptomic approaches. Spatial transcriptomics has gained significant traction in recent years to bridge the gap between structural and transcriptomic analyses but has seen limited application to the retina to date in the mouse and human.[41,42] To our knowledge, the present study is the first to apply spatial transcriptomics to the NHP retina. However, this exploratory study in two macaques (four AAV-treated eyes) carries a number of limitations. A limitation of spatial and single-cell transcriptomic studies in NHPs is the incomplete annotation of the *Macaca mulatta* genome relative to the human reference. While rhesus macaques are phylogenetically close to humans and represent a highly relevant model for studying immune responses, the characterization of immune-related genes in the macaque genome remains an evolving field. Immunoglobulin heavy chain genes in particular are not yet fully described in current macaque reference databases, meaning that B cell-related transcriptomic signals may be underrepresented in our dataset. As such, while our data robustly capture key inflammatory signatures including T cell infiltration, chemokine expression, and glial reactivity, conclusions regarding the full breadth of the humoral immune response should be drawn with caution, and future studies will benefit from continued improvements in macaque genome annotation. Nonetheless, our single-cell and spatial datasets combine to provide cell-type and location-resolved insight into AAV-associated inflammation in the primate retina. This resource helps prioritize targets for GTAU mitigation in humans and informs sampling windows and biomarkers relevant to human gene therapy.

MATERIALS AND METHODS

AAV vectors

The AAV2-CAG-*hRPE65* vector (voretigene neparvovec-rzyl, Luxturna, Spark Therapeutics, Inc., USA) was GMP-grade vector surplus from approved human retinal gene therapy. The AAV8-GRK1-*hRPGRco* vector was produced at the Nationwide Children's Hospital (Columbus, OH, USA) Vector Core as an "engineering grade" batch for pre-clinical studies.[3]

The *mScarlet*-expressing AAV vectors used for NHPs subretinal injections and iPSC-derived microglia experiments (AAV8-GRK1-*mScarlet*) were produced via transient transfection of adherent HEK293T cells. Confluent HYPERflasks (Corning Life Sciences, Tewksbury, MA, USA) were transfected with the pDG RepCap plasmid (Plasmid Factory, Bielefeld, Germany) of appropriate serotype and the transgene plasmid in TransIT-VirusGEN Transfection Reagent (Mirus bio, Madison, WI, USA). HYPERflasks were harvested 72 h post-transfection and the cell pellet lysed (lysis buffer 1 M Tris, 150 mM NaCl, cOmplete Protease Inhibitor (Roche, Basel, Switzerland) in water, pH 8.5). Lysate was subjected to three freeze-thaw cycles and Benzonase (Merck, Darmstadt, Germany) treated. AAV particles were isolated via an ultracentrifugation iodixanol gradient then concentrated and buffer exchanged for phosphate-buffered saline (PBS) with an Amicon Ultra 100 k filter unit (Merck Millipore, Burlington, MA, USA). Titer was determined by qPCR with primers specific to the transgene (see Table S1). The *mScarlet* vector preparation was confirmed to be under the limit of detection of endotoxin (<0.10 EU/mL) with the Pierce Chromogenic Endotoxin Quant Kit (https://www.thermofisher.com/order/catalog/product/A39552), and *in vivo* safety was pre-tested by subretinal administration of 1.5×10^9 vg in mice without observable adverse effects on OCT.

Engineering-grade AAV8-GRK1-*hRPGRco* vector and research-grade AAV8-GRK1-*mScarlet* vector were compared for purity and contaminating protein content by SDS-PAGE (Figure S15). Briefly, 10 μL of AAV stock was mixed with 5 μL 5× protein loading buffer in 10 μL of PBS and denatured at 95°C for 10 min. Samples were loaded onto a 10% Criterion TGX Precast Protein gel (Bio-Rad) alongside 10 μL of BLUye Prestained Protein ladder (Sigma-Aldrich). Gels were run for 105 min at 100 V in 1× SDS running buffer. Gels were washed three times in water for 5 min on a shaker and then submerged in Bio-Safe Coomassie G-250 stain (Bio-Rad) for 2 h. The gel was washed three more times with water and then imaged with LICOR Odyssey Imager (LICOR). AAV capsid protein bands are expected at ∼87 kDa (VP1), ∼72 kDa (VP2), and ∼62 kDa (VP3).

Animals

Two rhesus macaques (*Macaca Mulatta*) aged 5 years (NHP1) and 6 years (NHP2), obtained from the Medical Research Council (MRC) Centre for Macaques (Harwell Institute, Salisbury, UK), were used in this study. Animals were handled in accordance with The Animals (Scientific Procedures) Act 1986 and UK Home Office regulations. All procedures were performed under general anesthesia with

sevoflurane and medetomidine, with additional ketamine IV infusion for electroretinograms (ERGs). At baseline, 5 mL of blood was collected. cSLO, OCT, and blue-light AF imaging were performed with Heidelberg Spectralis HRA (Heidelberg Engineering, Heidelberg, Germany) and analyzed with Heidelberg Eye Explorer (HEYEX). Due to the orientation of the camera, all OCT images are inverted such that the inferior retina appears at the top of the images and the superior retina appears at the bottom. Light and dark-adapted baseline ERGs were obtained using a RETeval (LKC Technologies, Gaithersburg, MD, USA).

25-gauge pars plana vitrectomy was performed in all eyes with subretinal injection of AAV vectors using 38G subretinal cannula (MedONE, Sarasota, FL, USA) and foot-pedal-controlled viscous fluid injection (Alcon Constellation, Alcon, Fort Worth, TX, USA), as per human retinal gene therapy surgery.[43] NHP1 received 1.5×10^{11} vg of *voretigene neparvovec* via two subretinal injection blebs in both eyes (Figure 1). NHP2 received subretinal injections of 1.25×10^{11} vg of AAV8-GRK1-*hRPGRco* and 2.5×10^{10} vg of AAV8-GRK1-*mScarlet* (a "shadow" vector construct expressing a fluorescent reporter) in two separate blebs to each eye. At the end of the procedure, all four eyes of the two NHPs received subtenon injection of 40 mg triamcinolone acetate (Kenalog, Bristol Myers Squibb, Princeton, NJ, USA) and topical dexamethasone/neomycin/polymyxin B (Maxitrol, Southfield, MI, USA) ointment as standard. In addition, both left eyes received intravitreal injection of 1.5 mg of the humanized anti-TNF-α antibody, adalimumab (Amgevita, Amgen Limited, Cambridge, UK). No additional drops or systemic immunosuppression was given.

Based on the actual vector dose injected, areas of subretinal blebs measured on AF images, and an assumed RPE cell density of 4,000 cells/mm^2,[20] we estimated the vector dose per RPE cell for each treated area (Table 1). Blood sampling and retinal imaging were performed at baseline (before AAV subretinal injections) and every 2 weeks thereafter. Vitreous biopsy was obtained at baseline then every 4 weeks. Light and dark-adapted ERGs were obtained at baseline, 6 and 10 weeks. Intravitreal injection of 1.5 mg of adalimumab to the left eyes was repeated at 4 weekly intervals. The animals were euthanized, and eyes and spleen were harvested at 12 weeks.

Cytokine assays on NHP vitreous and blood samples

NHP serum and vitreous samples were assessed using the LEGENDplex NHP Inflammation Panel with V-bottom Plate (BioLegend, San Diego, CA, USA) according to manufacturer's instructions. Briefly, serum or vitreous fluid samples were centrifuged at 500 *g* for 5 min to clear then snap frozen on dry ice for storage at -80°C to be analyzed with samples from all time points together later. Vitreous samples were all run undiluted, whereas serum samples were diluted 1:4 in assay buffer as per the manufacturer's recommendations. 25 μL of sample was incubated with pre-defined mixed beads in assay buffer for 2 h at room temperature with 800 rpm shaking. Samples were spun down at 250 *g* for 5 min, supernatant discarded, and washed in wash buffer twice. Detection antibodies were added and incubated for 1 h at room temperature with 800 rpm shaking. SA-PE was added directly to sample wells and incubated for 30 min at room temperature with 800 rpm shaking. Samples were spun down and washed as mentioned previously, followed by final resuspension in 150 μL wash buffer. Samples were run on the Cytek Aurora's 5-Laser Full Spectrum Cytometer 16UV-16V-14B-10YG-8R (Fremont, CA, USA), set up as per the manufacturer's instructions to gate for the specified bead populations (www.biolegend.com/legendplex). Data were analyzed in the LEGENDplex Data Analysis Software (Qognit, BioLegend).

Dissection and tissue preparation

After rapid harvesting of the globes, the cornea and the lens were first carefully removed. The posterior eyecup was flattened with four diagonal radial cuts and the remaining vitreous carefully removed. The flattened eyecup was transferred to a glass slide. Guided by vascular landmarks on retinal images, 4 mm full-thickness retina/choroid/sclera punches were obtained using skin biopsy punches corresponding to treated and untreated areas and numerically assigned for downstream processing. For single-cell transcriptomic analysis, the retina was lifted away from the underlying RPE/Bruch's membrane of the biopsy punch for dissociation and library preparation. For spatial transcriptomic analysis, full-thickness punches were embedded in optimal cutting temperature compound (VWR, Radnor, PA, USA) and snap frozen in isopentane for storage (at -80 °C) or sectioning as per 10× Genomics protocol. For immunohistochemistry, additional full-thickness punches were incubated in pre-chilled 4% PFA for 30 min at room temperature, then transferred to 1% PFA and stored at 4°C until processing.

Immunohistochemistry and confocal microscopy

Retinal biopsy punches fixed in 1% PFA were cryosectioned to 10 μm slices at −20°C onto Superfrost plus slides (VWR). Slides were washed with PBS. For sections stained with anti-IBA1 (Fujifilm Wako, Osaka, Japan), an additional antigen retrieval step was performed. Briefly, slides were immersed in a pre-heated solution of 10 mM citrate buffer (Thermo Fisher Scientific, Waltham, MA, USA) and 0.05% Tween 20 and then heated several times at high temperature. After cooling the slides at room temperature for 20 min, samples were washed with 0.05% Tween 20 in PBS. All conditions were blocked with 10% Normal Donkey Serum (NDS), 0.05% Triton X-100 in PBS. Slides were then incubated overnight at 4°C with primary antibodies (full list in Table S2) in a solution composed of 1% NDS, 0.05% Triton X-100, and PBS. The next day, slides were washed with 0.05% Tween 20 in PBS and incubated with secondary antibodies under dark conditions for 2 h at room temperature. Slides were then briefly washed with 0.05% Tween 20 in PBS before being counterstained with Hoechst diluted at 1:1000 for 15 min in the dark. Coverslips were mounted in SlowFade Diamond Antifade Mountant (Thermo Fisher Scientific) and sealed. z stack images were captured on a Zeiss LSM 710 confocal microscope and post-analysis was performed in Fiji.[44]

Retinal dissociation, single-cell library preparation, and sequencing

Retinal biopsies were dissociated using the Worthington Papain Dissociation system (Worthington, Lakewood, NJ, USA). Retinas were placed in a solution of 20 U/mL papain, 0.005% DNase I with 1 mM L-cysteine and 5 mM EDTA in Earle's balanced salt solution (EBSS) for 10 min at 37°C with frequent, gentle agitation. Samples were then diluted by addition of 500 μL of EBSS to inactivate the papain and centrifuged at 300 *g* for 5 min at room temperature. Pellets were resuspended in 525 μL of a solution containing 1 mg/mL ovomucoid and BSA and 100 U/mL DNase I in EBSS. The resulting suspension was carefully layered over 500 μL ovomucoid/BSA solution and centrifuged at 70 *g* for 6 min. Supernatant was discarded and cells were resuspended in PBS containing 0.04% BSA.

Library preparation for scRNA-seq analysis of dissociated retinal cells was performed using the Chromium Next GEM 10X Single-cell 5′ v2 Dual Index Kit (10× Genomics, Pleasanton, CA, USA), which includes post GEM-generation clean-up, cDNA amplification and DNA quantification. Libraries were quality controlled and sequenced by Novogene (Cambridge, UK) using the Illumina NovaSeq X (Illumina, San Diego, CA, USA). Transformed raw sequencing data were provided as FASTQ files. The Cellranger MkRef pipeline from 10× Genomics was used to build a custom reference genome using FASTA and GTF files for the Mmul_10 *Macaca mulatta* reference genome appended with additional AAV transgene sequences. Cellranger count was then used to perform alignment, filtering, barcode counting, and UMI (unique molecular identifier) counting from FASTQ files to the custom reference genome. This generated feature-barcode matrices for each sample, which was used for downstream analyses.

Analysis of NHP scRNA-seq data

The SoupX package was used to correct raw feature-barcode matrices for ambient RNA contamination,[45] followed by doublet detection with scDblFinder.[46] Corrected matrices were then used for analysis with Scanpy.[47] Commonly used QC metrics, such as UMI count, number of features, percentage mitochondrial RNA and percentage ribosomal RNA were used to filter out low-quality cells, and doublets called by scDblFinder were removed. Samples were then log normalized and highly variable genes were selected. Harmony integration was then performed to remove batch effects and generate a single feature barcode matrix.

The integrated matrix was passed through standard dimensionality reduction and clustering pipelines in Seurat.[48] Briefly, principal-component analysis (PCA) was used to determine dataset dimensionality, followed by shared nearest-neighbor graph construction and dimensionality reduction with the uniform manifold approximation projection (UMAP) method. Annotated clusters were individually subclustered and iteratively re-processed to further remove low-quality droplets and doublets. Differential expression was performed by pseudobulking each identified cell type by sample with decoupleR and analyzing with the DESeq2 package.[49,50] Ranked gene lists from DESeq2 output were used for gene set enrichment analysis using the Molecular Signature Database (MSigDB) Gene Ontology Biological Process gene sets with the fgsea package in R.[51] Trajectory inference was performed on myeloid cells with the scFates package in Python following the tree analysis guidelines.[52] Prior to trajectory inference, the force-directed graph of myeloid cells was drawn using the "draw_graph" function in Scanpy with the Fruchterman-Reingold algorithm.[53] The ProjecTILs package was used for projecting the T cell subset onto the annotated ProjecTILs dataset using the standard pipeline.[27]

Spatial transcriptomic library generation, processing, and analysis

For spatial transcriptomics assays, the Visium Spatial v.1 3′ Gene Expression technology (10× Genomics) based on polyA capture was applied. Following optimization, fresh-frozen retinal punch biopsies from NHP1 and NHP2 were sectioned at 10 μm with a cryostat and positioned on a Visium (10× Genomics) spatial gene expression slide. The Visium slide was fixed in pre-chilled methanol at −20°C for 30 min, and stained with Hematoxylin & Eosin (H&E). In parallel, RNA from ten adjacent tissue sections were extracted using RNAse-Free DNase kit (Qiagen, Hilden, Germany) and RNA quality checked using Agilent Tapestation (Agilent Technologies, Santa Clara, CA, USA). The H&E-stained retina sections were imaged with the Zeiss Axioscan 7 microscope slide scanner (Zeiss, Oberkochen, Germany). Following 10X Genomics protocol, retina sections were incubated for permeabilization for 12 min at 37°C and reverse transcription was performed. Next, second strand cDNA was synthetized and total cDNA was denatured. qPCR was performed using 10× Genomics' specific cDNA primers for cDNA quantification before cDNA amplification and clean-up. The next day, cDNA integrity was measured. Spatial gene expression library was finally constructed and sequenced with Illumina NovaSeq X Plus by Novogene.

FASTQ files containing sequencing reads, previously generated custom Mmul_10 reference genome with additional AAV transgene sequences and TIF image files of H&E-stained retina sections were used as input to the Spaceranger count function. Combined data were analyzed with 10× Genomics' Loupe browser software. Since the Visium v.1 3′ Gene Expression platform is based on polyA capture, human and macaque transcripts can be clearly distinguished: human *RPE65* or codon-optimized *RPGR* transcripts were markers of AAV-derived human transgenes, while endogenous *Macaca mulatta RPE65* or *RPGR* transcripts aligned specifically with the *Macaca mulatta* reference genome.

Differentiation of human iPSC to microglia

Cells were cultured at 37°C in 5% CO_2. Healthy human iPSCs (BIONi037-A, Bioneer, Hørsholm, Denmark) were differentiated into microglia using previously described protocol.[54] Briefly, batch-QCed iPSCs were plated in Geltrex-coated well plates in mTeSR1 medium (STEMCELL Technologies, Vancouver, Canada) supplemented with Rho kinase inhibitor upon thaw to maintain

viability during single-cell suspension (Y27632 Abcam, Cambridge, UK). The cell medium was changed daily, then cells were centrifuged at 400 *g* into AggreWell 800 plate (STEMCELL Technologies) with embryoid body (EB) medium (mTeSR1 medium supplemented with BMP4 at 50 ng/mL (Invitrogen, Thermo Fisher Scientific); VEGF at 50 ng/mL (Invitrogen); SCF at 20 ng/mL (Miltenyi Biotec, Bergisch Gladbach, Germany); and penicillin-streptomycin (P/S) at 100X (Gibco, Thermo Fisher Scientific). After 4 days with daily feeding, EBs were separated from debris with a 40 μM cell strainer and plated in T175 flasks with myeloid differentiation medium: X-VIVO 15 cell media (Lonza, Basel, Switzerland) supplemented with 1X GlutaMax (Gibco, Thermo Fisher Scientific), 1X 2-Mercapto-ethanol (Gibco), M-CSF at 100 ng/mL (PHC9501, Invitrogen), and IL-3 at 25 ng/mL (Invitrogen). Differentiation cultures were fed weekly and emergent primitive macrophage precursors were harvested from the supernatant 8 weeks after for microglia differentiation. Primitive macrophage precursors were separated from EBs with a 40 μM cell strainer, counted and resuspended in microglia medium: 1X Advanced DMEM/F12 (Thermo Fisher Scientific) supplemented with 1X Glutamax, M-CSF at 25 ng/mL, GM-CSF at 10 ng/mL (Invitrogen), TGFB1 at 50 ng/mL (Peprotech, Cranbury, NJ, USA), IL-34 at 100 ng/mL (Peprotech), and P/S at 50 U/mL. Macrophage precursors were then plated in 96-well plates, and a 50% media change was performed tri-weekly for 2 weeks. After macrophage precursor differentiation into microglia, cells were collected for assays.

Phagocytosis assay and cytokine profiling of iPSC-derived microglia

For phagocytosis assays, all iPSC-derived microglia were treated with pHrodo Green Zymosan Bioparticles (Thermo Fisher Scientific) as phagocytic cargo. Control cells were treated with zymosan only. The “AAV8-treated 72 h prior” were transduced with an AAV8-CAG-*mScarlet*-WPRE vector at a multiplicity of infection (MOI) of 10,000 seven days after microglia differentiation. 72 h after transduction, the media was changed, and cells were washed three times by gently pipetted fresh media onto and swirled the plate to remove any residual AAV vector prior to induction of phagocytosis. The “AAV8-treated fresh” were stimulated with AAV8-CAG-*mScarlet*-WPRE present in cell media during the phagocytosis assay only. The “lipopolysaccharide (LPS)-treated” cells were included as a positive control. Four replicate wells were included per condition. The phagosome acidification index was determined by fluorescence microscopy as the total amount of pHrodo Green signal above threshold for each time point over the number of cells and processed by Fiji.

As performed for the NHP vitreous and blood samples, a similar LEGENDplex Cytokine assay was performed on the human iPSC-derived microglia. Cells were plated in 96-well plates in microglia medium. At baseline, previously described AAV vectors (AAV2-CAG-*hRPE65*, AAV8-GRK1-*hRPGRco*, or AAV8-GRK1-*mScarlet*) were added to the cells at an MOI of 10,000. In some wells, adalimumab (Amgevita 20 mg, Amgen Limited) was also added to the supernatant at a final concentration of 1 μg/mL. Supernatant was then collected at 1, 4, 24, 48, 72, and 96 h, assessed using the LEGENDplex Human Inflammation Panel (BioLegend) or the LEGENDplex Human *CXCL10* (IP-10) Capture Bead A5 (BioLegend), and analyzed as previously described.

DATA AND CODE AVAILABILITY

Code used for analysis is available on GitHub (https://github.com/JoelQuinn). Raw and processed scRNA-seq files have been deposited in NCBI’s Gene Expression Omnibus and are accessible through GEO series accession number GSE304621. Raw and processed Visium spatial RNA-seq files are accessible through GEO series accession number GSE304504.

ACKNOWLEDGMENTS

This work was supported by grant funding from the Wellcome Trust (216593/Z/19/Z). K.X., J.C.-K., and J.Q. would like to acknowledge additional funding from the Medical Research Council (MR/Z504725/1, MR/X013189/1, and MR/N013468/1). M.C.J. would like to acknowledge funding from the Biotechnology & Biological Sciences Research Council (BB/X511353/1). K.X. is indebted to Prof. Alistair Lamb (Nuffield Department of Surgical Sciences, University of Oxford) for advice on spatial transcriptomic analysis, Prof. Andrew Dick (UCL Institute of Ophthalmology), and Prof. M Dominik Fischer (Nuffield Department of Clinical Neurosciences, University of Oxford) for discussions over NHP experimental plans. We thank the staff members of Biomedical Services, Lucy Underdown, Dr. Henri Bertrand, Katie Underdown, Sarah Rohling, Andrew Emberton, and Kelly Simpson for their expert care of the animals and assistance with anesthesia. We are also grateful to Dr. Helen Ferry at the Flow Sorting Facility (Experimental Medicine Division, University of Oxford) for expert technical assistance with flow cytometric analysis.

AUTHOR CONTRIBUTIONS

K.X. conceptualized, planned, and supervised the project. C.M.-F.d.l.C. provided AAV vectors and performed dissection and tissue processing for immunohistochemistry and spatial transcriptomics. K.X. performed gene therapy surgery with assistance from J.C.-K. and R.E.M. C.S. and S.A.C. produced iPSC-derived microglia. M.C.J. produced the *mScarlet* vector and conducted immunological analyses. J.Q. performed single-cell RNA-sequencing and bioinformatic analysis. C.S. performed immunohistochemistry, spatial transcriptomic processing, and analysis. C.S. and K.X. wrote the original manuscript, and all authors contributed to the editing and reviewing process.

DECLARATION OF INTERESTS

K.X. is co-I on clinical trials sponsored by Beacon Therapeutics, AAVantgarde Bio and AbbVie. J.C.-K. is a PI on clinical trial sponsored by Beacon Therapeutics. R.E.M. is a scientific co-founder and consultant to Beacon Therapeutics. R.E.M. is a named co-inventor on a patent for RPGR gene therapy owned by the University of Oxford. C.M.-F.d.l.C. has received grant funding from Beacon Therapeutics and the Macular Society UK for work on RPGR gene therapy.

SUPPLEMENTAL INFORMATION

Supplemental information can be found online at https://doi.org/10.1016/j.omta.2026.201726.

REFERENCES

1. Purdy, R., John, M., Bray, A., Clare, A.J., Copland, D.A., Chan, Y.K., Henderson, R.H., Nerinckx, F., Leroy, B.P., Yang, P., et al. (2025). Gene Therapy-Associated Uveitis (GTAU): Understanding and mitigating the adverse immune response in retinal gene therapy. Prog. Retin. Eye Res. *106*, 101354. https://doi.org/10.1016/j.preteyeres.2025.101354.

2. Russell, S., Bennett, J., Wellman, J.A., Chung, D.C., Yu, Z.F., Tillman, A., Wittes, J., Pappas, J., Elci, O., McCague, S., et al. (2017). Efficacy and safety of voretigene neparvovec (AAV2-hRPE65v2) in patients with RPE65-mediated inherited retinal dystrophy: a randomised, controlled, open-label, phase 3 trial. Lancet *390*, 849–860. https://doi.org/10.1016/s0140-6736(17)31868-8.

3. Cehajic-Kapetanovic, J., Xue, K., Martinez-Fernandez de la Camara, C., Nanda, A., Davies, A., Wood, L.J., Salvetti, A.P., Fischer, M.D., Aylward, J.W., Barnard, A.R., et al. (2020). Initial results from a first-in-human gene therapy trial on X-linked retinitis pigmentosa caused by mutations in RPGR. Nat. Med. *26*, 354–359. https://doi.org/10.1038/s41591-020-0763-1.

4. Lam, B.L., Pennesi, M.E., Kay, C.N., Panda, S., Gow, J.A., Zhao, G., and MacLaren, R.E.; XIRIUS Study Group (2024). Assessment of Visual Function with Cotoretigene Toliparvovec in X-Linked Retinitis Pigmentosa in the Randomized XIRIUS Phase 2/3 Study. Ophthalmology *131*, 1083–1093. https://doi.org/10.1016/j.ophtha.2024.02.023.

5. Yang, P., Birch, D., Lauer, A., Sisk, R., Anand, R., Pennesi, M.E., Iannaccone, A., Yaghy, A., Scaria, A., Jung, J.A., et al. (2025). Subretinal Gene Therapy Drug AGTC-501 for XLRP Phase 1/2 Multicenter Study (HORIZON): 24-Month Safety and Efficacy Results. Am. J. Ophthalmol. *271*, 268–285. https://doi.org/10.1016/j.ajo.2024.11.021.

6. Fischer, M.D., Ochakovski, G.A., Beier, B., Seitz, I.P., Vaheb, Y., Kortuem, C., Reichel, F.F.L., Kuehlewein, L., Kahle, N.A., Peters, T., et al. (2019). Efficacy and Safety of Retinal Gene Therapy Using Adeno-Associated Virus Vector for Patients With Choroideremia: A Randomized Clinical Trial. JAMA Ophthalmol. *137*, 1247–1254. https://doi.org/10.1001/jamaophthalmol.2019.3278.

7. Fischer, M.D., Simonelli, F., Sahni, J., Holz, F.G., Maier, R., Fasser, C., Suhner, A., Stiehl, D.P., Chen, B., Audo, I., et al. (2024). Real-World Safety and Effectiveness of Voretigene Neparvovec: Results up to 2 Years from the Prospective, Registry-Based PERCEIVE Study. Biomolecules *14*, 122.

8. MacLaren, R.E., Fischer, M.D., Gow, J.A., Lam, B.L., Sankila, E.-M.K., Girach, A., Panda, S., Yoon, D., Zhao, G., and Pennesi, M.E. (2023). Subretinal timrepigene emparvovec in adult men with choroideremia: a randomized phase 3 trial. Nat. Med. *29*, 2464–2472. https://doi.org/10.1038/s41591-023-02520-3.

9. Pierce, E.A., Aleman, T.S., Jayasundera, K.T., Ashimatey, B.S., Kim, K., Rashid, A., Jaskolka, M.C., Myers, R.L., Lam, B.L., Bailey, S.T., et al. (2024). Gene Editing for *CEP290*-Associated Retinal Degeneration. N. Engl. J. Med. *390*, 1972–1984. https://doi.org/10.1056/NEJMoa2309915.

10. Campochiaro, P.A., Avery, R., Brown, D.M., Heier, J.S., Ho, A.C., Huddleston, S.M., Jaffe, G.J., Khanani, A.M., Pakola, S., Pieramici, D.J., et al. (2024). Gene therapy for neovascular age-related macular degeneration by subretinal delivery of RGX-314: a phase 1/2a dose-escalation study. Lancet *403*, 1563–1573. https://doi.org/10.1016/S0140-6736(24)00310-6.

11. Kessel, L., Christensen, U.C., and Klemp, K. (2022). Inflammation after Voretigene Neparvovec Administration in Patients with RPE65-Related Retinal Dystrophy. Ophthalmology *129*, 1287–1293. https://doi.org/10.1016/j.ophtha.2022.06.018.

12. Yang, P., Pardon, L.P., Ho, A.C., Lauer, A.K., Yoon, D., Boye, S.E., Boye, S.L., Roman, A.J., Wu, V., Garafalo, A.V., et al. (2024). Safety and efficacy of ATSN-101 in patients with Leber congenital amaurosis caused by biallelic mutations in GUCY2D: a phase 1/2, multicentre, open-label, unilateral dose escalation study. Lancet *404*, 962–970. https://doi.org/10.1016/S0140-6736(24)01447-8.

13. Reichel, F.F., Seitz, I., Wozar, F., Dimopoulos, S., Jung, R., Kempf, M., Kohl, S., Kortüm, F.C., Ott, S., Pohl, L., et al. (2023). Development of retinal atrophy after subretinal gene therapy with voretigene neparvovec. Br. J. Ophthalmol. *107*, 1331–1335. https://doi.org/10.1136/bjophthalmol-2021-321023.

14. Seitz, I.P., Wozar, F., Ochakovski, G.A., Reichel, F.F., Gelisken, F., Bartz-Schmidt, K.U., Peters, T., and Fischer, M.D. (2024). Dose-Dependent Progression of Chorioretinal Atrophy at the Injection Site After Subretinal Injection of rAAV2/8 in Nonhuman Primates. Ophthalmol. Sci. *4*, 100516. https://doi.org/10.1016/j.xops.2024.100516.

15. Chandler, L.C., McClements, M.E., Yusuf, I.H., Martinez-Fernandez de la Camara, C., MacLaren, R.E., and Xue, K. (2021). Characterizing the cellular immune response to subretinal AAV gene therapy in the murine retina. Mol. Ther., Methods Clin. Dev. *22*, 52–65. https://doi.org/10.1016/j.omtm.2021.05.011.

16. Durrani, K., Kempen, J.H., Ying, G.S., Kacmaz, R.O., Artornsombudh, P., Rosenbaum, J.T., Suhler, E.B., Thorne, J.E., Jabs, D.A., Levy-Clarke, G.A., et al. (2017). Adalimumab for Ocular Inflammation. Ocul. Immunol. Inflamm. *25*, 405–412. https://doi.org/10.3109/09273948.2015.1134581.

17. Bober, E., Frain, K., Fotuhi, M., Virgo, J., Hindle, E., Ma, J., Luis, J., Addison, P., Okhravi, N., Tucker, W., et al. (2024). Adalimumab in the treatment of refractory non-infectious scleritis: 6-month outcomes. Eye *38*, 628–630. https://doi.org/10.1038/s41433-023-02725-3.

18. Çam, F., and Celiker, H. (2024). Efficacy, retention rate and safety of adalimumab treatment in patients with non-infectious uveitis and scleritis: a real-world, retrospective, single-centre study. Eye *38*, 893–901. https://doi.org/10.1038/s41433-023-02800-9.

19. Suhler, E.B., Adán, A., Brézin, A.P., Fortin, E., Goto, H., Jaffe, G.J., Kaburaki, T., Kramer, M., Lim, L.L., Muccioli, C., et al. (2018). Safety and Efficacy of Adalimumab in Patients with Noninfectious Uveitis in an Ongoing Open-Label Study: VISUAL III. Ophthalmology *125*, 1075–1087. https://doi.org/10.1016/j.ophtha.2017.12.039.

20. Snodderly, D.M., Sandstrom, M.M., Leung, I.Y.F., Zucker, C.L., and Neuringer, M. (2002). Retinal Pigment Epithelial Cell Distribution in Central Retina of Rhesus Monkeys. Investig. Ophthalmol. Vis. Sci. *43*, 2815–2818.

21. Brouwer, A.H., de Wit, G.C., de Boer, J.H., and van Genderen, M.M. (2020). Effects of DTL electrode position on the amplitude and implicit time of the electroretinogram. Doc. Ophthalmol. *140*, 201–209. https://doi.org/10.1007/s10633-019-09733-3.

22. Brouwer, A.H., de Wit, G.C., Ten Dam, N.H., Wijnhoven, R., van Genderen, M.M., and de Boer, J.H. (2019). Prolonged Cone b-Wave on Electroretinography Is Associated with Severity of Inflammation in Noninfectious Uveitis. Am. J. Ophthalmol. *207*, 121–129. https://doi.org/10.1016/j.ajo.2019.05.028.

23. Berson, E.L., Sandberg, M.A., Rosner, B., Birch, D.G., and Hanson, A.H. (1985). Natural course of retinitis pigmentosa over a three-year interval. Am. J. Ophthalmol. *99*, 240–251. https://doi.org/10.1016/0002-9394(85)90351-4.

24. Beltran, W.A., Cideciyan, A.V., Boye, S.E., Ye, G.-J., Iwabe, S., Dufour, V.L., Marinho, L.F., Swider, M., Kosyk, M.S., Sha, J., et al. (2017). Optimization of Retinal Gene Therapy for X-Linked Retinitis Pigmentosa Due to RPGR Mutations. Mol. Ther. *25*, 1866–1880. https://doi.org/10.1016/j.ymthe.2017.05.004.

25. Quinn, J., Salman, A., Paluch, C., Jackson-Wood, M., McClements, M.E., Luo, J., Davis, S.J., Cornall, R.J., MacLaren, R.E., Dendrou, C.A., and Xue, K. (2024). Single-cell transcriptomic analysis of retinal immune regulation and blood-retinal barrier function during experimental autoimmune uveitis. Sci. Rep. *14*, 20033. https://doi.org/10.1038/s41598-024-68401-y.

26. Wiley, L.A., Boyce, T.M., Meyering, E.E., Ochoa, D., Sheehan, K.M., Stone, E.M., Mullins, R.F., Tucker, B.A., and Han, I.C. (2023). The Degree of Adeno-Associated Virus-Induced Retinal Inflammation Varies Based on Serotype and Route of Delivery: Intravitreal, Subretinal, or Suprachoroidal. Hum. Gene Ther. *34*, 530–539. https://doi.org/10.1089/hum.2022.222.

27. Andreatta, M., Corria-Osorio, J., Müller, S., Cubas, R., Coukos, G., and Carmona, S.J. (2021). Interpretation of T cell states from single-cell transcriptomics data using reference atlases. Nat. Commun. *12*, 2965. https://doi.org/10.1038/s41467-021-23324-4.

28. Hallam, T.M., Gardenal, E., McBlane, F., Cho, G., Ferraro, L.L., Pekle, E., Lu, D., Carney, K., Wenden, C., Beadsmoore, H., et al. (2025). Ocular biomarker profiling after complement factor I gene therapy in geographic atrophy secondary to age-related macular degeneration. medRxiv. https://doi.org/10.7554/elife.99806.2.

29. von Krusenstiern, L., Liu, J., Liao, E., Gow, J.A., Chen, G., Ong, T., Lotery, A.J., Jalil, A., Lam, B.L., and MacLaren, R.E.; XIRIUS Part 1 Study GroupXOLARIS Study Group (2023). Changes in Retinal Sensitivity Associated With Cotoretigene Toliparvovec in X-Linked Retinitis Pigmentosa With RPGR Gene Variations. JAMA Ophthalmol. *141*, 275–283. https://doi.org/10.1001/jamaophthalmol.2022.6254.

30. Wright, R.N., Hong, D.H., and Perkins, B. (2011). Misexpression of the constitutive Rpgr(ex1-19) variant leads to severe photoreceptor degeneration. Investig. Ophthalmol. Vis. Sci. *52*, 5189–5201. https://doi.org/10.1167/iovs.11-7470.

31. Dufour, V.L., Cideciyan, A.V., Ye, G.J., Song, C., Timmers, A., Habecker, P.L., Pan, W., Weinstein, N.M., Swider, M., Durham, A.C., et al. (2020). Toxicity and Efficacy Evaluation of an Adeno-Associated Virus Vector Expressing Codon-Optimized RPGR Delivered by Subretinal Injection in a Canine Model of X-linked Retinitis Pigmentosa. Hum. Gene Ther. *31*, 253–267. https://doi.org/10.1089/hum.2019.297.

32. Song, C., Dufour, V.L., Cideciyan, A.V., Ye, G.J., Swider, M., Newmark, J.A., Timmers, A.M., Robinson, P.M., Knop, D.R., Chulay, J.D., et al. (2020). Dose Range Finding Studies with Two RPGR Transgenes in a Canine Model of X-Linked Retinitis Pigmentosa Treated with Subretinal Gene Therapy. Hum. Gene Ther. *31*, 743–755. https://doi.org/10.1089/hum.2019.337.

33. Stingl, K., Stingl, K., Schwartz, H., Reid, M.W., Kempf, M., Dimopoulos, S., Kortuem, F., Borchert, M.S., Lee, T.C., and Nagiel, A. (2023). Full-field Scotopic Threshold Improvement after Voretigene Neparvovec-rzyl Treatment Correlates with Chorioretinal Atrophy. Ophthalmology *130*, 764–770. https://doi.org/10.1016/j.ophtha.2023.02.015.

34. Weed, L., Ammar, M.J., Zhou, S., Wei, Z., Serrano, L.W., Sun, J., Lee, V., Maguire, A.M., Bennett, J., and Aleman, T.S. (2019). Safety of Same-Eye Subretinal Sequential Readministration of AAV2-*hRPE65v2* in Non-human Primates. Mol. Ther., Methods Clin. Dev. *15*, 133–148. https://doi.org/10.1016/j.omtm.2019.08.011.

35. Gange, W.S., Sisk, R.A., Besirli, C.G., Lee, T.C., Havunjian, M., Schwartz, H., Borchert, M., Sengillo, J.D., Mendoza, C., Berrocal, A.M., and Nagiel, A. (2022). Perifoveal Chorioretinal Atrophy after Subretinal Voretigene Neparvovec-rzyl for RPE65-Mediated Leber Congenital Amaurosis. Ophthalmol. Retina *6*, 58–64. https://doi.org/10.1016/j.oret.2021.03.016.

36. Bucher, K., Rodríguez-Bocanegra, E., Wissinger, B., Strasser, T., Clark, S.J., Birkenfeld, A.L., Siegel-Axel, D., and Fischer, M.D. (2023). Extra-viral DNA in adeno-associated viral vector preparations induces TLR9-dependent innate immune responses in human plasmacytoid dendritic cells. Sci. Rep. *13*, 1890. https://doi.org/10.1038/s41598-023-28830-7.

37. Reichel, F.F., Dauletbekov, D.L., Klein, R., Peters, T., Ochakovski, G.A., Seitz, I.P., Wilhelm, B., Ueffing, M., Biel, M., Wissinger, B., et al. (2017). AAV8 Can Induce Innate and Adaptive Immune Response in the Primate Eye. Mol. Ther. *25*, 2648–2660. https://doi.org/10.1016/j.ymthe.2017.08.018.

38. Rutar, M., Natoli, R., Chia, R.X., Valter, K., and Provis, J.M. (2015). Chemokine-mediated inflammation in the degenerating retina is coordinated by Müller cells, activated microglia, and retinal pigment epithelium. J. Neuroinflammation *12*, 8. https://doi.org/10.1186/s12974-014-0224-1.

39. Xiong, W., Wu, D.M., Xue, Y., Wang, S.K., Chung, M.J., Ji, X., Rana, P., Zhao, S.R., Mai, S., and Cepko, C.L. (2019). AAV cis-regulatory sequences are correlated with ocular toxicity. Proc. Natl. Acad. Sci. USA *116*, 5785–5794. https://doi.org/10.1073/pnas.1821000116.

40. Liu, J., Lei, F., Yan, B., Cao, T., Cui, N., Sharma, J., Correa, V., Roach, L., Nicolaou, S., Pitts, K., et al. (2025). Epigenetic adaptation drives monocyte differentiation into microglia-like cells upon engraftment into the retina. bioRxiv, 2024.09.09.612126. https://doi.org/10.1101/2024.09.09.612126.

41. Choi, J., Li, J., Ferdous, S., Liang, Q., Moffitt, J.R., and Chen, R. (2023). Spatial organization of the mouse retina at single cell resolution by MERFISH. Nat. Commun. *14*, 4929. https://doi.org/10.1038/s41467-023-40674-3.

42. Zhang, J., Wang, J., Zhou, Q., Chen, Z., Zhuang, J., Zhao, X., Gan, Z., Wang, Y., Wang, C., Molday, R.S., et al. (2025). Spatiotemporally resolved transcriptomics reveals the cellular dynamics of human retinal development. Nat. Commun. *16*, 2307. https://doi.org/10.1038/s41467-025-57625-9.

43. Xue, K., Groppe, M., Salvetti, A.P., and MacLaren, R.E. (2017). Technique of retinal gene therapy: delivery of viral vector into the subretinal space. Eye (Lond) *31*, 1308–1316. https://doi.org/10.1038/eye.2017.158.

44. Schindelin, J., Arganda-Carreras, I., Frise, E., Kaynig, V., Longair, M., Pietzsch, T., Preibisch, S., Rueden, C., Saalfeld, S., Schmid, B., et al. (2012). Fiji: an open-source platform for biological-image analysis. Nat. Methods *9*, 676–682. https://doi.org/10.1038/nmeth.2019.

45. Young, M.D., and Behjati, S. (2020). SoupX removes ambient RNA contamination from droplet-based single-cell RNA sequencing data. GigaScience *9*, giaa151. https://doi.org/10.1093/gigascience/giaa151.

46. Germain, P.L., Lun, A., Garcia Meixide, C., Macnair, W., and Robinson, M.D. (2021). Doublet identification in single-cell sequencing data using *scDblFinder*. F1000Res. *10*, 979. https://doi.org/10.12688/f1000research.73600.2.

47. Wolf, F.A., Angerer, P., and Theis, F.J. (2018). SCANPY: large-scale single-cell gene expression data analysis. Genome Biol. *19*, 15. https://doi.org/10.1186/s13059-017-1382-0.

48. Hao, Y., Stuart, T., Kowalski, M.H., Choudhary, S., Hoffman, P., Hartman, A., Srivastava, A., Molla, G., Madad, S., Fernandez-Granda, C., and Satija, R. (2024). Dictionary learning for integrative, multimodal and scalable single-cell analysis. Nat. Biotechnol. *42*, 293–304. https://doi.org/10.1038/s41587-023-01767-y.

49. Badia-I-Mompel, P., Vélez Santiago, J., Braunger, J., Geiss, C., Dimitrov, D., Müller-Dott, S., Taus, P., Dugourd, A., Holland, C.H., Ramirez Flores, R.O., and Saez-Rodriguez, J. (2022). decoupleR: ensemble of computational methods to infer biological activities from omics data. Bioinform. Adv. *2*, vbac016. https://doi.org/10.1093/bioadv/vbac016.

50. Love, M.I., Huber, W., and Anders, S. (2014). Moderated estimation of fold change and dispersion for RNA-seq data with DESeq2. Genome Biol. *15*, 550. https://doi.org/10.1186/s13059-014-0550-8.

51. Sergushichev, A.A. (2016). An algorithm for fast preranked gene set enrichment analysis using cumulative statistic calculation. bioRxiv, 060012. https://doi.org/10.1101/060012.

52. Faure, L., Soldatov, R., Kharchenko, P.V., and Adameyko, I. (2023). scFates: a scalable python package for advanced pseudotime and bifurcation analysis from single-cell data. Bioinformatics *39*, btac746. https://doi.org/10.1093/bioinformatics/btac746.

53. Fruchterman, T.M.J., and Reingold, E.M. (1991). Graph drawing by force-directed placement. Software Pract. Ex. *21*, 1129–1164. https://doi.org/10.1002/spe.4380211102.

54. Washer, S.J., Perez-Alcantara, M., Chen, Y., Steer, J., James, W.S., Trynka, G., Bassett, A.R., and Cowley, S.A. (2022). Single-cell transcriptomics defines an improved, validated monoculture protocol for differentiation of human iPSC to microglia. Sci. Rep. *12*, 19454. https://doi.org/10.1038/s41598-022-23477-2.

OMTA, Volume *34*

Supplemental information

Single-cell and spatial transcriptomic analyses of gene therapy-associated retinal inflammation in non-human primates

Célia Sourd, Joel Quinn, Molly C. John, Cristina Martinez-Fernandez de la Camara, Lakshanie C. Wickramasinghe, Moustafa Attar, Hoda Shamsnajafabadi, Ahmed Salman, Sally A. Cowley, Calliope A. Dendrou, Robert E. MacLaren, Jasmina Cehajic-Kapetanovic, and Kanmin Xue

Table S1. List of PCR primers.

Name	Sequence	Purpose
**mScarlet_F**	GCGTGATGAACTTCGAGGAC	qPCR Titration of Vector
**mScarlet_R**	CTTGTAGATCAGGGTGCCGT	qPCR Titration of Vector

Table S2. List of antibodies.

Target	Host	Clonality	Reference	Supplier	Working dilution
**RPGR**	Rabbit	Polyclonal	HPA001593	Sigma-Aldrich	1:200 (IHC)
**RPE65**	Mouse	Monoclonal	401.8B11.3D9	Novus Biologicals	1:250 (IHC)
**IBA1**	Rabbit	Monoclonal	019-19741	Wako	1:500 (IHC)
**GFAP**	Chicken	Polyclonal	Ab4674	Abcam	1:200 (IHC)
**CD45**	Rabbit	Monoclonal	Ab281586	Abcam	1:250 (IHC)

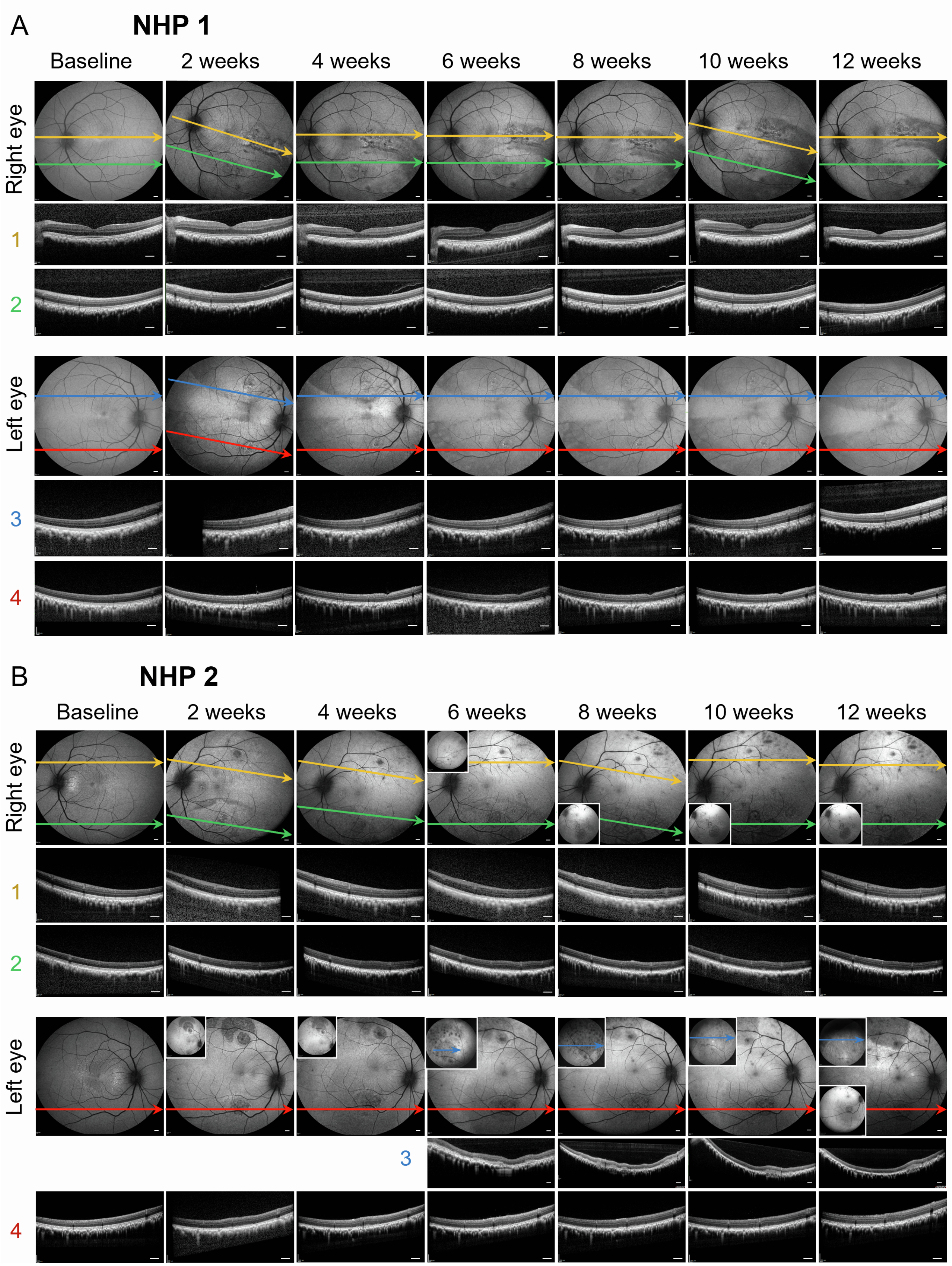
A
NHP 1
Baseline
2 weeks
4 weeks
6 weeks
8 weeks
10 weeks
12 weeks
Right eye
1
2
Left eye
3
4
B
NHP 2
Baseline
2 weeks
4 weeks
6 weeks
8 weeks
10 weeks
12 weeks
Right eye
1
2
Left eye
3
4

Figure S1. Longitudinal multimodal retinal imaging over 12 weeks following subretinal injection of AAV vectors in NHPs.

See Figure 2. Longitudinal multimodal retinal imaging of the macula following subretinal injection of AAV vectors over 12 weeks of both eyes of NHP1 (**A**) and NHP2 (**B**). The coloured lines represent the locations of OCT sections mapped to the fundus autofluorescence (AF) images from inside the treated subretinal bleb areas. Note that all retinal images are vertically inverted (top of the image representing inferior retina). Hypo-autofluorescent patches corresponding to the development of RPE/outer retinal atrophy can be seen along the inferior arcade of the left eye of NHP2 which was treated with an AAV8-GRK1-*mScarlet* vector. The surrounding region showed increased background hyper-autofluorescence which likely represents mScarlet reporter expression. Scale bars = 500 µm.

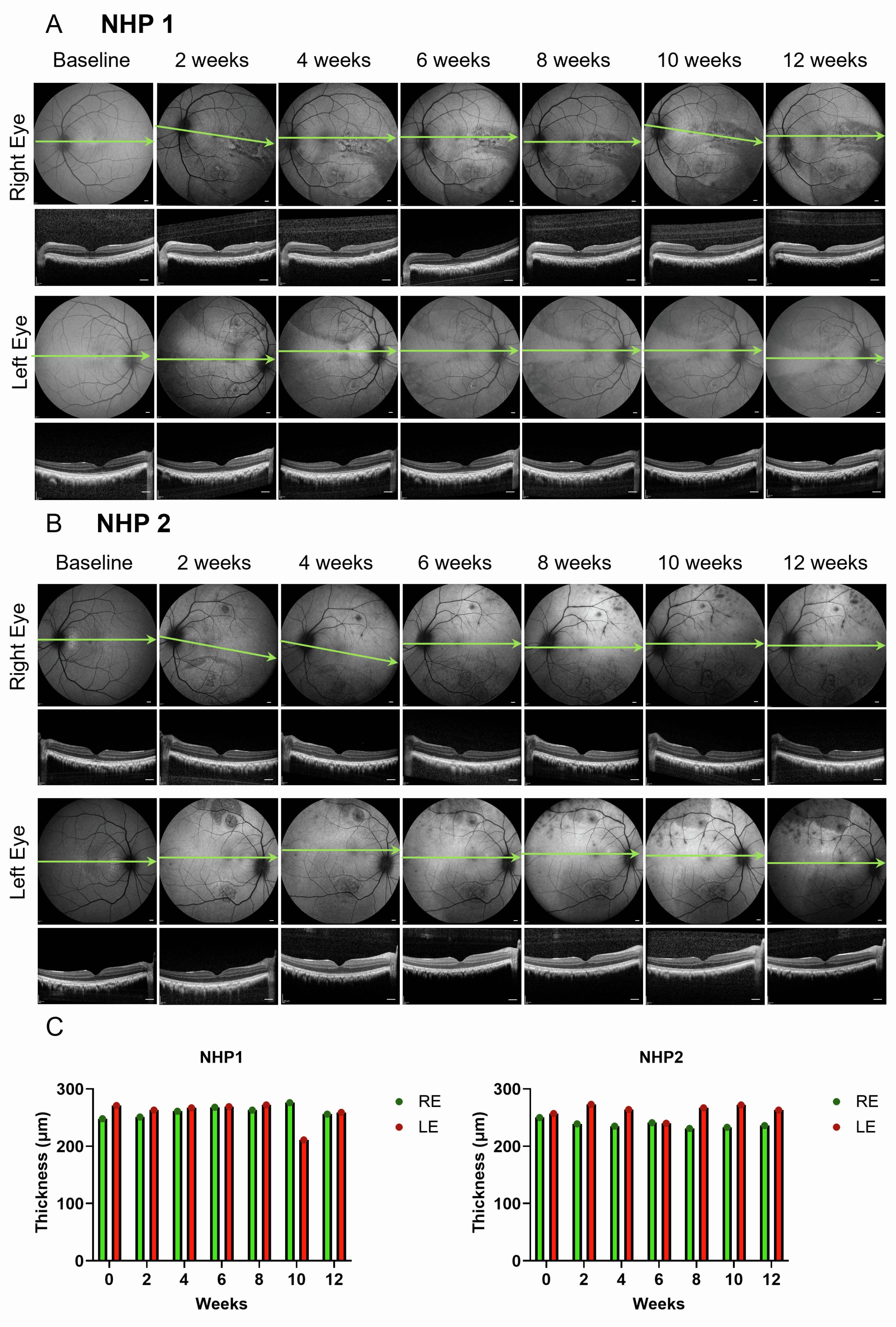
A NHP 1
Baseline
2 weeks
4 weeks
6 weeks
8 weeks
10 weeks
12 weeks
Right Eye
Left Eye
B NHP 2
Baseline
2 weeks
4 weeks
6 weeks
8 weeks
10 weeks
12 weeks
Right Eye
Left Eye
C
NHP1
NHP2
Thickness (μm)
300
200
100
0
0
2
4
6
8
10
12
Weeks
RE
LE

Figure S2. Preservation of macula anatomy following subretinal AAV gene therapy in NHPs.

Fundus autofluorescence (AF) and OCT imaging of the macula following subretinal injection of AAV vectors over 12 weeks of the right and left eyes of NHP1 (**A**) and NHP2 (**B**). Green lines on the AF images represent the localisations of corresponding OCT sections across the fovea. White bars represent a scale of 500μm. (**C**) Retinal thickness at the macula of NHP1 and NHP2 derived from the OCT images showed no significant changes over 12 weeks.

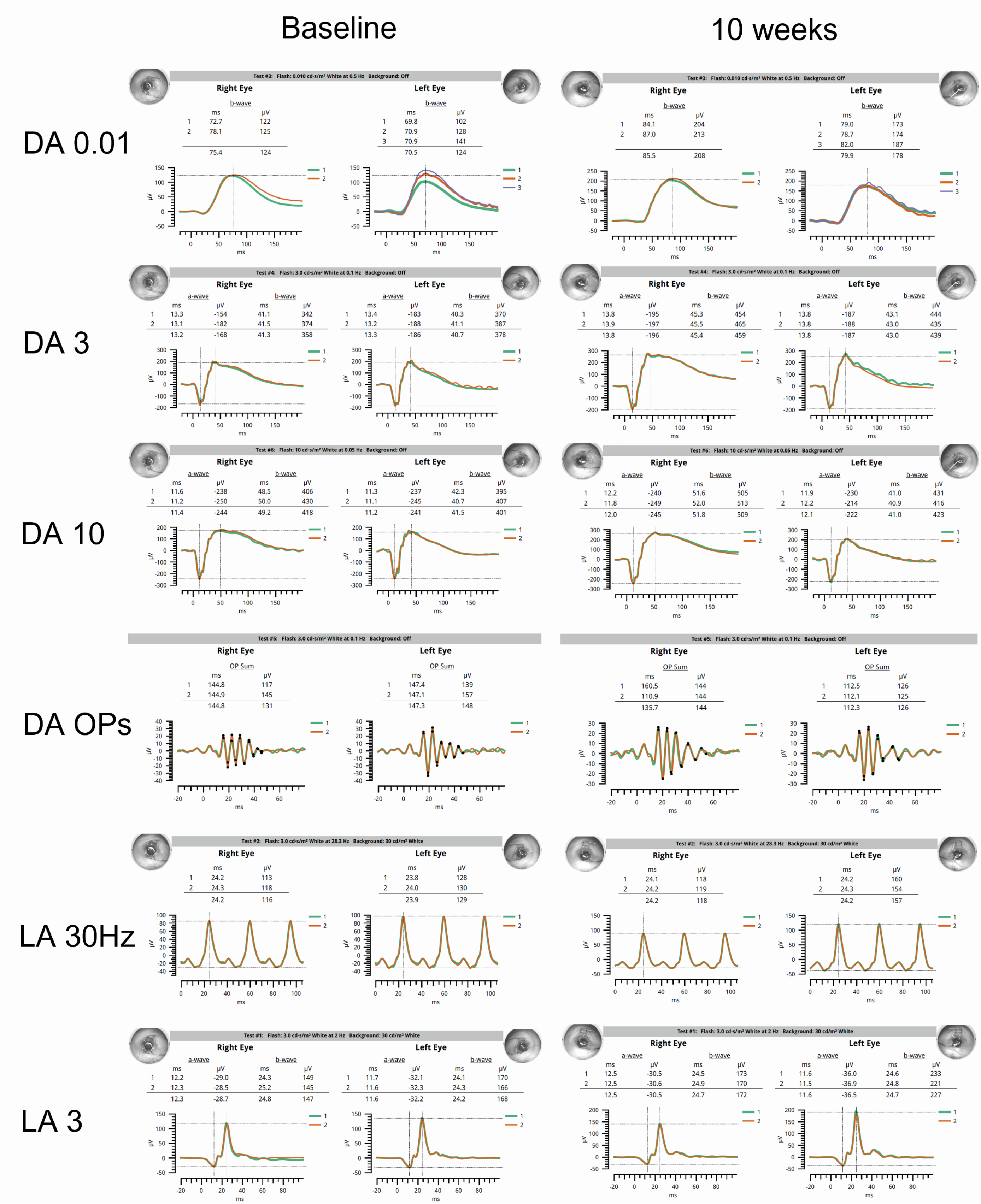
Baseline
10 weeks
DA 0.01
DA 3
DA 10
DA OPs
LA 30Hz
LA 3

Figure S3. Electroretinogram of NHP1.

Electroretinogram (ERG) responses recorded under dark-adapted (DA) and light-adapted (LA) conditions at baseline and 10 weeks after treatment in the right and left eyes of NHP1. Measurements included standard a-wave and b-wave responses, as well as oscillatory potentials (OPs) and 30 Hz flicker were performed in duplicates (green and orange lines) to assess retinal function.

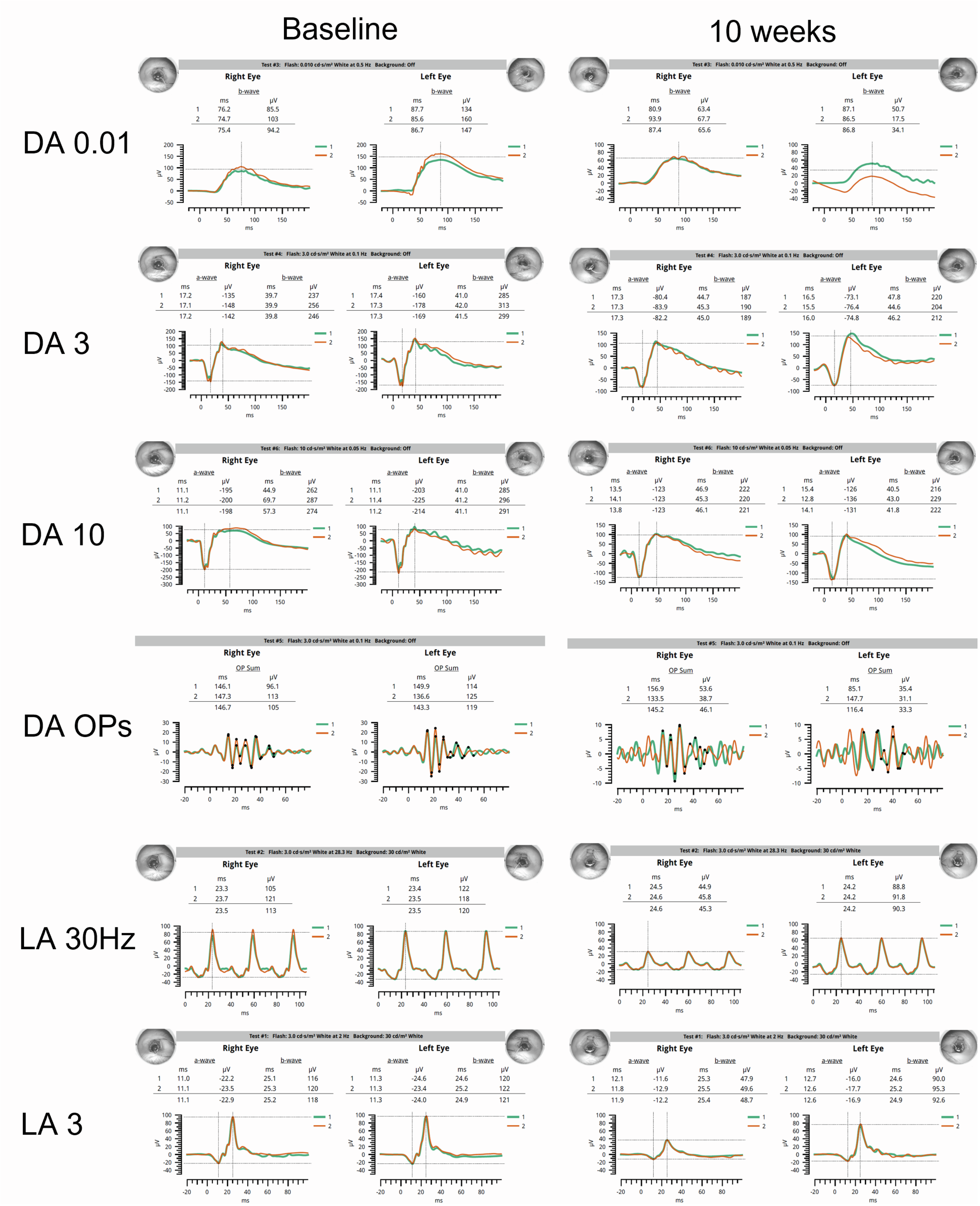
Baseline
10 weeks
DA 0.01
DA 3
DA 10
DA OPs
LA 30Hz
LA 3
Right Eye
Left Eye

Figure S4. Electroretinogram of NHP2.

Electroretinogram (ERG) responses recorded under dark-adapted (DA) and light-adapted (LA) conditions at baseline and 10 weeks after treatment in the right and left eyes of NHP2. Measurements included standard a-wave and b-wave responses, as well as oscillatory potentials (OPs) and 30 Hz flicker were performed in duplicates (green and orange lines) to assess retinal function.

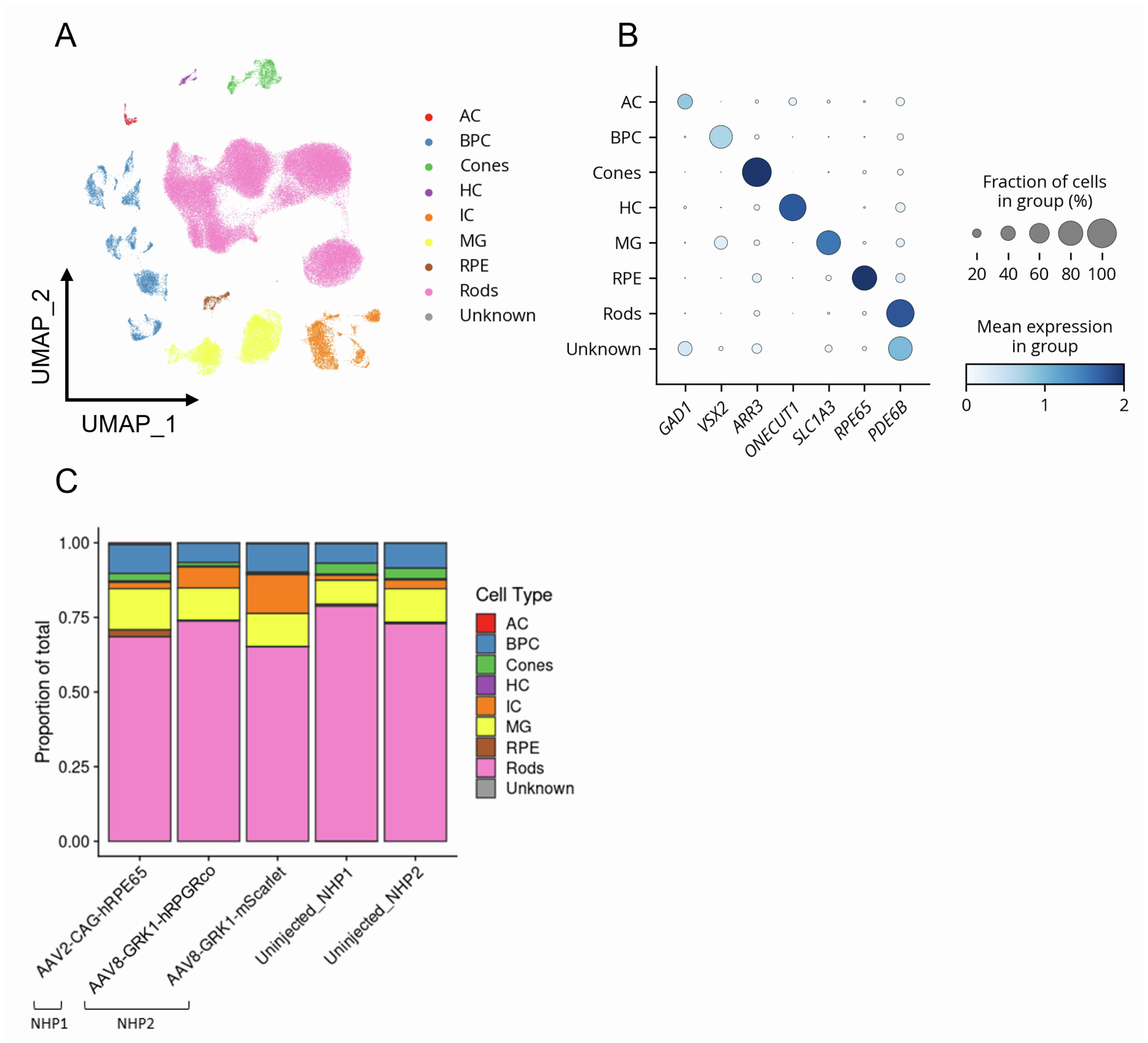

Figure S5. Single-cell transcriptomic analysis of retina cell populations in treated and untreated retinas.

Single-cell RNAseq data from dissociated retinas of NHP1 and NHP2 were combined. (**A**) Integrated UMAP of the entire cell population with labelled cell types. (**B**) Major marker genes used for cell type annotation. (**C**) Proportion of cell types identified from each AAV vector treated bleb area. AC = amacrine cells; BPC = bipolar cells; HC = horizontal cells; IC = immune cells; MG = Müller glia; RPE = retinal pigment epithelium.

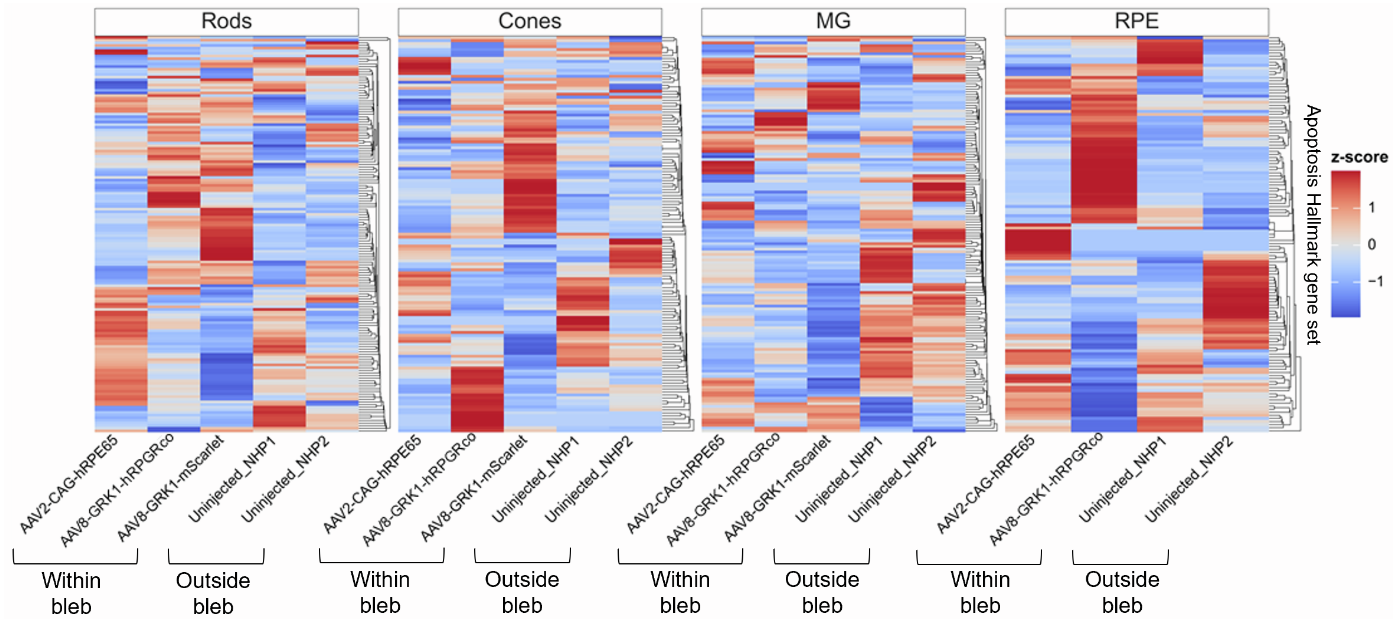

Figure S6. No clear correlation found between the levels of apoptotic marker gene expression and AAV treatment.

Normalised expression levels of apoptotic marker genes in the Apoptosis MSigDB Hallmark gene set in the rod, cone, Müller glia and RPE cell populations were compared between AAV treated (within bleb) versus untreated (outside bleb) retinas.

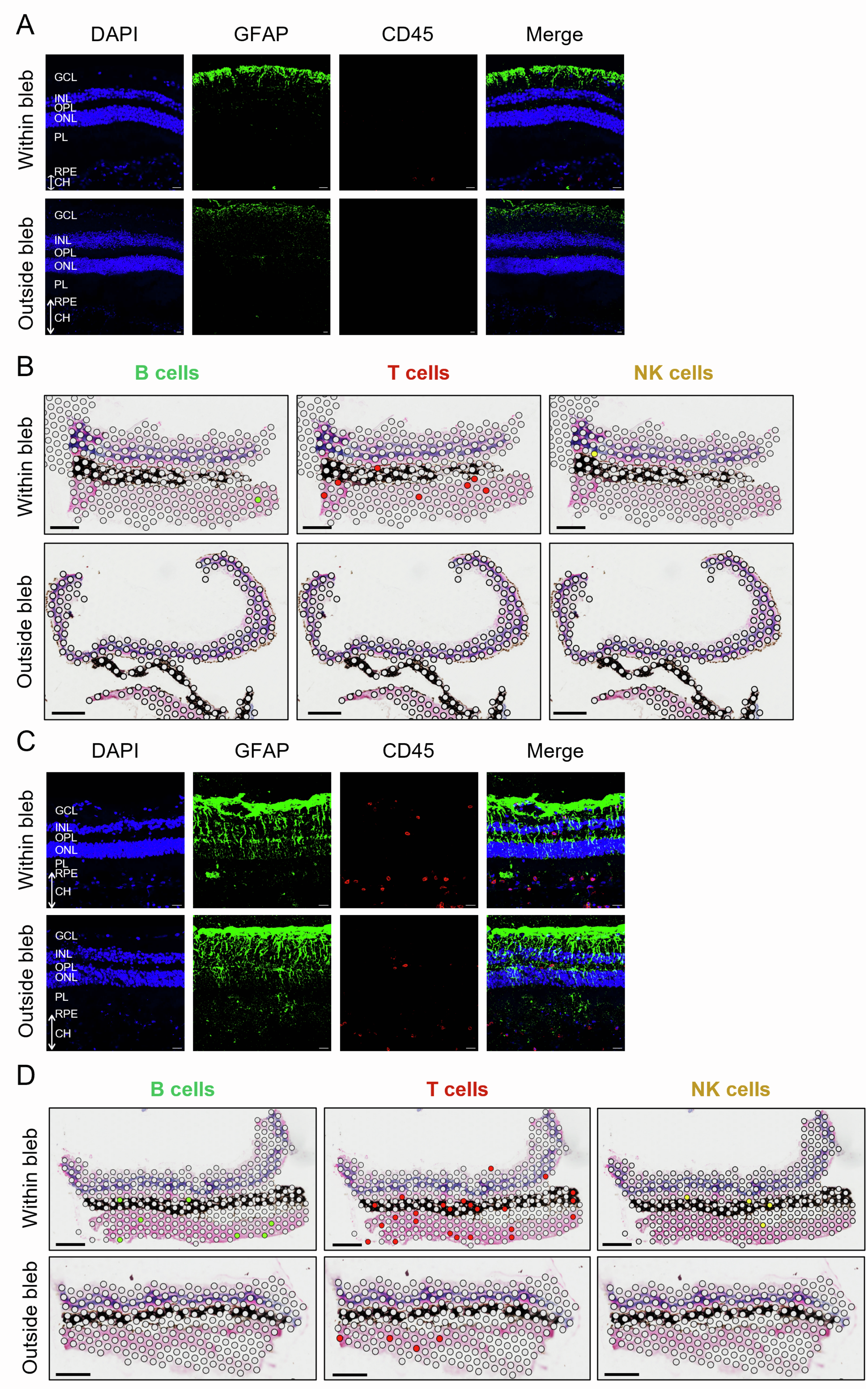
A
DAPI
GFAP
CD45
Merge
Within bleb
Outside bleb
GCL
INL
OPL
ONL
PL
RPE
CH
B
B cells
T cells
NK cells
C
D

Figure S7. Analysis of inflammation in the AAV-treated NHP retinas.

(**A, C**) Immunostaining of retina sections from (**A**) AAV2-CAG-*hRPE65* (NHP1) and (**C**) AAV8-GRK1-*hRPGRco* (NHP2) for GFAP and CD45 proteins. Control sections were taken from outside the treated blebs. Scale bars = 20µm. GCL = ganglion cell layer; INL = inner nuclear layer; OPL = outer plexiform layer; ONL = outer nuclear layer; PL = photoreceptor layer; RPE = retinal pigmented epithelium. (**B, D**) Spatial transcriptomic maps of B cell, T cell and natural killer (NK) cell clusters within retina sections of NHP1 (**B**) and NHP2 (**D**). Coloured spots represent the locations of cells expressing marker genes of interest overlayed on the H&E staining image. Scale bar = 0.5 mm.

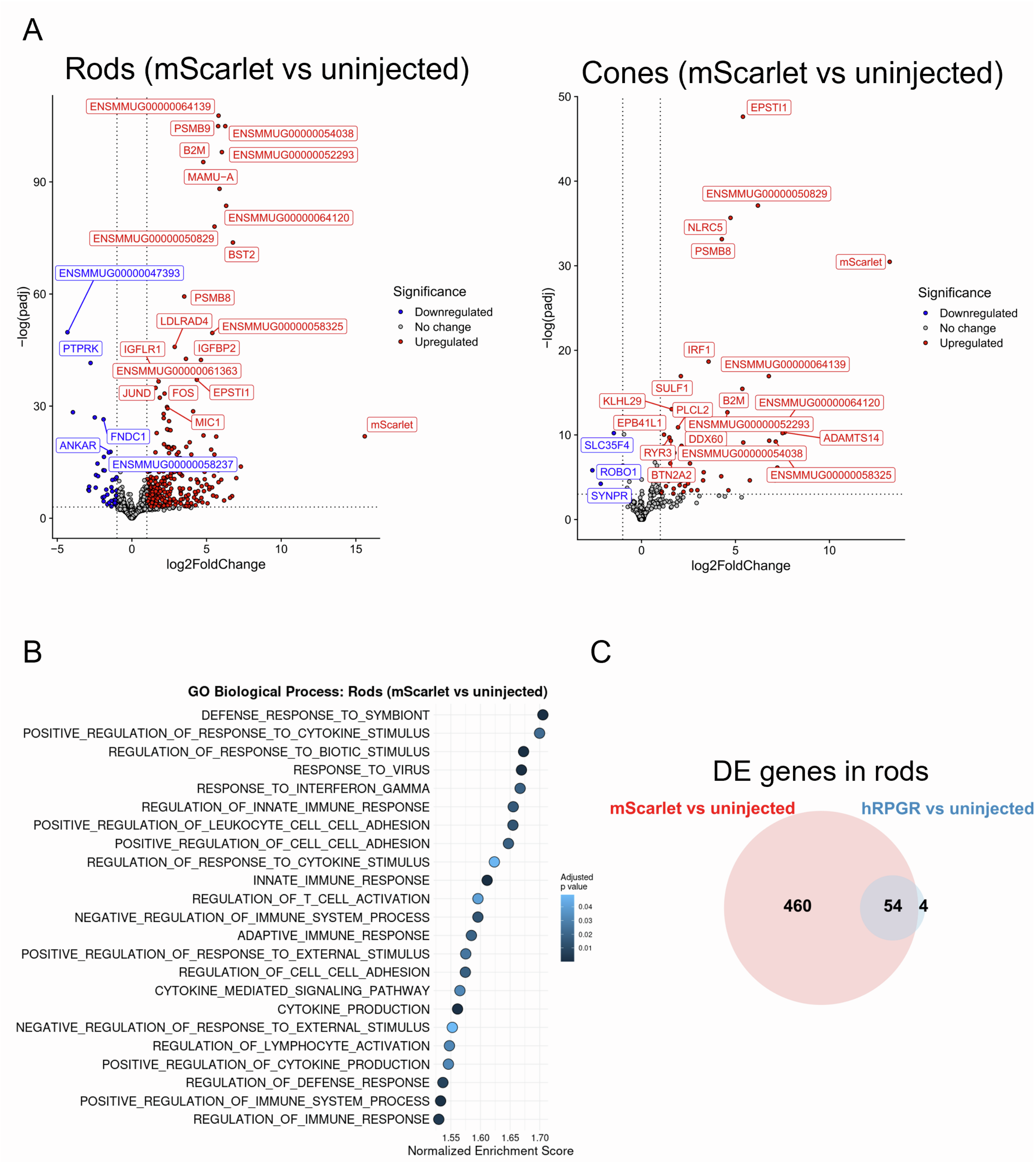
A
Rods (mScarlet vs uninjected)
Cones (mScarlet vs uninjected)
−log(padj)
log2FoldChange
Significance
Downregulated
No change
Upregulated
ENSMMUG00000064139
PSMB9
ENSMMUG00000054038
B2M
ENSMMUG00000052293
MAMU−A
ENSMMUG00000064120
ENSMMUG00000050829
BST2
ENSMMUG00000047393
PSMB8
LDLRAD4
ENSMMUG00000058325
PTPRK
IGFLR1
IGFBP2
ENSMMUG00000061363
JUND
FOS
EPSTI1
MIC1
mScarlet
FNDC1
ANKAR
ENSMMUG00000058237
EPSTI1
ENSMMUG00000050829
NLRC5
PSMB8
mScarlet
IRF1
ENSMMUG00000064139
SULF1
KLHL29
B2M
ENSMMUG00000064120
PLCL2
EPB41L1
ENSMMUG00000052293
ADAMTS14
SLC35F4
DDX60
RYR3
ENSMMUG00000054038
ROBO1
BTN2A2
ENSMMUG00000058325
SYNPR
B
GO Biological Process: Rods (mScarlet vs uninjected)
DEFENSE_RESPONSE_TO_SYMBIONT
POSITIVE_REGULATION_OF_RESPONSE_TO_CYTOKINE_STIMULUS
REGULATION_OF_RESPONSE_TO_BIOTIC_STIMULUS
RESPONSE_TO_VIRUS
RESPONSE_TO_INTERFERON_GAMMA
REGULATION_OF_INNATE_IMMUNE_RESPONSE
POSITIVE_REGULATION_OF_LEUKOCYTE_CELL_CELL_ADHESION
POSITIVE_REGULATION_OF_CELL_CELL_ADHESION
REGULATION_OF_RESPONSE_TO_CYTOKINE_STIMULUS
INNATE_IMMUNE_RESPONSE
REGULATION_OF_T_CELL_ACTIVATION
NEGATIVE_REGULATION_OF_IMMUNE_SYSTEM_PROCESS
ADAPTIVE_IMMUNE_RESPONSE
POSITIVE_REGULATION_OF_RESPONSE_TO_EXTERNAL_STIMULUS
REGULATION_OF_CELL_CELL_ADHESION
CYTOKINE_MEDIATED_SIGNALING_PATHWAY
CYTOKINE_PRODUCTION
NEGATIVE_REGULATION_OF_RESPONSE_TO_EXTERNAL_STIMULUS
REGULATION_OF_LYMPHOCYTE_ACTIVATION
POSITIVE_REGULATION_OF_CYTOKINE_PRODUCTION
REGULATION_OF_DEFENSE_RESPONSE
POSITIVE_REGULATION_OF_IMMUNE_SYSTEM_PROCESS
REGULATION_OF_IMMUNE_RESPONSE
Adjusted p value
Normalized Enrichment Score
C
DE genes in rods
mScarlet vs uninjected
hRPGR vs uninjected
460
54
4

Figure S8. Upregulation of antiviral and MHC Class I genes in NHP photoreceptors after AAV gene therapy.

Results derived from single-cell RNAseq analysis of dissociated retinas from NHP2. (**A**) DESeq2 differential expression analysis. Differentially upregulated genes include B2M (MHC I light chain), ENSMMUG00000054038 (macaque MHC I antigen), ENSMMUG00000064120 (ortholog to CD1D, MHC-like lipid antigen presenter), ENSMMUG00000050829 (MHC I pathway regulator), ENSMMUG00000052293 (ortholog to KIAA1109, MHC I complex assembly) and ENSMMUG00000058325 (MHC I antigen), indicating upregulated MHC class I antigen presentation. Upregulation of multiple immune system-associated genes in rods and cones from AAV8-GRK1-*mScarlet*-treated bleb versus untreated (outside of bleb) retina sections. (**B**) Gene Ontology (GO) Biological Process enrichment of upregulated genes in rods. A variety of antiviral and other immune-related gene sets in AAV8-GRK1-mScarlet treated bleb areas can be observed when compared with untreated (outside of bleb) retina sections. (**C**) Venn diagram comparing differentially expressed genes in rods between AAV8-GRK1-*hRPGRco* and AAV8-GRK1-*mScarlet* treated retinas. The majority of upregulated genes in rods from AAV8-GRK1-*hRPGRco* treated retina overlapped with those from AAV8-GRK1-*mScarlet* treated retina, suggesting a similar response. DE genes = differently expressed genes.

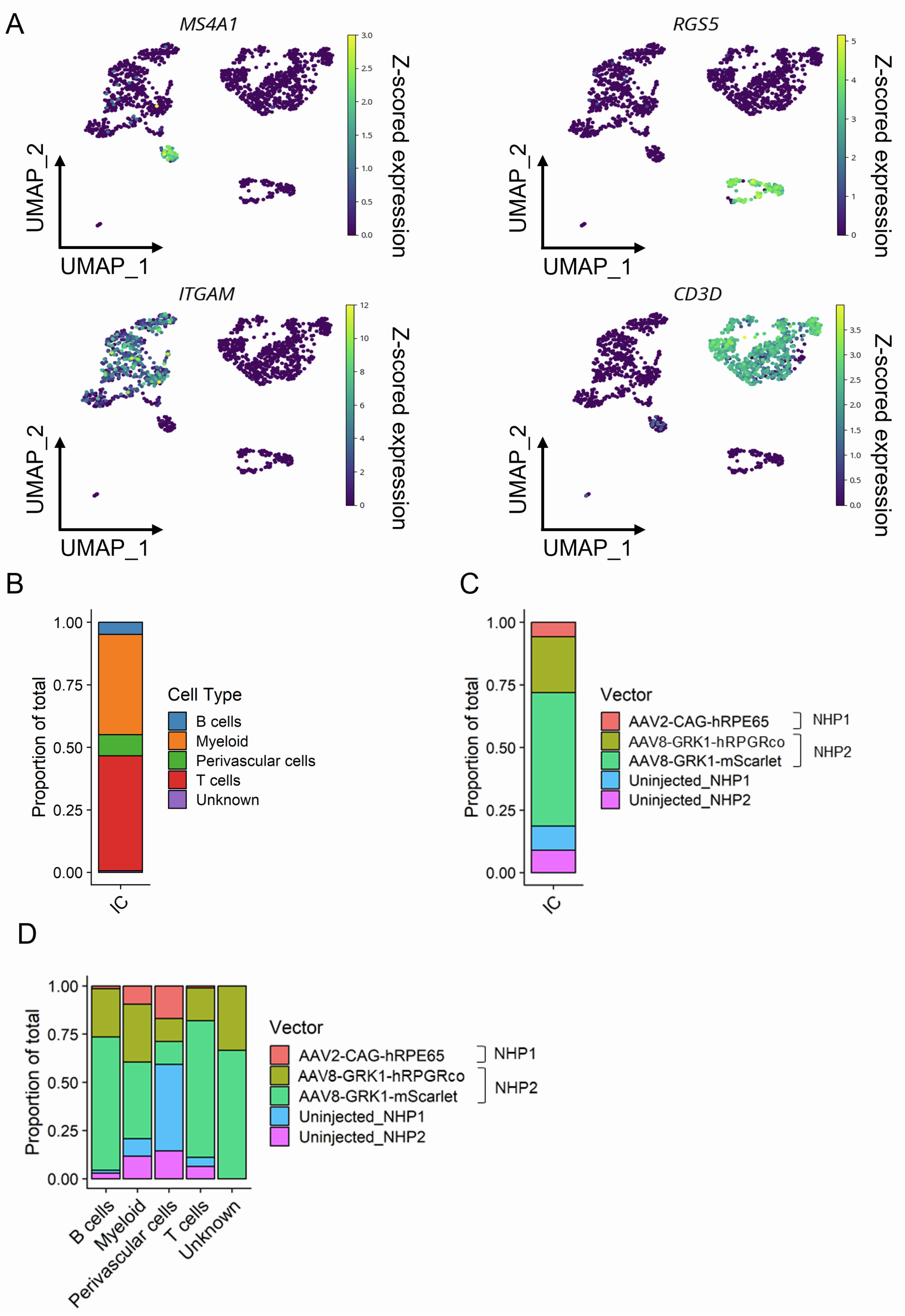
A
MS4A1
RGS5
ITGAM
CD3D
UMAP_1
UMAP_2
Z-scored expression
B
Proportion of total
1.00
0.75
0.50
0.25
0.00
IC
Cell Type
B cells
Myeloid
Perivascular cells
T cells
Unknown
C
Vector
AAV2-CAG-hRPE65
AAV8-GRK1-hRPGRco
AAV8-GRK1-mScarlet
Uninjected_NHP1
Uninjected_NHP2
NHP1
NHP2
D
B cells
Myeloid
Perivascular cells
T cells
Unknown

Figure S9. The immune cell population in AAV-injected NHP retinas.

See Figure 5 A. (**A**) Marker genes used to identify B cells (*MS4A1*), perivascular cells (*RGS5*), myeloid cells (*ITGAM*) and T cells (*CD3D*). Data from both NHP1 and NHP2. (**B**) Relative proportions of different immune cell populations showing predominantly myeloid and T cell infiltrate. (**C**) Relative contributions of immune cells from each retina sample showing that AAV8-GRK1-*mScarlet* and AAV8-GRK1-*hRPGRco* treated retinas contributed the majority of immune cells. (**D**) Relative contributions from different retina samples for each immune cell type. The majority of B cells, T cells and myeloid cells came from AAV8-GRK1-*mScarlet* and AAV8-GRK1-*hRPGRco* treated retinas, indicating ongoing adaptive immune response in the treated retina of NHP2.

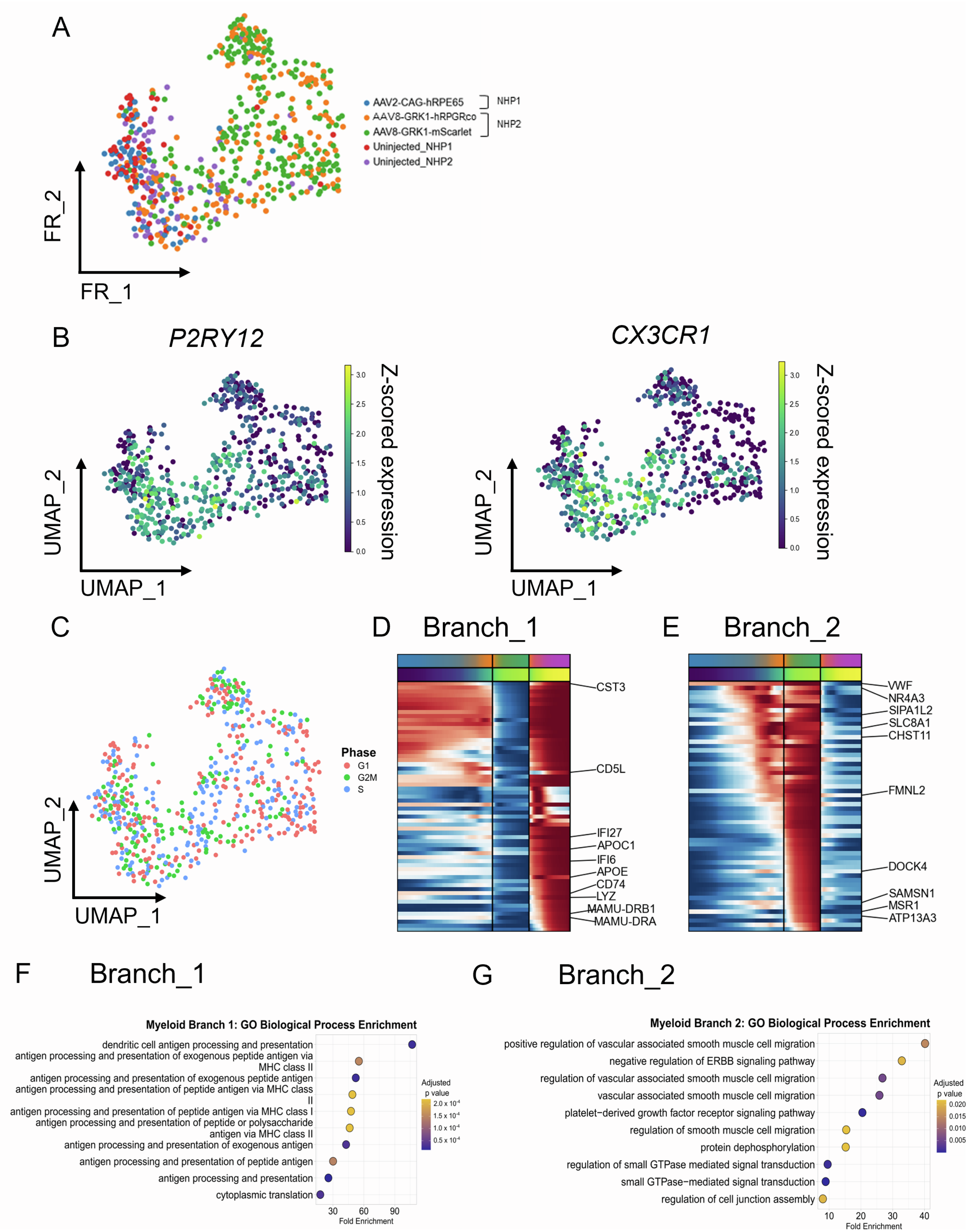
A
AAV2-CAG-hRPE65
AAV8-GRK1-hRPGRco
AAV8-GRK1-mScarlet
Uninjected_NHP1
Uninjected_NHP2
NHP1
NHP2
FR_1
FR_2
B
P2RY12
CX3CR1
Z-scored expression
UMAP_1
UMAP_2
C
Phase
G1
G2M
S
D Branch_1
CST3
CD5L
IFI27
APOC1
IFI6
APOE
CD74
LYZ
MAMU-DRB1
MAMU-DRA
E Branch_2
VWF
NR4A3
SIPA1L2
SLC8A1
CHST11
FMNL2
DOCK4
SAMSN1
MSR1
ATP13A3
F Branch_1
Myeloid Branch 1: GO Biological Process Enrichment
dendritic cell antigen processing and presentation
antigen processing and presentation of exogenous peptide antigen via MHC class II
antigen processing and presentation of exogenous peptide antigen
antigen processing and presentation of peptide antigen via MHC class II
antigen processing and presentation of peptide antigen via MHC class I
antigen processing and presentation of peptide or polysaccharide antigen via MHC class II
antigen processing and presentation of exogenous antigen
antigen processing and presentation of peptide antigen
antigen processing and presentation
cytoplasmic translation
Fold Enrichment
Adjusted p value
G Branch_2
Myeloid Branch 2: GO Biological Process Enrichment
positive regulation of vascular associated smooth muscle cell migration
negative regulation of ERBB signaling pathway
regulation of vascular associated smooth muscle cell migration
vascular associated smooth muscle cell migration
platelet−derived growth factor receptor signaling pathway
regulation of smooth muscle cell migration
protein dephosphorylation
regulation of small GTPase mediated signal transduction
small GTPase−mediated signal transduction
regulation of cell junction assembly
Fold Enrichment
Adjusted p value

Figure S10. Characterization of the myeloid cell population in AAV-treated NHP retinas.

See Figure 5 B-C. Data based on single-cell RNAseq analysis of retina samples from both NHP1 and NHP2. (**A**) 2-dimensional FR force-directed graph of myeloid cells coloured by treatment conditions. (**B**) Expression of microglia homeostatic markers, *P2RY12* and *CX3CR1*, identified the root for pseudotime analysis. (**C**) Myeloid cells coloured by cell cycle phases. No obvious proliferating cell cluster was detected. (**D**) Heatmap of significant Branch 1-specific gene expression over pseudotime. Detected MHC class I genes were previously observed in Figure S7 A. Detected MHC Class II genes included *Mamu-DRB1* (MHC II beta chain), *Mamu-DRA* (MHC class II alpha chain), *Mamu-DMB* (MHC class II peptide loader), *ENSMMUG00000056183* (ortholog to *Mamu-DQB1*, MHC II DQ beta chain) and *ENSMMUG00000019371* (ortholog to *Mamu-DQA1*, MHC II DQ alpha chain). Detected genes involved in antiviral defence included *RNASE6* (degrades viral RNA), *FGL2* (suppresses immune response, can dampen viral immune response), *BST2* (blocks virus release), *IFI27* (inhibits viral replication), *IFI6* (prevents virus-induced apoptosis), *NPC2* (regulates lipid homeostasis, affecting viral entry and replication) and *STING1* (activates interferon response). Genes commonly associated with pro-inflammatory myeloid cells such as *APOE* (inflammation marker), *APOC1* (modules immune response), *LYZ* (Bacterial defence enzyme), *FCER1G* (activates immune cells) and *C1QB* (complement system component) were also upregulated in Branch 1. (**E**) Heatmap of significant Branch 2-specific gene expression over pseudotime. Upregulated genes were *MAP4K4* (regulates cell migration), *NR4A3* (transcription factor, migration), *MERTK* (phagocytosis and mobility), *MYO1E* (actin-based cell movement), *PTPN1* (tyrosine phosphatase, signalling), *PTPRJ* (regulates cell adhesion and mobility) and *CBLB* (ubiquitin ligase, immune signalling). (**F**, **G**) Gene ontology (GO) enrichment analysis on Branch 1 (**F**) and Branch 2 (**G**) specific genes. The majority of Branch_1 detected genes are involved in antigen presentation. Branch_2 genes are involved in cell migration and mobility, GTPase activity and protein dephosphorylation.

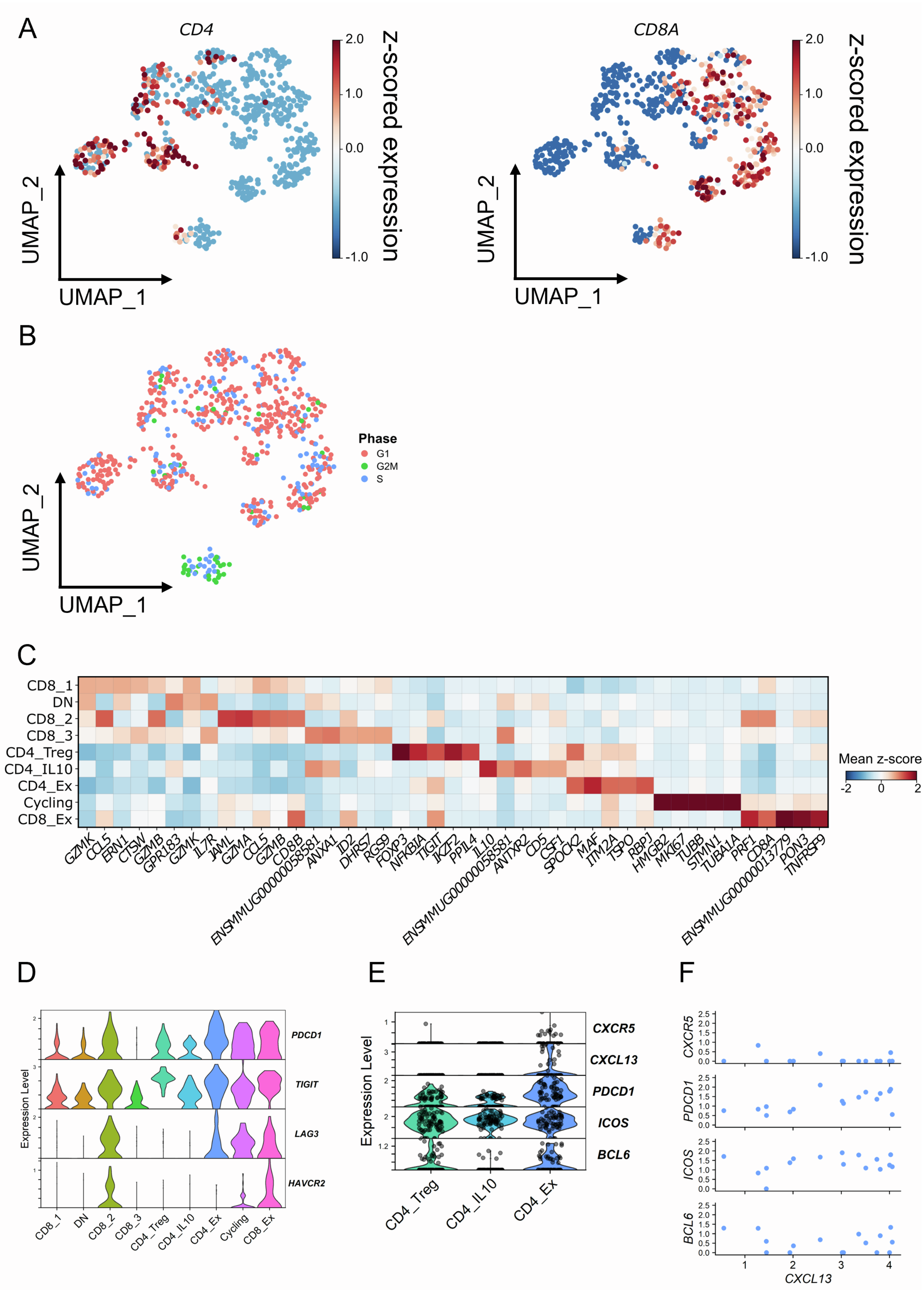

A
CD4
CD8A
Z-scored expression
2.0
1.0
0.0
-1.0
UMAP_2
UMAP_1
B
Phase
G1
G2M
S
UMAP_2
UMAP_1
C
CD8_1
DN
CD8_2
CD8_3
CD4_Treg
CD4_IL10
CD4_Ex
Cycling
CD8_Ex
GZMK CCL5 ERN1 CTSW GZMB GPR183 GZMK IL7R JAML GZMA CCL5 GZMB CD8B ENSMMUG00000058581 ANXA1 ID2 DHRS7 RGS9 FOXP3 NFKBIA TIGIT IKZF2 PPIL4 IL10 ENSMMUG00000058581 ANTXR2 CD5 CSF1 SPOCK2 MAF ITM2A TSPO RBPJ HMGB2 MKI67 TUBB STMN1 TUBA1A PRF1 CD8A ENSMMUG00000013779 PON3 TNFRSF9
Mean z-score
-2
0
2
D
Expression Level
PDCD1
TIGIT
LAG3
HAVCR2
CD8_1
DN
CD8_2
CD8_3
CD4_Treg
CD4_IL10
CD4_Ex
Cycling
CD8_Ex
E
Expression Level
CXCR5
CXCL13
PDCD1
ICOS
BCL6
CD4_Treg
CD4_IL10
CD4_Ex
F
CXCR5
PDCD1
ICOS
BCL6
CXCL13

Figure S11. Characterization of the T cell population in AAV-treated NHP retinas.

See Figure 5 D-F. Data based on single-cell RNAseq analysis of retina samples from both NHP1 and NHP2. (**A**) CD4 and CD8A marker gene expression enabled differentiation between CD4+ and CD8+ T cells. (**B**) Cells coloured by cell cycle phases, highlighting a significant cluster of proliferating T cells (in S phase). (**C**) Heatmap of major T cell subsets detected based on gene expression profiles. (**D**) Violin plots depicting expression of major immune checkpoint receptor genes (*PDCD1, TIGIT, LAG3 and HAVCR2*). Increased expression was seen in the 'CD8_2' T cell cluster and an exhausted CD8 T cell cluster ('CD8_Ex'). (**E**) Violin plots showing the expression of typical T follicular helper (Tfh) cell marker genes in CD4 clusters. (**F**) Scatter plots showing expression of Tfh marker genes against *CXCL13* in *CXCL13*$^+$ *CD4*$^+$ cells. Co-expression of *PDCD1*, *ICOS* and *BCL6*, but low expression of *CXCR5* might suggest a composition of T peripheral helper cells. (**D**) Violin plots depicting expression of major immune checkpoint receptor genes (*PDCD1, TIGIT, LAG3 and HAVCR2*). Increased expression was seen in the 'CD8_2' T cell cluster and an exhausted CD8 T cell cluster ('CD8_Ex'). (**E**) Violin plots showing the expression of typical T follicular helper (Tfh) cell marker genes in CD4 clusters. (**F**) Scatter plots showing expression of Tfh marker genes against *CXCL13* in *CXCL13*$^+$ *CD4*$^+$ cells. Co-expression of *PDCD1*, *ICOS* and *BCL6* but low expression of *CXCR5* might suggest a composition of T peripheral helper cells.

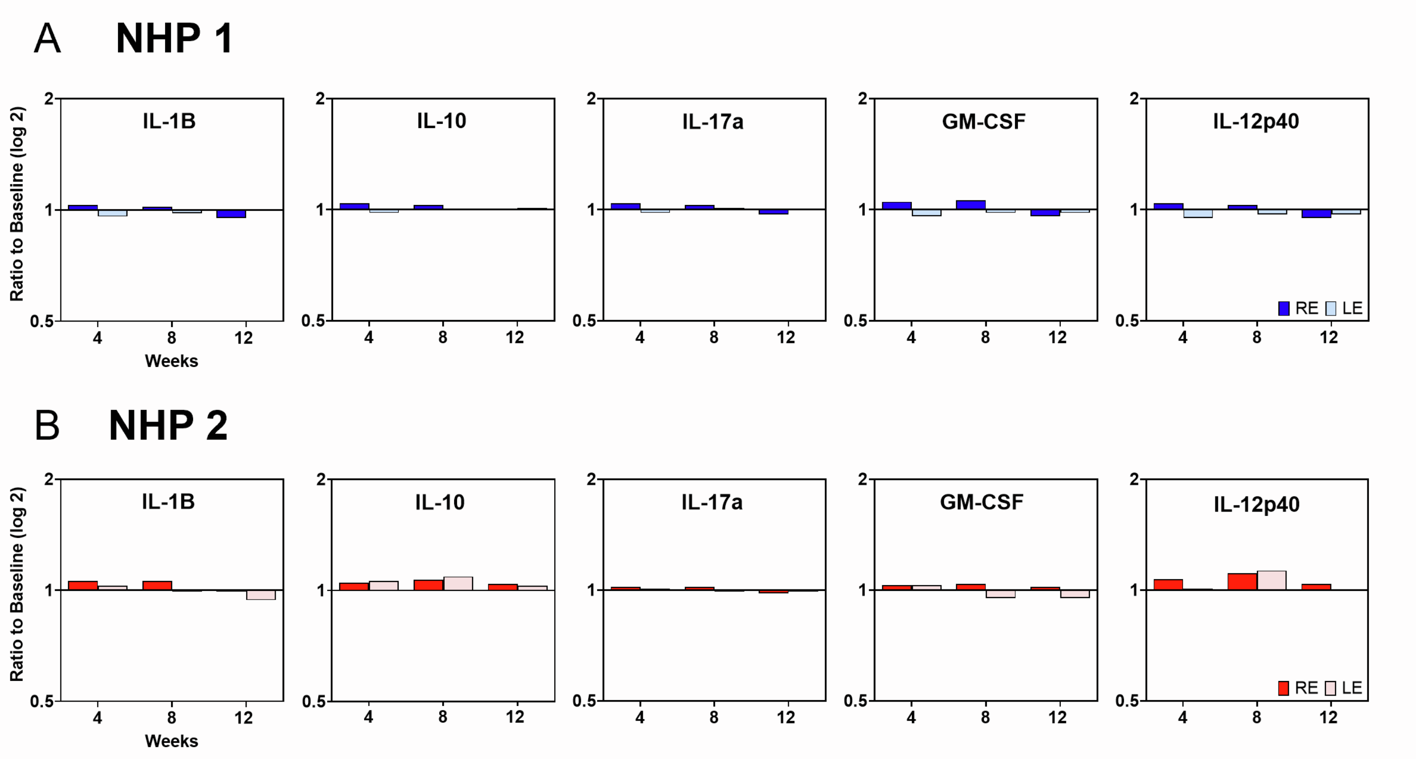

Figure S12. Additional cytokine panels for NHP vitreous samples.

See Figure 6. Additional cytokines assayed as part of the LegendPlex NHP Inflammation Panel applied to the vitreous samples from NHP1 (**A**) and NHP2 (**B**). Ratio scale is in log 2 compared to baseline.

A NHP 1

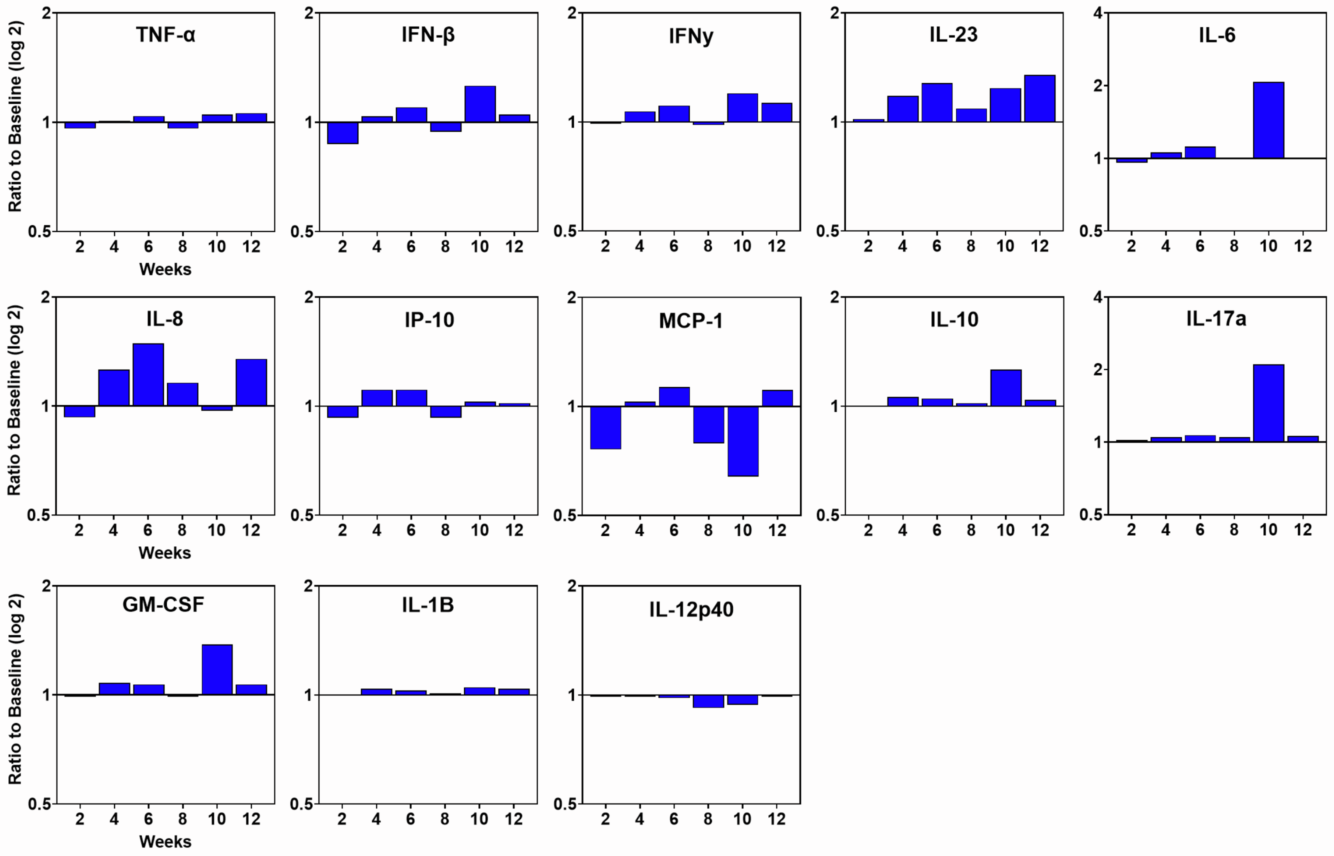

TNF-α
IFN-β
IFNγ
IL-23
IL-6
IL-8
IP-10
MCP-1
IL-10
IL-17a
GM-CSF
IL-1B
IL-12p40
Ratio to Baseline (log 2)
Weeks

B NHP 2

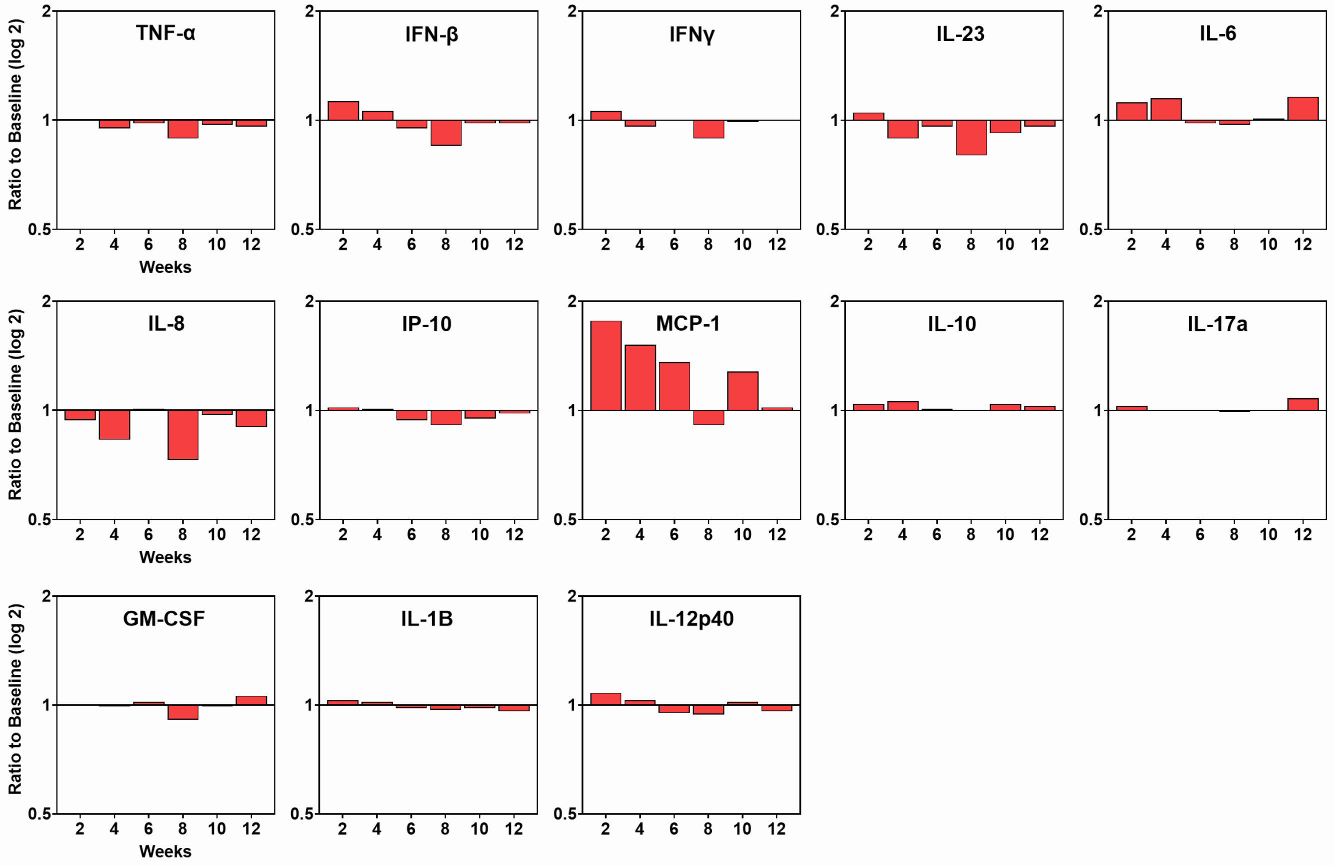

TNF-α
IFN-β
IFNγ
IL-23
IL-6
IL-8
IP-10
MCP-1
IL-10
IL-17a
GM-CSF
IL-1B
IL-12p40
Ratio to Baseline (log 2)
Weeks

Figure S13. Cytokine profiling of NHP peripheral blood mononuclear cells (PBMCs) revealed no major changes.

Analysis of cytokine expression in the blood samples from NHP1 (**A**) and NHP2 (**B**) following subretinal AAV gene therapy. Ratio scale is in log 2 compared to baseline.

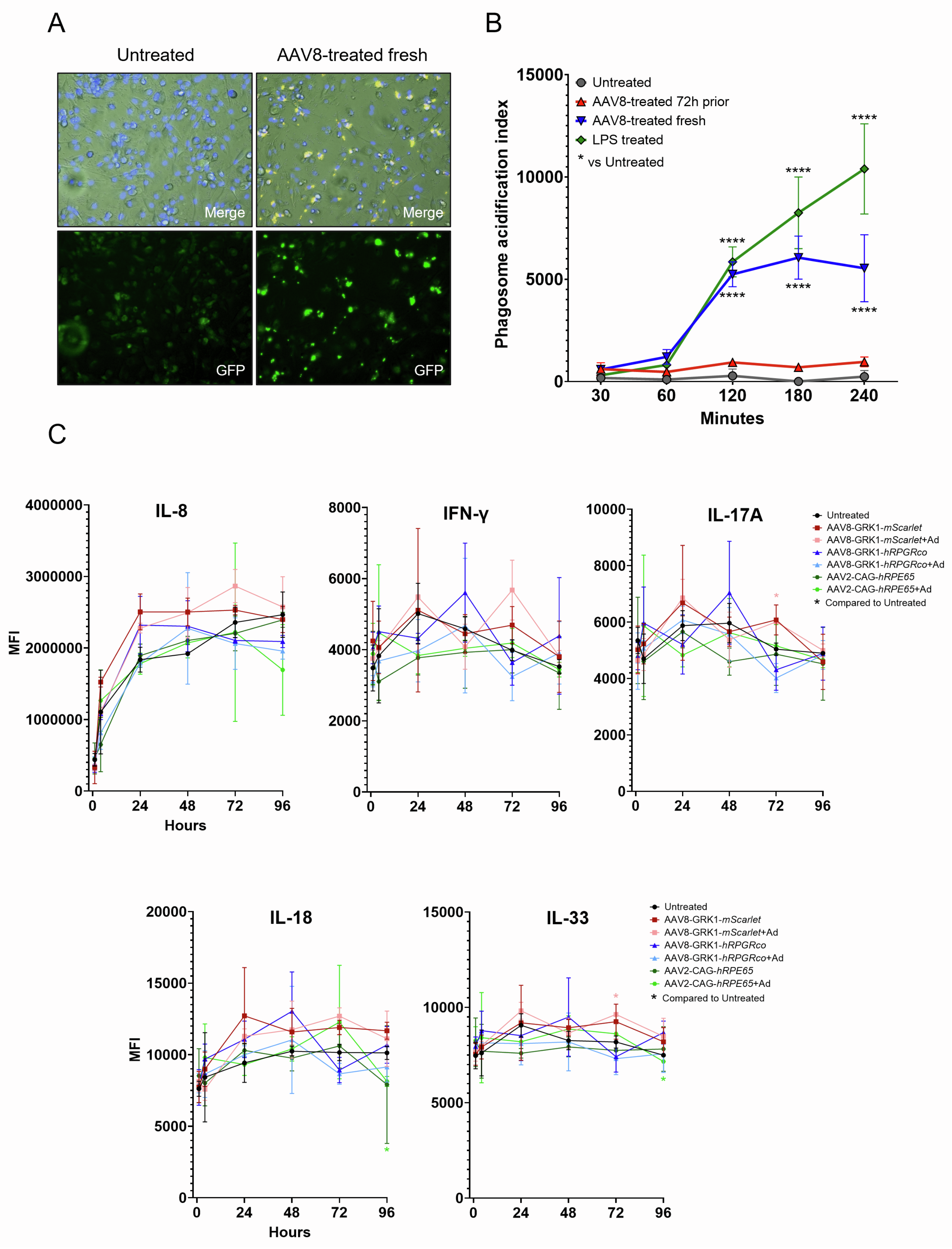

A
Untreated
AAV8-treated fresh
Merge
Merge
GFP
GFP
B
Phagosome acidification index
Untreated
AAV8-treated 72h prior
AAV8-treated fresh
LPS treated
* vs Untreated
****
Minutes
C
IL-8
IFN-γ
IL-17A
IL-18
IL-33
MFI
Hours
Untreated
AAV8-GRK1-mScarlet
AAV8-GRK1-mScarlet+Ad
AAV8-GRK1-hRPGRco
AAV8-GRK1-hRPGRco+Ad
AAV2-CAG-hRPE65
AAV2-CAG-hRPE65+Ad
* Compared to Untreated

Figure S14. Human iPSC-derived microglia activity and cytokine profile following exposure to AAV vectors.

See Figure 7. (**A**) Representative fluorescence microscopy images showing phagocytosis of pHrodo-labelled zymosan particles by iPSC-microglia exposed to AAV8-CAG-*mScarlet* (AAV8) at 240 min. pHrodo becomes fluorescent upon acidification within endosomes. (**B**) Phagocytic assay comparing (i) naïve microglia exposed to lipopolysaccharide (LPS) (green), (ii) naïve microglia exposed to AAV8-CAG-*mScarlet* (blue), (iii) microglia pre-treated with AAV8 72 hours prior (red), and (iv) untreated naïve microglia (grey). The phagosome acidification index was calculated as the total amount of fluorescence signal above threshold over the number of cells. (**C**) Rest of the cytokine expression panel applied to human iPSC-derived microglia after AAV stimulation. Data are represented as mean ± SD (n=4). Two-way ANOVA test was performed. Ad = adalimumab treatment. * = vs Untreated (UT). * = p>0.01.

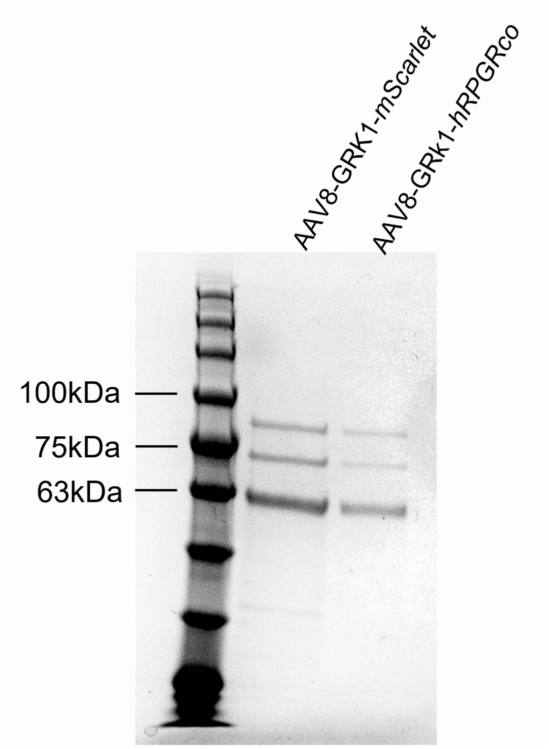

Figure S15. Comparison between purity of AAV8-GRK1-hRPGRco and AAV8-GRK1-mScarlet vector preparations.

Coomassie stained SDS-PAGE of equivalent dose-normalised amounts of both vectors.
